# Collagen hybridizing peptide imaging and delivery of therapeutic antibody in rheumatoid arthritis models

**DOI:** 10.1038/s41467-026-72038-y

**Published:** 2026-04-20

**Authors:** Xiaoyun Mo, Yongjie Huang, Haimin Chen, Junwen Huang, Kui Huang, Suwen Zhao, Xufei Wang, Kuibo Zhang, Yang Li

**Affiliations:** 1 Guangdong Provincial Engineering Research Center of Molecular Imaging, Guangdong-Hong Kong-Macao University Joint Laboratory of Interventional Medicine, the Fifth Affiliated Hospital, Sun Yat-sen University, Zhuhai, Guangdong, China; 2 Department of Pathology, The Affiliated Qingyuan Hospital (Qingyuan People’s Hospital), Guangzhou Medical University, Qingyuan, Guangdong, China; 3 Department of Spine Surgery, the Fifth Affiliated Hospital, Sun Yat-sen University, Zhuhai, Guangdong, China

**Keywords:** Rheumatoid arthritis, Drug delivery, Diagnostic markers, Three-dimensional imaging, Molecular imaging

## Abstract

The use of immunosuppressive therapies for rheumatoid arthritis (RA) is associated with substantial adverse effects. In addition, existing diagnostic reagents are limited in their ability to detect local joint damage. Here, we address both these needs using a collagen-hybridizing peptide (CHP) that selectively binds denatured collagen present in inflamed joints in RA models. Light-sheet scanning and immunohistology of the inflamed ankles in RA mice pre-injected with fluorescently-labeled-CHP revealed specific CHP accumulation within matrices of the synovial pannus and cartilage that express matrix metalloproteases and cathepsin K extensively. In vivo fluorescence probing with labeled CHP enabled dynamic monitoring of collagen breakdown throughout RA progression or regression with sensitivity beyond arthritis scoring. Moreover, bioconjugation of a therapeutic monoclonal antibody (mAb) against TNF with CHP enriched the mAb precisely within the pannus of inflamed joints in RA mice, resulting in improved efficacy relative to the native mAb and reduced systemic exposure. These findings highlight denatured collagen as a molecular matrix signature of RA lesions for diagnostic imaging and an effective target for RA drug delivery.

## Abstract (web summary)

The treatment and diagnosis of rheumatoid arthritis (RA) remain challenging. Here, the authors use a collagen-hybridizing peptide specific for damaged collagen, allowing both therapeutic targeting and imaging of inflamed joints in a mouse model of RA.

## Introduction

Seventy million people worldwide are suffering from multi-joint inflammation, chronic pain, and restricted mobility caused by rheumatoid arthritis (RA)^1,2^. RA is an autoimmune disease characterized by joint erosion, whose primary pathological manifestation includes synovitis and progressive destruction of the articular cartilage leading to joint deformity and loss of functions^3^. For diagnosis, conventional serological autoantibody markers, such as the anti-cyclic citrullinated peptide antibodies, provide little information on cartilage damage^4^. While elevated serum levels of cartilage oligomeric matrix protein (COMP) serve as a systemic indicator of cartilage metabolism and breakdown during RA progression^5^, it lacks the spatial resolution required to identify the specific anatomical sites of structural joint damage^6^. Radiographically, X-rays cannot visualize the cartilage and synovium^7^ and identifying subtle morphological changes in these soft tissues is also challenging with magnetic resonance imaging (MRI)^8^. For therapeutics, although the widely used glucocorticoids and anti-inflammatory monoclonal antibodies (mAbs) can effectively alleviate inflammation and cartilage degradation^9,10^, these treatments pose a significant risk of adverse effects^11,12^. For example, the use infliximab and adalimumab, two therapeutic mAbs against tumor necrosis factor (TNF) α is associated with general immunosuppression, increasing the risk of infection in patients^13,14^.

As such, developing molecular agents specifically targeting inflamed joint tissue is crucial in RA management for sensing local cartilage and synovium erosion and delivering immunosuppressive drugs to joint lesions with minimal systemic exposure^15^. Previous research on molecular imaging and drug delivery in RA has focused on inflammation, including the immune cells (macrophages overexpressing folate receptor β^16^, neovascularization (endothelial cells overexpressing α_v_β_3_ integrin)^17^, and metabolism (glucose uptake in macrophages and neutrophils)^18^. Moreover, fluorescence probes activated by ROS^19,20^, NO^21^, MMP13^22^, low pH^23^, and M1 macrophage^24^ have been showcased in RA microenvironments. Meanwhile, the synovium, cartilage, and subchondral bone constituting a joint are predominantly built by the collagenous extracellular matrix (ECM). As such, collagen- and other ECM-binding peptides were leveraged to localize and retain therapeutic antibodies within the collagenous RA lesions in recent years^25,26^. However, since collagen is so prevalent in the human body, generic collagen-targeting strategies may not be effective for RA imaging; they may even increase the risk of promoting RA drug uptake in non-target tissues.

The structural and functional deterioration of the joints in RA is caused by the breakdown of the collagenous matrices^27,28^, warranting deliberate attention in developing RA diagnostics and therapeutics^6^. During RA pathogenesis, the synovial fibroblasts and macrophages are extensively activated, expressing abundant matrix metalloproteases (MMPs) and cathepsin K, which degrade the collagens in the synovium, cartilage, and bones^29,30^. A range of circulating collagen-derived peptide fragments has been found in association with RA (e.g., C1M, C2M, and C3M)^31–33^. The collagen molecule is characterized by three intertwined polypeptide chains forming a unique triple-helix motif^34^, which can be denatured upon proteolytic degradation^35,36^. As such, we hypothesize that substantial amounts of degraded collagen chains may unfold and remain denatured within the RA joints, providing a matrix signature for monitoring and targeting joint damage in RA. We have previously shown that synthetic collagen hybridizing peptides (CHPs), featuring collagen-like proline-rich sequences [e.g., (GPO)_n_ or (GfO)_n_, O: hydroxyproline, f: (4S)-fluoroproline, *n* = 6–10], can specifically refold with the denatured collagen chains in situ and in vivo, presumably by reforming a hybrid triple-helix structure through interchain hydrogen bonds in a process analogous to how primers bind to melted DNA strands during a polymerase chain reaction^37^. Here, utilizing this collagen hybridization method, we visualize degraded and damaged collagen in mouse models of RA and evaluate it as an imaging biomarker of joint destruction. Furthermore, we modify an anti-TNF mAb with CHP conjugation and assess its targeted uptake and efficacy in the RA joints.

## Results

### Visualizing collagen breakdown in inflamed RA joints

**Fig. 1:**
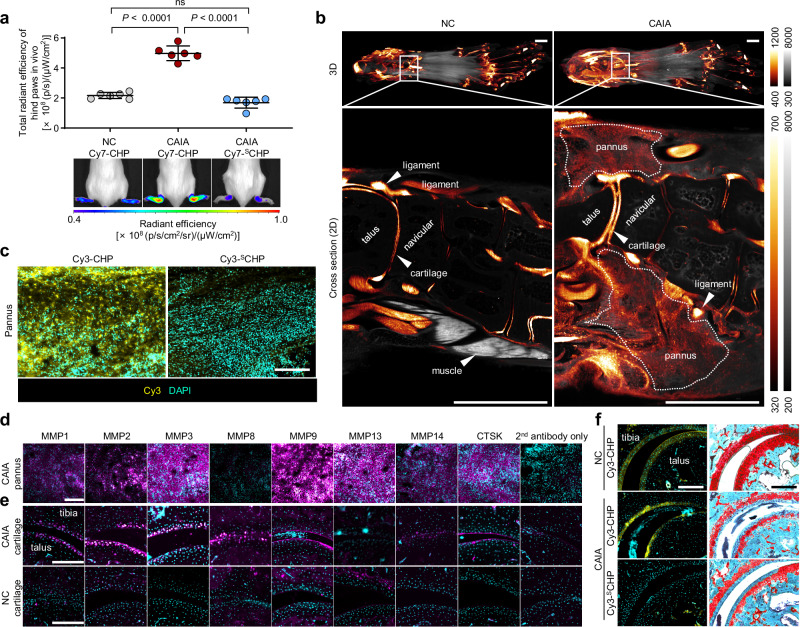
Tracing collagen breakdown in the inflamed RA joints in situ and in vivo via collagen hybridization. **a** Representative in vivo images and quantification of the fluorescence uptake from the hind paws of the normal control (NC) and CAIA mice 2 h post intravenous (i.v.) injection of 1 nmole of Cy7-CHP or its scrambled control peptide Cy7-^S^CHP (*n* = 6 hind paws from 3 mice). Data are shown as mean ± standard deviation. Statistical analysis: one-way ANOVA with Tukey’s test. **b** Representative light-sheet fluorescence microscopy images of the hind paws from the normal and CAIA mice collected and cleared 2 h post i.v. injection of Cy7-CHP. Coronal-section images from the three-dimensional scan of the inflamed ankle from the CAIA mouse showed prominent CHP binding to the denatured collagen in the expanding synovial pannus (dotted circles) and destructed cartilage. Pseudo-colors: glow, Cy7 fluorescence; gray, autofluorescence. **c** Representative fluorescence micrographs of the cryosections of the CAIA mice’s pannus tissue stained with Cy3-CHP or Cy3-^S^CHP. **d,** **e** Representative fluorescence micrographs of the cryosections of the CAIA mice’s ankle pannus tissue (**d**) and cartilage (**e**, compared with normal ankle cartilage) immunostained against various MMPs and cathepsin K (CTSK). The sections in the 2^nd^-antibody-only group were stained without any primary antibody. **f** Representative fluorescence micrographs of the cryosections of the ankle cartilage from normal or CAIA mice stained with Cy3-CHP or Cy3-^S^CHP showing pronounced CHP binding to areas of cartilage destruction (left); proteoglycan loss revealed by Safranin O staining of the neighboring sections (right). Representative images from three independent experiments with similar results (**b**–**f**). Scale bars: 1 mm (**b**) and 150 μm (**c**–**f**).

In this study, we used Balb/c mice with collagen antibody-induced arthritis (CAIA) as a mouse model (Supplementary Fig. 1a). This is a widely employed murine model of RA with high morbidity and reproducibility as well as pathological characteristics similar to human RA patients, including cartilage degradation and bone erosion (Supplementary Fig. 1b)^38^. Two hours post intravenous (i.v.) injection of Cy7-labeled CHP, drastically stronger in vivo near-infrared fluorescence signals were detected in the swollen ankle regions in the hind paws of the CAIA mice (6 days post-disease onset, hind paw arthritis score = 4) than those of the normal naïve mice (Fig. 1a, Supplementary Fig. 2).

In vivo imaging was performed at 2 h post-injection of Cy7-CHP to allow for the systemic clearance of non-specific circulating probes (Supplementary Fig. 2d), thereby ensuring that the remaining fluorescence specifically highlights collagen damage for accurate RA detection (Supplementary Fig. 2a). However, the control peptide Cy7-^S^CHP with a scrambled amino acid sequence and no collagen triple-helix folding capacity, displayed virtually no fluorescence signal within the inflamed paws (Fig. 1a). This result suggested that the CHP uptake in the inflamed ankles is contingent upon its triple-helical folding together with the probable denatured collagen within the joint tissues. Furthermore, fluorescence imaging of the hind paws dissected from the CHP-dosed mice post-skin-removal further confirmed the apparent CHP uptake in the inflamed ankles (Supplementary Fig. 3).

To track the exact anatomic location of CHP binding in the inflamed ankles, we used light-sheet fluorescence microscopy to scan the mice’s hind paws, which were dissected 2 h post i.v. injection of a Lys-modified Cy7-CHP [Cy7-Ahx-K-Ahx-(GfO)_9_] followed by tissue clearing using the PEGASOS method^39^ (Fig. 1b, Supplementary Fig. 4, see methods). The additional Lys residue provides a primary amine group that crosslinks with paraformaldehyde during fixation. This covalent stabilization enables the CHP probe, which otherwise associates with tissue non-covalently, to withstand rigorous washing and clearing protocols. Notably, the probe’s specificity and in vivo behavior remained unaltered following the Lys modification (Supplementary Fig. 5). Strong smoke-like Cy7 fluorescence signals in the inflamed ankle were located throughout the expanding synovial pannus, and the fluorescence from the articular cartilage between multiple bones was prominent (Fig. 1b, Supplementary Movies 1, 2). In contrast, in the normal paw, only the anatomical structures responsible for joint movements, such as articular surfaces, tendons, ligaments, and joint capsules, showed a low physiological level of CHP binding (Fig. 1b), likely due to collagen denaturation under mechanical wear during regular activities.

Histological analysis of inflamed ankle cryosections revealed robust Cy3-CHP fluorescence extensively distributed throughout the pannus, a hyperplastic and collagen-rich synovial tissue (Fig. 1c)^29^. CHP fluorescence signals preferentially colocalized with collagen IV immunostaining rather than collagen I (Supplementary Fig. 6). The absence of binding for the sequence-scrambled control (Cy3-^S^CHP) suggests that triple-helical hybridization is required for target engagement (Fig. 1c), a finding further supported by the competitive blocking experiments (Supplementary Fig. 7). Moreover, immunofluorescence analysis revealed prevalence of MMP1, 2, 3, 9, 13, 14 (esp. MMP1, 3, 9, and 13) and CTSK in the pannus cryosections (Fig. 1d) surrounded by the Cy3-CHP-marked denatured collagen (Supplementary Fig. 8), suggesting extensive matrix degradation. Strikingly, Cy3-CHP co-localized with the immunofluorescence from a commercial antibody targeting degraded collagen specifically in the pannus, but not the normal synovium (Supplementary Fig. 9). This finding directly supports the peptide’s specific binding to degraded collagen fragments in the pannus.

Moreover, immunofluorescence of the ankle cryosections showed marked upregulation of MMP1, 2, 3, 8, 9, and CTSK in the inflamed cartilage between the tibia and talus (Fig. 1e, Supplementary Fig. 10). At the exact location, Cy3-CHP (but not Cy3-^S^CHP) stained the RA ankle cartilage much more intensely compared with the normal controls, a result in line with the Safranin O staining of the neighboring sections showing surface fissures and proteoglycan loss (Fig. 1f). Overall, our histologic analysis confirmed our observation from in vivo and light-sheet fluorescence imaging; these results suggested that the collagens in the joint pannus and cartilage are extensively degraded by the various matrix proteinases during RA progression^30^, rendering them denatured and trackable by CHP hybridization (Fig. 1b, c, f).

### Dynamic monitoring of RA progression and regression

**Fig. 2:**
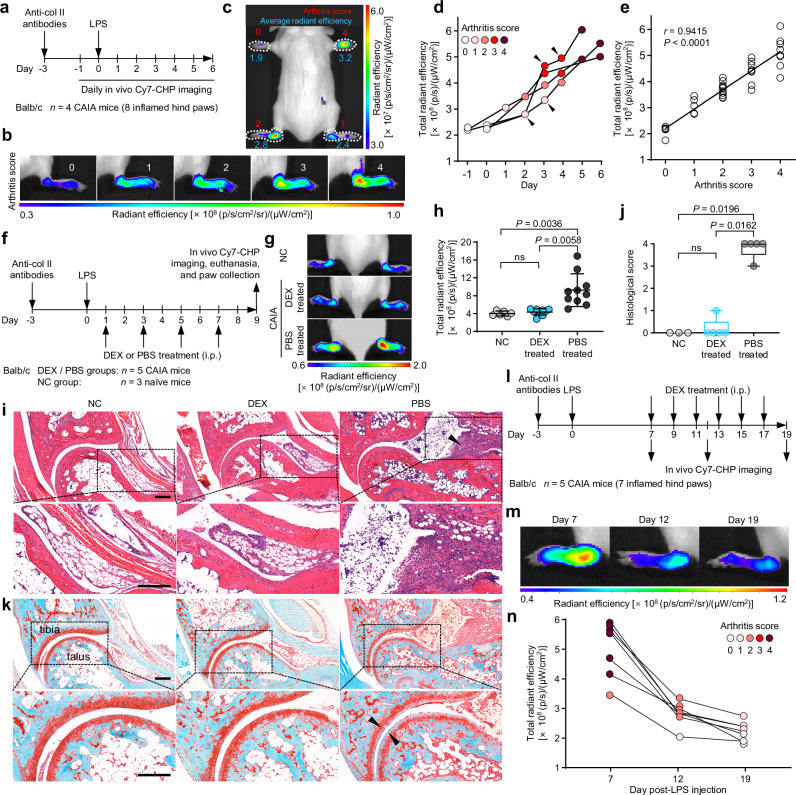
Live-monitoring collagen destruction in inflamed ankles. **a** Experimental design: daily in vivo near-infrared fluorescence imaging using i.v. injected Cy7-CHP in CAIA mice (*n* = 4). **b**, **c** Representative in vivo fluorescence images 2 h post-Cy7-CHP injection for ankles with arthritis scores 0–4 (**b**) and paw-specific fluorescence quantification in an individual mouse (**c**). **d** Quantified Cy7-CHP fluorescence in right hind paws over 8 days (*n* = 4 mice; see Supplementary Fig. 11 for left paws and Cy7-^S^CHP control). Arrowheads indicate increasing Cy7-CHP uptake over 24 h without arthritis score changes. **e** Correlation between Cy7-CHP uptake and arthritis scores (8 paws over 8 days). **f** Design of dexamethasone (DEX) treatment study (DEX / PBS groups: *n* = 5 CAIA mice, NC group: *n* = 3 naïve mice). DEX (1.5 mg/kg) or PBS was administered intraperitoneally every other day post-LPS injection. **g**, **h** Representative images (**g**) and quantification (**h**) of Cy7-CHP uptake in the hind paws at the endpoint. **i–k** Representative H&E (**i**) and Safranin O (**k**) staining, with histological scores (**j**) of right ankle joints. Arrowheads indicate synovial proliferation (**i**) or proteoglycan loss (**k**). Severity of synovial hyperplasia and bone resorption was scored 0–4 (**j**). **l** Design of the DEX treatment study monitored by Cy7-CHP in vivo imaging on days 7, 12, and 19 (7 inflamed paws from 5 mice). **m**, **n** Representative images (**m**) and quantification (**n**) of Cy7-CHP uptake in each inflamed hind paw during DEX treatment. Data points represent individual hind paws (**d**, **e**, **h**, **j**, **n**). Data are shown as mean ± standard deviation (**h**) or median with interquartile range (**j**). Statistical analysis: Two-sided Spearman correlation (**e**), Welch’s ANOVA with two-sided post hoc Dunnett’s T3 test (**h**), and Kruskal-Wallis test with post hoc Dunn’s test (**j**). Images are representative of three independent experiments with similar results (**i**, **k**). Scale bars: 200 μm (**i**, **k**).

Upon confirming that the 1 nmole i.v. dose of Cy7-CHP can be entirely cleared from both inflamed and normal ankle joints within 24 h (Supplementary Fig. 2), we proceeded to monitor collagen degradation in the hind paws of four CAIA mice throughout the disease onset and progression via daily Cy7-CHP injection and live imaging (Fig. 2a). Quantification of in vivo Cy7-CHP fluorescence in individual hind paws revealed a progressive increase in signal intensity over the six days following LPS injection, closely correlating with the rising arthritis scores (Fig. 2b–d, Supplementary Fig. 11a). In contrast, the signal from the scrambled control probe ^S^CHP remained consistently at baseline levels while the arthritis scores and associated inflammation increased over time (Supplementary Fig. 11b). This divergence demonstrates that the observed joint enrichment is a result of specific CHP hybridization to the progressive accumulation of denatured collagen, rather than a passive byproduct of hyperperfusion or vascular permeability. Moreover, slight increase of the Cy7-CHP signal was sensitively detected on sequential days even for paws with no change in the arthritis score (arrowheads, Fig. 2d). The varying CHP fluorescence levels in the four paws of a single mouse typically corresponded with each paw’s arthritis score (Fig. 2c). Statistically, the CHP fluorescence uptake in the hind paws measured from all images over the experimental duration was differentiable according to the arthritis score (Supplementary Fig. 12a); furthermore, strong positive correlations were observed between in vivo CHP fluorescence uptake and arthritis score (Spearman *r* = 0.9415, Fig. 2e) as well as between in vivo CHP uptake and ankle thickness (Pearson *r* = 0.9263, Supplementary Fig. 12b).

Next, we tested the in vivo detection of collagen degradation using Cy7-CHP in CAIA mice under treatment. From the second day following the LPS injection, every 48 h, we treated a group of CAIA mice with dexamethasone (DEX, intraperitoneal injection), a common corticosteroid for RA treatment with anti-inflammatory and immunosuppressive properties (Fig. 2f)^40^. In our experiment, the DEX treatment significantly lowered the arthritis score in CAIA mice compared to those treated with vehicle-only (i.e., PBS buffer) (Supplementary Fig. 13). On day 9 (the endpoint), we i.v. injected the mice with Cy7-CHP; the in vivo Cy7-CHP fluorescence from the hind paws of the DEX-treated mice was almost indistinguishable from the normal controls (Fig. 2g, h) and drastically lower than that of the PBS-treated group (hind paw arthritis scores = 2–4). Histologic analysis of each right hind paw of the DEX-treated mice showed normal joint morphology (with only one paw showing minor synovial hyperplasia, Fig. 2i, j) and undetectable cartilage damage (Fig. 2k), corroborating our in vivo CHP fluorescence measurements (Fig. 2h). Subsequently, we followed the therapeutic response using in vivo CHP imaging. This time, we started the DEX treatment on day 7 post-LPS induction when the paws reached maximal inflammation (Fig. 2l). We imaged the inflamed paws with Cy7-CHP on days 7, 12, and 19 (Fig. 2m); as expected, DEX treatment led to a progressive and concurrent decrease in both CHP fluorescence and arthritis scores across all monitored paws (Fig. 2m, n, Supplementary Fig. 14). Together, these findings demonstrated the feasibility and reliability of using collagen hybridization to image collagen degradation in vivo for dynamic, individual monitoring of RA progression and assessment of therapeutic efficacy.

### CHP-conjugation for localizing mAb to the inflamed paws

**Fig. 3:**
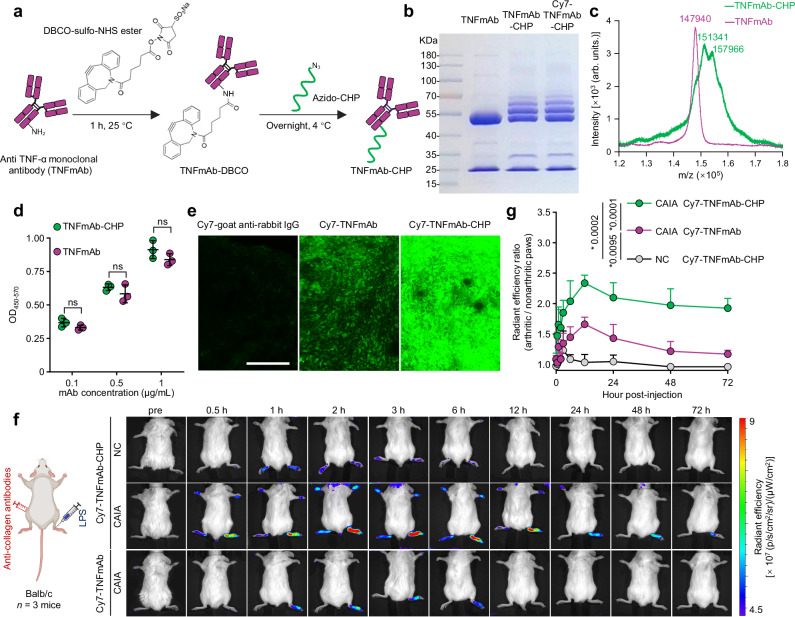
CHP-conjugated TNFmAb accumulates in the inflamed paw. **a** Scheme for chemical conjugation of CHP to TNFmAb via a DBCO-azide click reaction. **b** Coomassie blue-stained SDS-PAGE of TNFmAb-CHP, unmodified TNFmAb, and Cy7-labeled TNFmAb-CHP under reducing conditions. **c** MALDI-TOF MS of TNFmAb-CHP and unmodified TNFmAb. m/z: mass-to-charge ratio. **d** ELIZA assays comparing the reactivity of TNFmAb-CHP and TNFmAb against the recombinant mouse TNF-α protein (*n* = 3 independent experiments). **e** Representative fluorescence micrographs of cryosections of the synovial pannus from CAIA mice stained with Cy7-TNFmAb, Cy7-TNFmAb-CHP, or a Cy7-labeled goat-anti-rabbit IgG (2^nd^ antibody). **f** Arthritis was triggered selectively at the mice’s right hind paws by local injection of LPS at the right hind footpads. Representative serial in vivo fluorescence images of these single-paw CAIA or normal mice i.v. injected with Cy7-labeled TNFmAb-CHP or TNFmAb on the day following the LPS injection, showing long-lasting, selective accumulation of Cy7-TNFmAb-CHP in the arthritic right hind paw (*n* = 3 mice). **g** Changes in radiant efficiency ratio of the arthritic paws (right hind) to the non-arthritic paw (left hind) in the normal and CAIA mice (*n* = 3 mice). Data are shown as mean with standard deviation (**d**, **g**). Statistical analysis: Two-sided *t*-test (**d**) and one-way repeated measures ANOVA with Geisser-Greenhouse correction for sphericity and post hoc Tukey’s test (**g**). Representative images from three independent experiments with similar results (**b**, **e**). Scale bar: 250 μm (**e**).

Monoclonal antibodies against tumor necrosis factor α (TNFmAbs) are frontline biological RA therapeutics^41^. However, systemic exposure to the TNFmAb can lead to immune suppression and off-target effects^14^. To enhance the mAb uptake in the inflamed RA joint lesions, we conjugated a TNFmAb molecule (XT3.11 mAb, anti-mouse TNF-α rat IgG1) with the CHP, leveraging the peptide’s affinity to denatured collagen. We covalently modified the antibody with DBCO-sulfo-NHS ester, which was subsequently coupled via a copper-free click reaction to an azido-featuring CHP peptide (Fig. 3a, Supplementary Fig. 15a, see Methods). SDS-PAGE analysis showed an increase of approximately ~10–15 KDa in molecular weight for both the TNFmAb’s light and heavy chains after CHP conjugation (Fig. 3b); MALDI-TOF MS confirmed that each antibody molecule was modified with 3–4 CHP chains (MW: 3 KDa per chain) on average under our stoichiometric reaction condition (Fig. 3c, Supplementary Fig. 15b, c). ELIZA assays showed that the TNFmAb-CHP conjugate possessed full reactivity against TNF with no significant difference from that of the unmodified TNFmAb (Fig. 3d). We further labeled the TNFmAb and TNFmAb-CHP with Cy7 to visualize the collagen binding. The Cy7-TNFmAb-CHP conjugate exhibited a strong affinity for mouse skin tissues that we pre-heated in a boiling water bath to produce denatured collagen on purpose, whereas the Cy7-TNFmAb itself only bound minimally (Supplementary Fig. 16a); also, the unlabeled TNFmAb-CHP conjugate exhibited notable affinity to such pre-heated denatured mouse skin and tendon tissues compared to TNFmAb itself, detected immunochemically by a peroxidase-conjugated secondary antibody (Supplementary Fig. 16b). In cryosections, Cy7-TNFmAb exhibited preferential binding to the inflamed pannus of the CAIA mice; however, conjugation with CHP enhanced tissue accumulation multi-fold (Fig. 3e). Blocking experiments confirmed that this increased binding to the tissue is primarily driven by CHP-mediated hybridization with degraded collagen (Supplementary Fig. 17a). Furthermore, the Cy7-TNFmAb-CHP conjugate showed minimal binding to normal synovial tissues compared to the RA pannus (Supplementary Fig. 17b). Collectively, these in vitro and histological results demonstrate that the CHP conjugation preserves the intrinsic antigen-recognition capability of TNFmAb while endowing the conjugate a second affinity for the denatured collagen prevalent in the RA joint matrix.

To test the localization of the conjugate to the inflamed joint in vivo, we injected LPS selectively at the right hind footpad (Fig. 3f). This local LPS injection induced severe arthritis at the right hind paw compared to the left one (Supplementary Fig. 18a). On day 1 post-LPS injection, an equivalent dose of Cy7-labeled TNFmAb-CHP or TNFmAb was intravenously injected into this modified CAIA model and the normal controls, which were subjected to frequent near-infrared fluorescence imaging in 3 days (Fig. 3f). The Cy7-labeled TNFmAb-CHP rapidly accumulated in the inflamed right ankle from 0.5 to 3 h post-injection, whereas the Cy7-TNFmAb showed only weak signals (Fig. 3f, g, Supplementary Fig. 18); from 12 to 72 h post-injection the TNFmAb-CHP fluorescence declined slowly in the inflamed ankle compared to the TNFmAb (Fig. 3f, Supplementary Fig. 18c, d). The ratio of the Cy7 fluorescence level in the arthritic paw to the non-arthritic paw was significantly higher in mice injected with TNFmAb-CHP than with TNFmAb (Fig. 3g). Analysis of the area under the curve (AUC) for the fluorescence signal over 72 h revealed that the ratio of mAb uptake in arthritic versus non-arthritic paws was 3.1-fold higher for TNFmAb-CHP than for the parent TNFmAb (Supplementary Fig. 18b). These data demonstrated the CHP’s capacity to deliver its mAb conjugate to the arthritic ankle joint in vivo.

### CHP-conjugation enhances the efficacy of TNFmAb

**Fig. 4:**
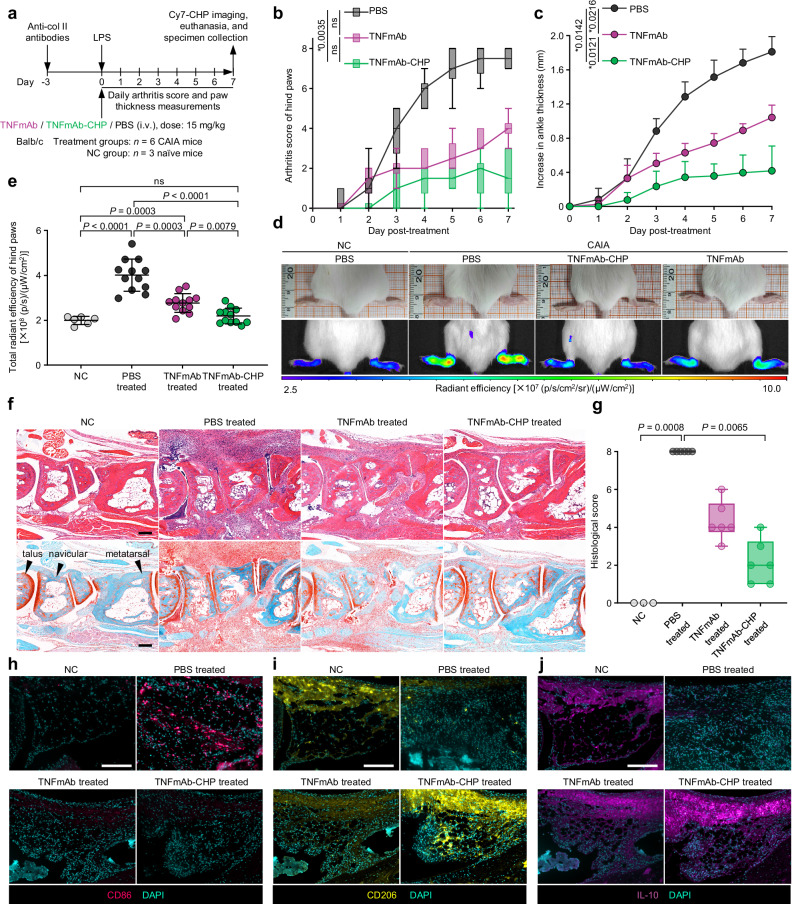
CHP-conjugation enhances the efficacy of TNFmAb in CAIA mice. **a** Experimental design: on the day of LPS injection (day 0), the CAIA mice received a single i.v. dose of TNFmAb-CHP / TNFmAb or vehicle (PBS) (Treatment groups: *n* = 6 CAIA mice, NC group: *n* = 3 naïve mice). **b** Combined arthritis scores for the two hind paws of the CAIA mice in the TNFmAb-CHP-, TNFmAb-, and PBS-treated groups. **c** Combined increases in ankle thickness compared to day 0 for the two hind paws of the CAIA mice in the TNFmAb-CHP-, TNFmAb-, and PBS-treated groups. **d** Representative photographs of the CAIA mice’s hind paws from each treatment group and their corresponding in vivo fluorescence images with Cy7-CHP compared to normal mice (acquired 2 h post i.v. Cy7-CHP injection on day 7). **e** Quantification of the Cy7-CHP fluorescence uptake in each hind paw in (**d**). **f** Representative H&E and Safranin O stains of the ankle joints collected on day 7 from the normal or CAIA mice in each treatment group. **g** Combined histological scores from each CAIA mouse’s two ankles in the three treatment groups compared to the normal controls (data points: individual mice). The severity of synovial hyperplasia and bone resorption was scored from 0 to 4 for each ankle as described in Methods. **h–j** Representative fluorescence micrographs of the ankle synovial tissue on day 7 from the normal or CAIA mice in each treatment group, immunostained for CD86 (**h**), CD206 (**i**), and IL-10 (**j**), respectively. Data are shown as median with interquartile range (**b,** **g**) or mean with standard deviation (**c**, **e**). Statistical analysis: Friedman test for repeated measures on ranks with post hoc Dunn’s test (**b**), one-way repeated measures ANOVA with Geisser-Greenhouse correction for sphericity with post hoc Tukey’s test (**c**), Welch’s ANOVA with two-sided post hoc Dunnett’s T3 test (**e**), and Kruskal-Wallis test with post hoc Dunn’s test (**g**). Representative images from three independent experiments with similar results (**h**–**j**). Scale bars: 200 μm (**f**) and 150 μm (**h**–**j**).

We next examined the efficacy of TNFmAb-CHP in the regular CAIA model with intraperitoneal injection of LPS (Fig. 4a). On the day of LPS injection, the CAIA mice received a single i.v. dose of TNFmAb or TNFmAb-CHP (dose: 15 mg/kg) or vehicle only (PBS). The hind paw’s arthritis score and the increase in ankle thickness for the TNFmAb-CHP-treated mice were consistently half of those observed in the TNFmAb group throughout the testing period (Fig. 4b-c). The redness and swelling were visibly attenuated for the TNFmAb-CHP-treated ankles compared to the TNFmAb-treated ones (Fig. 4b, Supplementary Fig. 19). The outcomes in the front paws were also consistent with the hind paws (Supplementary Figs. 20, 21), and the total increase in joint thickness for the four paws of TNFmAb-CHP-treated CAIA mice was also consistently lower than in the TNFmAb group (Supplementary Fig. 22). On day 7 post-mAb-treatment, in vivo, near-infrared fluorescence imaging using Cy7-CHP revealed a marked reduction in collagen degradation and denaturation in the hind paws treated with TNFmAb-CHP than those treated with TNFmAb (Fig. 4d, e, Supplementary Fig. 23); a similar trend was also noted in the front paws (Supplementary Fig. 23). Endpoint histological examination and scoring of the ankle joints revealed that synovial hyperplasia, inflammatory cell infiltration, and articular cartilage erosion were effectively attenuated by TNFmAb-CHP compared to TNFmAb (Fig. 4f, g). Furthermore, immunostaining revealed an increase in anti-inflammatory IL-10 and CD206⁺ cells (possibly M2 macrophages)^2,42^ within the pannus of the TNFmAb-CHP group compared to the TNFmAb group, as well as a decline in CD86^+^ cells (possibly M1 macrophages)^43^ in the TNFmAb-CHP-treated pannus compared to the PBS-treated group (Fig. 4h–j). These data suggest that the CHP conjugation with TNFmAb provides superior anti-inflammatory efficacy in the CAIA model relative to the unmodified mAb.

We further challenged TNFmAb-CHP by initiating the treatment after RA progression in the CAIA mice had reached an average score of 1 (characterized by digit swelling) or 2 (characterized by wrist/ankle and digit swelling) per paw. Assessments of the arthritis score and paw thickness over 7 days post-treatment demonstrated that TNFmAb-CHP remained significantly more effective than TNFmAb in mitigating RA progression in these two scenarios (Supplementary Figs. 24, 25).

### Anatomical locating CHP-delivered TNFmAb in the arthritic paws

**Fig. 5:**
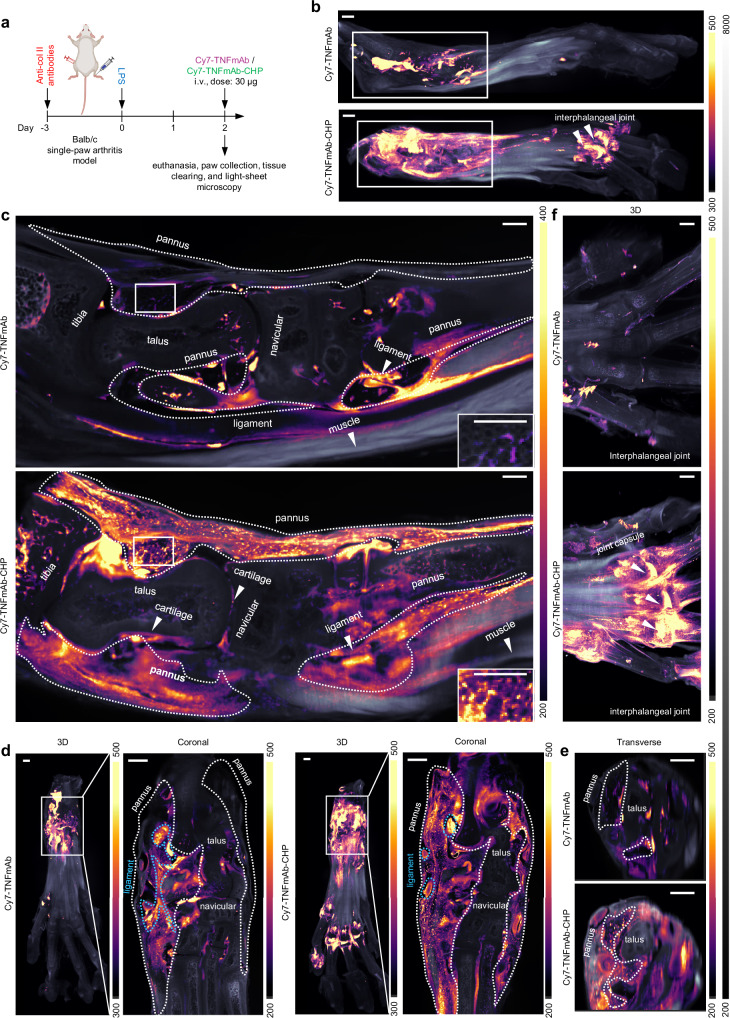
CHP conjugation directly delivers TNFmAb to the pannus of the arthritic paws. **a** Experimental design. Arthritis was selectively induced in the right hind paws of the CAIA mice by local LPS injection on day 0. The inflamed right hind paws were collected for tissue clearing 2 h post i.v. injection of Cy7-TNFmAb-CHP or Cy7-TNFmAb on day 2. **b**–**f** Representative light-sheet fluorescence microscopy images showing the distinct anatomical locations of Cy7-TNFmAb and Cy7-TNFmAb-CHP distribution within the arthritic hind paws (arthritis score: 2). 3D holistic view (**b**); 2D sagittal view (Bottom right thumbnails: zoomed-in images for the boxed areas) (**c**); 3D and 2D coronal views (**d**); 2D transverse view (**e**); 3D coronal view of the toe digits (**f**). Pseudo-colors: mpl-inferno, Cy7 fluorescence; gray, autofluorescence. Representative images from three independent experiments with similar results (**b**–**f**). Scale bars: 250 μm (**c**), 500 μm (**b**, **d**–**f**).

We traced the distribution of the CHP-conjugated TNFmAb-CHP within the anatomical locations of the inflamed hind paws of the CAIA mice using light-sheet microscopy. On day 2 post-LPS-induction of arthritis in the right hind footpad (Fig. 5a, arthritis score: 2), both mice were intravenously injected with an equal dose of Cy7-labeled TNFmAb-CHP or TNFmAb. Two hours post-injection, their hind paws were collected and cleared. The 2 h time point was selected to ensure a robust fluorescence signal for the Cy7-TNFmAb-CHP conjugate while maintaining detectable signal levels in the more rapidly cleared Cy7-TNFmAb control group in the RA joint (Fig. 3f), thereby enabling a direct and meaningful spatial comparison. To eliminate non-specific signals from the circulation, all animals were subjected to extensive systemic perfusion with PBS prior to tissue collection, ensuring that the detected fluorescence specifically represents tissue-localized mAbs. Light-sheet fluorescence imaging revealed that Cy7-TNFmAb was primarily present in the joint cavity, with signals on the joint cavity linings and the ligament surfaces (Fig. 5b, c). In striking contrast, Cy7-TNFmAb-CHP accumulated prominently within the expanding pannus. Notable from views in all three anatomical planes, the entire synovial pannus surrounding the tibial, navicular, and talus bones was enriched with Cy7-TNFmAb-CHP, but not Cy7-TNFmAb (Fig. 5c–e). The only fluorescence that could be seen in the magnified fields of view of the pannus was from the Cy7-TNFmAb-CHP bound to the tissue (granular signals, Fig. 5c inset). Critically, this difference in signal intensity was not attributable to variations in disease severity. Histopathological analysis of the same paw specimens post light-sheet microscopy confirmed equivalent levels of RA-associated pathology across both groups (Supplementary Fig. 26). Furthermore, in stark contrast to Cy7-TNFmAb, Cy7-TNFmAb-CHP demonstrated substantial uptake within the inflamed interphalangeal joints (Fig. 5f, b, arrowheads). Meanwhile, we noted that the spatial distribution of Cy7-TNFmAb-CHP administered in mice on day 1 post-LPS-induction (arthritis score: 1) was highly similar to the above results (Supplementary Fig. 27), whereas fluorescence was markedly less pronounced in the synovial tissues of the contralateral non-arthritic left hind paws (internal control, Supplementary Fig. 28). These findings demonstrate that CHP selectively anchors TNFmAb to the destructed collagen within the lesion—the synovial pannus—providing strong support for the superior efficacy of TNFmAb-CHP over TNFmAb as seen in Fig. 4, Supplementary Figs. 24, 25.

Furthermore, we demonstrated that the CHP peptide selectively binds to the inflamed synovial pannus of a collagen-induced arthritis (CIA) mouse model, as well as to the arthritic shoulder joint cartilage from an RA patient (Supplementary Figs. 29, 30). Crucially, this collagen hybridization-mediated binding also enabled the TNFmAb-CHP conjugate to adhere to these pathological tissues (Supplementary Figs. 29, 30). These data implied that our targeting strategy would be effective across different RA models and potentially in human RA.

### Pharmacokinetics and biodistribution of Cy7-TNFmAb-CHP in CAIA mice

**Fig. 6:**
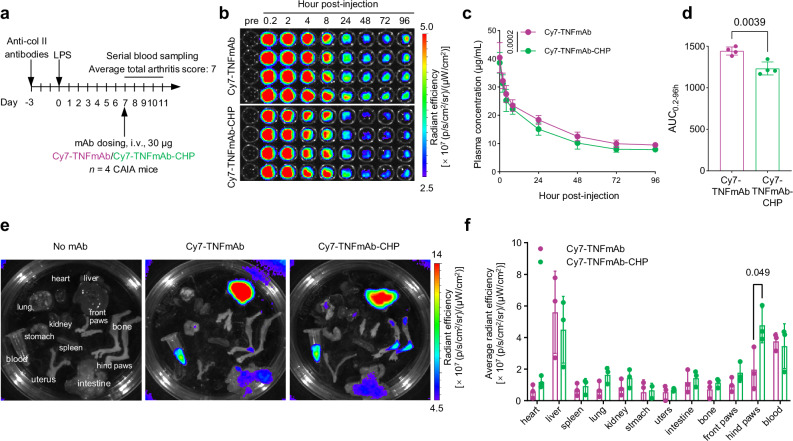
Pharmacokinetics and biodistribution of Cy7-TNFmAb and Cy7-TNFmAb-CHP in CAIA mice. **a** Experimental design: On day 7 post-LPS injection (average total arthritis score of four paws per mouse: 7.5, *n* = 4 CAIA mice), CAIA mice received a single i.v. injection of Cy7-TNFmAb or Cy7-TNFmAb-CHP, followed by serial blood collection at designated time points. **b** Fluorescence intensity of the Cy7-TNFmAb or Cy7-TNFmAb-CHP containing plasma (50 μL/well) at different time points. **c** Quantification of the Cy7-TNFmAb and Cy7-TNFmAb-CHP concentrations in the plasma of the CAIA mice in (**b**). **d** Areas under the curve (AUC) of the plasma concentration-time curves for Cy7-TNFmAb and Cy7-TNFmAb-CHP from 0.2 to 96 h post-injection. **e** Representative fluorescence images of the major organs, paws, and blood of CAIA mice harvested with no mAb injection (No mAb, *n* = 1 mouse) or 24 h post i.v. injection of 30 μg of Cy7-TNFmAb or Cy7-TNFmAb-CHP (average total arthritis score per mouse: 7, *n* = 3 mice). **f** Average radiant efficiency of each organ quantified from the fluorescence images in (**e**). Data are shown as mean ± standard deviation (**c**, **d**, **f**). Data points: individual mice (**d**, **f**). Statistical analysis: Two-sided paired *t*-test (**c**), two-sided Welch’s unpaired *t*-test (**d**), and two-sided unpaired *t*-test (**f**).

Finally, we characterized the longitudinal blood concentrations of Cy7-TNFmAb-CHP and Cy7-TNFmAb via Cy7 fluorescence measurements of serial blood samples collected from CAIA mice 0.2–96 h post i.v. injection (Fig. 6a, b). Both probes exhibited a similar gradual decline, approaching a plateau by 96 h (Fig. 6b, c). At equivalent dosing, the area under the concentration-time curve for Cy7-TNFmAb-CHP was approximately 85.4% of that observed for TNFmAb within 96 h post-injection (Fig. 6d). Interestingly, the pharmacokinetic profiles of the two constructs were nearly indistinguishable in healthy control mice (Supplementary Fig. 31). This suggests that the modest reduction in serum concentration observed for Cy7-TNFmAb-CHP in CAIA mice (Fig. 6c, d) may be partially attributable to its enhanced retention within inflamed RA joints.

We further assessed the biodistribution of Cy7-TNFmAb-CHP and Cy7-TNFmAb in major organs of CAIA mice 24 h post-injection via fluorescence imaging (Fig. 6e). Aside from the inflamed paws, the Cy7 fluorescence intensities from Cy7-TNFmAb-CHP and Cy7-TNFmAb were comparable across all harvested organs and tissue (Fig. 6f). Similar biodistribution profiles were observed at 2 h post-injection (Supplementary Fig. 32).

To investigate the in vivo toxicity of the TNFmAb-CHP conjugate, we also analyzed the lungs, livers, spleens, kidneys, and hearts of all the mice at the endpoint of the efficacy study (Fig. 4a). Histology of the organs from the TNFmAb-CHP-treated mice showed intact cellular and tissue architecture without apparent inflammation, hemorrhage, or necrosis, comparable to that of the TNFmAb and normal groups (Supplementary Fig. 33). Collectively, these data suggest that CHP conjugation enhances the therapeutic index of TNFmAb by facilitating targeted joint retention without significantly altering systemic clearance or inducing off-target organ toxicity.

## Discussion

Our study pinpoints the denatured collagen chains in the inflamed joints as a matrix marker of rheumatoid arthritis. Current molecular imaging strategies applied to RA primarily target the immune (e.g., macrophages, T/B cells, and neutrophils) and stromal cells (e.g., vascular endothelial cells and fibroblasts) and focus on pathways implicated in metabolism, proteolysis, and cell adhesion^44^. These studies have been instrumental in elucidating the mechanisms of RA progression and identifying therapeutic targets. Yet they fail to indicate the destruction to the ECM, the primary structural component of the joints. RA is defined by joint damage and degeneration, where synovial tissue undergoes drastic proliferation, neovascularization, and expansion, forming the pannus tissue with recruited inflammatory cells. This process is marked by high expression of matrix proteases (Fig. 1d, e)^3,30^. The altered matrix turnover has long been recognized as a pathological hallmark of RA^45^ but has rarely been exploited for its molecular imaging. We envision that a substantial portion of collagen molecules in the affected joints can be proteolyzed while the remaining collagen chains adopt a denatured conformation within the matrix. In this study, we validated this hypothesis histologically and in vivo via collagen hybridization (Fig. 1). We further located substantial collagen denaturation in regions of upregulated MMP (mainly MMP 1, 2, 3, 9, 13) and cathepsin K expression within the pannus and cartilage of the RA-affected ankles (Fig. 1d, e, Supplementary Figs. 8–10) and specified major denatured collagen types (Supplementary Fig. 6). Our data also exhibited a positive correlation between the level of collagen denaturation (estimated by in vivo fluorescence CHP update) and the arthritis score of the joints (Fig. 2b–e, Supplementary Fig. 12). Existing protease-targeted molecular imaging studies for RA (e.g., monoamine oxidase B^46^ and MMP13^47^) are often indirect and rely on measuring the enzyme activity on the probes. Here, based on the collagens’ conformational change rather than the upregulation of protease expression or activity, the CHP-collagen hybridization enables direct engagement and visualization of the products of matrix proteolysis, offering a straightforward method for detecting and monitoring RA.

Radiology is the most common way to monitor joint damage in RA in the clinic. X-ray imaging can visualize some anatomical changes to the RA joints, such as bone erosion, joint space narrowing, and periarticular bone loss^7^; ultrasound examinations can inform bone degradation, synovitis, and tendon pathology^48^. However, these modalities lack precise and quantitative criteria, so the disease assessment often depends on the skills and experience of the examiners. Meanwhile, in research laboratories, since serological analysis cannot examine RA pathogenesis in the context of synovial anatomy and histology investigation requires animal sacrificing and joint collection, subjective visual arthritis scoring is the most common method for joint impairment assessment in RA animal studies. As such, we tested and demonstrated in vivo dynamic monitoring of the changes to the joints via collagen hybridization during RA progression and regression, respectively (Fig. 2d, pathological changes; Fig. 2n, therapeutic changes under treatment). These findings offer a unique preclinical molecular imaging approach to continually monitor RA severity and evaluate therapeutic efficacy in individual animals, which can be more objective and quantitative than arthritis scoring. For future translational applications, we will develop CHP-based imaging agents compatible with clinical modalities extending beyond the near-infrared fluorescence used in this work (e.g., ultrasound and photoacoustic imaging).

Because regular tissue-section staining only provides limited two-dimensional information and cannot fully recapitulate the in vivo behavior of the imaging probes, we applied optical sectioning enabled by light-sheet microscopy to resolve the in vivo CHP binding in three dimensions, delineating the extent and range of collagen breakdown across the anatomy of the inflamed joints in situ (Fig. 1b). We observed pronounced collagen denaturation in the cartilage and the proliferative pannus enveloping the inflamed ankles (Fig. 1b), indicating drastic collagen catabolism during synovial pannus expansion and cartilage erosion in RA^49^. This methodology could be applied to various potential tractable targets in inflammatory joint diseases.

Our study demonstrates denatured collagen as a target for drug delivery towards the articular lesions in RA. Biologics such as anti-TNF mAbs are clinically validated RA therapeutics^41^. However, only a portion of the mAb dose could reach the target avascular joint tissues following systemic administration, with the majority distributed throughout normal tissues via circulation^26,50,51^, leading to off-target effects such as opportunistic infections and heart failure^13,52^. Currently, therapeutic delivery strategies targeting the ECM components are investigated for improving RA treatment outcomes^6,15,25,53^, including the use of proteoglycan-targeting cysteine-dense peptides to deliver corticosteroids to cartilage^54^, as well as conjugation of ECM-binding peptides to anti-TNF mAbs to improve mAb retention within the collagenous joint lesions^25,26^. These studies showcase that directly targeting ECM molecules can help overcome the rapid drug clearance from inflamed joints during intra-articular drug administration for monoarthritis^6,25,55^. However, systemic delivery remains a challenge for such strategies, since collagen is ubiquitous in the body, and generic collagen affinity could also result in drug retention in normal and fibrotic tissues^56–58^. Therefore, approaches to link anti-inflammatory agents to binding proteins that recognize ECM molecules selectively upregulated at disease sites can be beneficial^6,59,60^. For diseases featured by ECM destruction rather than synthesis, such as RA, we validated the delivery of therapeutic mAbs selectively to the destruction sites using collagen hybridization (Fig. 3). Despite achieving extended joint retention in healthy mice, previous CHP-conjugated infliximab fragments did not yield measurable therapeutic benefits in RA model mice^61^. In contrast, our findings demonstrated that conjugating CHP chains (with a molecular weight of only 2% of an IgG) to a full anti-TNF mAb significantly enriches the mAb in the RA-affected joints (Fig. 3), achieving more effective suppression of inflammation and collagen breakdown compared to the unmodified mAb (Fig. 4).

More importantly, the CHP-conjugate does not simply enrich TNFmAb in the joints but endows the mAb with spatially defined delivery targeting the inflamed joint microenvironment. Unlike unmodified TNFmAb, which relies mainly on passive uptake via hypervascularization and increased vascular permeability in the inflamed RA joints (Fig. 3f–g) without tissue selectivity (Fig. 5, Supplementary Fig. 27), the CHP-conjugation enables precise locating of TNFmAb to the synovial pannus—the front line of RA pathology—across all joints in the inflamed paws, including the interphalangeal joints (Fig. 5, Supplementary Fig. 27). We propose that such locational specificity, a feature not offered by many matrix-targeting molecules, constitutes the main mechanism underlying the superior efficacy of TNFmAb-CHP^6^. Furthermore, our study demonstrates the unique utility of light-sheet microscopy in visualizing the in vivo spatial distribution of therapeutics across tissue architectures^62^. While CHP conjugation led to a modest reduction in systemic exposure (Fig. 6d), it facilitated a preferential accumulation of TNFmAb within the inflamed ECM of RA joints (Fig. 6f). This subtle shift in biodistribution suggests a promising strategy for improving the therapeutic index and reducing the potential for systemic side effects (Fig. 6). We also provide preliminary evidence that the collagen-hybridizing targeting strategy can be applied to different RA animal models and possesses potential for clinical translation (Supplementary Figs. 29, 30).

The high specificity and efficacy of mAbs make them widely utilized in treating many cancers and autoimmune diseases^63^. The molecular targets of mAbs (e.g., PD-1/PD-L1, TNF, VEGF, HER2, FGF, TGF-β) are generally membrane receptors located on the cell surface or cytokines released into the ECM^64,65^. Therefore, delivering mAbs to the ECM should not affect the functional engagement with their therapeutic targets outside the cells. Moreover, providing these therapeutic mAbs and immunomodulatory agents the capacity to home in on the pathological ECM extends their retention at the disease site and limits the off-target effects on normal tissues, thus widening their therapeutic windows. Numerous malignancies, autoimmune disorders, and chronic inflammation typically involve ECM destruction or fibrotic remodeling^36,66^, creating altered tissue microenvironments populated with immunocytes and fibroblasts, whose functions are generally antagonized by mAb drugs. Using RA as a case study, our findings prove the concept of therapeutic mAb delivery to such lesion microenvironments by targeting the pathological changes to ECM, implicating a class of future biologics that can deliver localized, site-specific treatments across a range of critical disorders.

## Methods

### Mouse studies

All procedures for mouse maintenance and experiments were strictly followed the policy of the Experimental Animal Ethics Committee of the Fifth Affiliated Hospital of Sun Yat-sen University (protocol number: 00335). All mouse studies are summarized in Supplementary Table 1.

#### CAIA models

Female Balb/c mice (7 to 8 weeks old) were purchased from Zhiyuan (Guangdong) Biomedical Technology Co., Ltd. (stock number: XW20240620-01) and co-housed in a specific-pathogen-free (SPF) grade facility with a 12-h cycle of light and darkness at a controlled temperature of 20 to 24 °C and humidity of 40 to 60%, with free access to food and water. Consistent with established protocols for the CAIA model, female mice were used in this study to reflect the higher clinical prevalence of RA in women and to minimize the confounding effects of male aggression on arthritis scoring. Arthritis was induced by intraperitoneal injection of a cocktail of anti-collagen antibodies (1.5 mg per mouse; Chondrex, 53100) 3 days before an intraperitoneal injection of lipopolysaccharide (LPS, 25 μg per mouse; Chondrex, 53100, on day 0). Changes in paw thickness were monitored daily with a caliper. Paws were scored individually according to the manufacturer’s protocol (Chondrex): 0 = normal; 1 = mild, but definite redness and swelling of the ankle or wrist, or apparent redness and swelling limited to individual digits, regardless of the number of affected digits; 2 = moderate redness and swelling of the ankle or wrist; 3 = severe redness and swelling of the entire paw, including digits; 4 = maximally inflamed limb involving multiple joints. The scores for both hind paws were often summed for each mouse (from 0 to 8 in total).

For Figs. 3f, 5 and Supplementary Figs. 27, 28, the single-paw arthritis model was induced with LPS (12.5 μg per mouse) subcutaneously injected locally into the right hind paw on day 0, 3 days post intraperitoneal injection of the anti-collagen antibody cocktail^25^. At the end of the experimental procedures, mice were euthanized by cervical dislocation while under deep anesthesia induced by inhalation of 1–2% isoflurane.

#### CIA model

Inflamed hind paws from collagen-induced arthritis (CIA) mice (DBA/1 mice, male, 14 weeks old) were kindly provided by Dr. Yi Wang from Zhejiang University, from which pannus tissue was carefully dissected and cryosectioned into 5 μm sections (Supplementary Fig. 29).

#### Dexamethasone (DEX) treatment studies

The therapeutic dose of DEX (Macklin, D829854) was approximately 1.5 mg/kg. For Fig. 2f–k, female Balb/c mice (7 to 8 weeks old, body weight: 20 ± 1 g) were randomly divided into 3 groups: the PBS-treated CAIA group, the DEX-treated CAIA group, and the PBS-treated normal control (NC) group (Supplementary Table. 1). DEX (0.2 mg/mL, in 150 μL PBS) or PBS (150 μL) was intraperitoneally administered on days 1, 3, 5, and 7 post-LPS injection. For Fig. 2l–n, five CAIA mice were injected intraperitoneally with DEX (0.2 mg/mL, in 150 μL PBS) every other day from day 7 (when paw arthritis peaked) to 17 post-LPS injection.

#### Micro-computed tomography (μ-CT)

For micro-CT scanning, the collected hind paws were fixed in 4% paraformaldehyde (PFA, Biosharp, BL539A). The specimens were fitted in a cylindrical sample holder and scanned by nanoScan PET/CT 425 82S (Mediso) (Supplementary Fig. 1b). The voltage of the scanner was 80 kV, and the resolution was 18 nm/pixel. For visualization, the segmented data were imported and reconstructed into a 3-dimensional image using RadiAnt DICOM Viewer (64-bit).

### Peptide synthesis and labeling

As described in our previous reports, peptides were synthesized on a Rink Amide-AM resin (HECHENG, loading: 0.37 mmol/g) with standard Fmoc-chemistry solid-phase synthesis using a PurePep Chorus peptide synthesizer (Gyros Protein Technologies). The peptides were fluorescently labeled with the N-hydroxysuccinimide (NHS) ester of either sulfo-Cyanine 3 (Cy3, Lumiprobe, 41320) or sulfo-Cyanine 7 (Cy7, Lumiprobe, 25320) on resin before cleavage and purified by reverse phase high-performance liquid chromatography (RP-HPLC) on a semi-preparative column (Agilent ZORBAX StableBond 300 C18) using a mixture of H_2_O (0.1% TFA, v/v) and acetonitrile (0.1% TFA, v/v) as the mobile phase (4 mL/min). The purified peptides were subsequently lyophilized and characterized by matrix-assisted laser desorption/ionization time-of-flight (MALDI-TOF) on a Shimadzu MALDI-8020 instrument (Supplementary Table. 2).

In this study, Cy3-CHP with a sequence of (GPO)_9_ [O: (2S,4 R)-4-hydroxyproline] was used in in vitro tissue staining experiments (Fig. 1c, f, Supplementary Figs. 6–10) following a preheating step. Because (GPO)_9_ has a strong capacity to self-assemble into a peptide triple-helix, Cy3-CHP needs to be heated at 80 °C to dissociate the peptide homotrimer into single strands before staining. The non-collagen-hybridizing control peptide Cy3-^S^CHP has a scrambled amino acid sequence: Cy3-Ahx-PGOGPGPOPOGOGOPPGOOPGGOOPPG (e.g., Fig. 1c, f). Because the preheating step complicates the tail vein injection process, in all in vivo experiments in this study, we utilized Cy7-CHP with the sequence of (GfO)_9_ [f: (2S,4S)-4-fluoro-proline], which cannot self-trimerize at body temperature but still binds to denatured collagen^67^. The non-collagen-hybridizing control peptide Cy7-^S^CHP has a scrambled sequence of Cy7-Ahx-OfGGOfGfGfOfOGOfGOOfGGOOffG.

To improve the preservation of the CHP fluorescence signal in the paws for light-sheet fluorescence microscopy (Fig. 1b, Supplementary Movies), a Lys-featuring CHP sequence of Cy7-Ahx-K-Ahx-(GfO)_9_ was used to allow covalent crosslinking between the fluorescent probe’s Lys amine sidechain and the joint tissues during the paraformaldehyde fixation step after the paw collection. A peptide precursor NH_2_-Ahx-K(Dde)-Ahx-(GfO)_9_ was first synthesized with Fmoc-Lys(Dde)-OH (Aladdin F181651) on resin, cleaved, and purified by HPLC (linear gradient: 5−50% acetonitrile in 25 min). The lyophilized peptide (2.5 μmol), sulfo-Cyanine 7 NHS ester (1 μmol), and DIEA (20 μmol) were mixed in anhydrous DMSO (150 μL), and the reaction mixture was stirred at 25 °C for 24 h in the dark to produce Cy7-Ahx-K(Dde)-Ahx-(GfO)_9_. Subsequently, the Dde protective group on Lys was removed with 5% (v/v) hydrazine in water before the final HPLC purification (linear gradient: 10−60% acetonitrile in 25 min) and MALDI-TOF characterization (Supplementary Table 2).

For bioconjugation with the TNF monoclonal antibody, we synthesized and purified an azide-featuring CHP peptide NH_2_-K(N_3_)-Ahx-(GfO)_9_ using Fmoc-K(N_3_)-OH (Bidepharm, BD317054) on resin, and prepared and characterized its Cy7 conjugate [Cy7-K(N_3_)-Ahx-(GfO)_9_] (Fig. 3, Supplementary Fig. 15) by solution-phase labeling as described above (Supplementary Table 2).

#### Gelatin binding assays

For Supplementary Fig. 5a, wells of a 96-well plate were coated with approximately 6 μL of 70 °C hot gelatin (Sigma, G2500) solution in PBS (10% w/v) before incubation at 4 °C for 10 min to allow gelation. PBS solution (100 μL) containing 10 μM of Cy7-CHP or Lys-modified-Cy7-CHP [Cy7-Ahx-K-Ahx-(GfO)_9_] or Cy7-^S^CHP was heated at 45 °C for 10 min before being immediately added to each well and allowed to bind at 4 °C overnight (*n* = 4). The plate was washed three times with 200 μL of PBS at room temperature. The fluorescence intensity of each well was detected with a Biotek Synergy HTX plate reader (ex/em: 750/800 nm, gain: 1000).

### Antibody labeling and bioconjugation

#### Cy7-TNFmAb

Sulfo-Cyanine 7 (Cy7) NHS ester (1.0 mg, 1.18 μmol) was dissolved in 1 mL dimethyl sulfoxide (DMSO). TNF-α monoclonal antibody [7.94 mg/mL, ~ 0.01 μmol (assuming MW of 150 KDa) in 200 μL PBS, clone: XT3.11, Bio X Cell, BE0058] was diluted in 1800 μL of PBS. Five molar equivalents of Cy7 (45 μL in DMSO) were added to and mixed with the TNF mAb solution (2 mL in PBS) for 2 h at 25 °C in the dark.

After the labeling reaction, excess Cy7 was removed by centrifugation tube (MWCO 100 KDa, Millipore, UFC810096) through repeated centrifugation (Eppendorf, model 5427 R) in PBS at 358 g [4000 revolutions-per-minute (rpm)] at 4 °C until no Cy7 absorbance was detected in the filtrate at 750 nm measured with a UV-vis spectrophotometer (Implen, NP80touch). The mAb concentrations of the Cy7-TNFmAb solutions were determined using the BCA method.

#### TNFmAb-CHP conjugation

The conjugation steps are schematically described in Supplementary Fig. 15. First, 1 mg DBCO-sulfo-NHS ester (1.87 μmol, Click Chemistry Tools, A124-25) was dissolved in 187 μL of PBS to prepare a stock solution (10 mM), from which 5 μL solution (5 molar equivalents, 0.05 μmol) was added to and mixed with 200 μL of the TNFmAb solution (7.94 mg/mL, 0.01 μmol in PBS) for 1 h at 25 °C. Unreacted DBCO-sulfo-NHS ester was removed using an ultrafiltration tube (MWCO 100 KDa) through repeated centrifugation in PBS at 358 g (4000 rpm) at 4 °C. This process was repeated until no DBCO-specific absorbance was detected in the filtrate at 310 nm. Ten molar equivalents of NH_2_-K(N_3_)-Ahx-(GfO)_9_ peptide (0.1 μmol) were added to the purified TNFmAb-DBCO solution in PBS and mixed at 4 °C overnight. The excess peptide was removed by a centrifugation tube (MWCO 100 KDa) in PBS at 358 g (4000 rpm) until no absorbance peak was detected in the filtrate at 214 nm. In this step, we found that the free CHP peptide could trimerize with the TNFmAb-CHP product in solution at 4 °C, complicating its removal by ultrafiltration. Therefore, we performed the centrifugation at 37 °C to accelerate the removal of the excess CHP peptide. Finally, the TNFmAb-CHP was collected from the retentate for RA treatment. The Cy7-TNFmAb-CHP was produced by conjugating TNFmAb with Cy7-K(N_3_)-Ahx-(GfO)_9_ following the same method (Fig. 3a). The protein concentrations of the antibody conjugates were determined using the BCA assay before any in vitro or in vivo testing. About 50-65% of the TNFmAb protein can typically be recovered after the final conjugation step. The resulting products were characterized using a Bruker ultrafleXtreme MALDI-TOF/TOF mass spectrometry instrument (Fig. 3c, Supplementary Fig. 15b, c). The absorbance spectra of different TNFmAb variants in PBS were measured by an Implen, NP80touch UV-Vis spectrophotometer (Supplementary Fig. 15d). Importantly, we noted that over-reaction with Cy7-NHS or DBCO-NHS can cause TNFmAb to aggregate and precipitate from the solution; we thus recommend adding no more than 5 molar equivalents of dye-NHS or DBCO-NHS to TNFmAb solutions at similar concentrations.

#### Sodium dodecyl sulfate-polyacrylamide gel electrophoresis (SDS-PAGE)

SDS-PAGE was performed in gels made with the One-Step PAGE Gel Fast Preparation Kit (10%, Fdbio science, FD341) after the antibodies were reduced in the loading buffer (Cwbio, CW0027S). After electrophoresis, the gel was stained with Coomassie Blue staining reagent (Beyotime, P0017A). The protein ladder (Thermo Fisher, 26617) used here was a pre-stained mixture of ten recombinant proteins ranging from 10 to 180 KDa.

#### Enzyme-linked immunosorbent assay (ELIZA)

A 96-well ELIZA plate was coated with 1 μg/mL of recombinant mouse TNF-α protein (Absin, abs04259) in PBS overnight at 4 °C, followed by blocking with 2% BSA in PBS with 0.05% Tween 20 (PBS-T) for 1 h at room temperature. Then, wells were washed with PBS-T and further incubated with TNFmAb or TNFmAb-CHP at designated concentrations for 1 h at room temperature. After three rounds of washing with PBS-T, the wells were incubated with horseradish peroxidase (HRP)-coupled goat anti-rat IgG antibody (Solarbio, SE132) for 1 h at room temperature. After washing, the plate-bound antibodies were detected with a tetramethyl-benzidine (TMB, Solarbio, PR1200) substrate by subtracting the measured absorbance at 570 nm from the absorbance at 450 nm.

### Histology and microscopy

#### Tissue processing and ex vivo Cy3-CHP staining

The collected hind paw specimens were fixed in 4% PFA at room temperature overnight and washed three times with PBS. The fixed hind paw specimens were cut in half to expose the cross-section in the median sagittal plane for Cy3-CHP staining. Before staining, Cy3-CHP solution (5 μM in 2 mL PBS) was heated to 80 °C for 5 min to dissociate the triple-helix into single strands and rapidly cooled to room temperature by sitting in an ice-water bath for 1 min. The samples were incubated in Cy3-CHP solution at 4 °C for 72 h before being washed with 15 mL PBS for 30 min three times. Subsequently, the stained specimens were decalcified in 20% EDTA solution (w/v, pH = 7.0) for three days, with the EDTA solution renewed halfway. After rinsing with PBS for 15 min three times, the decalcified specimens were incubated in 30% w/v sucrose solution for 24 h and then immersed in a 30% (w/v) / 40% (v/v) / 30% (v/v) mixture of sucrose/water/optimal cutting temperature compound (OCT, Solarbio, 4583) for 12 h before being frozen in OCT for cryosectioning into 10 μm thick sections. For fluorescence imaging of bound Cy3-CHP (e.g., Fig. 1c, f, Supplementary Fig. 10), cryosectioned slides were rinsed with PBS for 5 min to remove OCT, stained with DAPI (Beyotime C1002, 1:1000 dilution in ddH_2_O) for 15 min, sealed in an antifade mounting medium (Vector, H-1000) and imaged with an EVOS M7000 imaging system (Thermo Fisher) (Light cubes: RFP and DAPI, objective: 20×). To ensure signal specificity and eliminate background interference, imaging parameters, including excitation intensity, exposure time, and most importantly, detector gain, were first calibrated using pannus sections stained with the sequence-scrambled control Cy3-^S^CHP to establish a baseline of negligible fluorescence, with these identical settings strictly maintained for all Cy3-CHP acquisitions. Pseudocolors were assigned to the images using the LUT color scheme available in the ImageJ software (Fiji 2.14.0). Adjacent sections were then stained with Safranin-O/fast green staining kit (Solarbio, G1371).

For Supplementary Fig. 7, pannus cryosections from a CAIA model mouse were washed with PBS to remove OCT. For competitive blocking, Cy5-CHP or Cy5-^S^CHP (200 µM in 100 µL of PBS) were pre-heated at 80 °C for 10 min, immediately cooled down, and applied to the sections for overnight incubation at 4 °C (Cy5-CHP: Cy5-Ahx-(GPO)_9_, m/z calculated: 3181.4 [M+Na]^+^, m/z found: 3182.1 [M+Na]^+^; Cy5-^S^CHP: Cy5-Ahx-PGOGPGPOPOGOGOPPGOOPGGOOPPG, m/z calculated: 3181.4 [M+Na]^+^, m/z found: 3182.1 [M+Na]^+^). After removing the Cy5-peptide solutions, the sections were incubated with Cy3-CHP (10 µM in 100 µL PBS) at 4 °C for 6 h, followed by DAPI staining and PBS washes. Imaging was performed on an EVOS M7000 system (20× objective, Cy3: RFP cube, Light: 0.5, Exposure: 0.25, Gain: 10).

#### Haematoxylin & eosin (H&E) and Safranin O/fast green stains

H&E and Safranin O staining of the paraffin-embedded sections was performed by Wuhan Servicebio Technology (e.g., Fig. 2i, k, 4f, Supplementary Figs. 26, 33). Stained histological slides were scanned and analyzed using a Pannoramic 250 Flash III scanner (3DHISTECH). H&E staining of mouse Achilles tendon cryosections was performed with an H&E staining kit (Phygene, PH0516) according to the manufacturer’s instructions (Supplementary Fig. 16). Cryosections of hind paws were stained with a Safranin-O/fast green staining kit according to the manufacturer’s instructions (Fig. 1f).

#### Section staining with Cy7-labeled TNFmAbs

Pannus tissue was carefully dissected from the inflamed paw of a CAIA mouse and cryosectioned into 4-μm sections. After the OCT was removed with PBS, sections were incubated in 0.3% H_2_O_2_ for 20 min, blocked with 10% goat serum for 1 h, incubated with Cy7-goat anti-rabbit IgG (Cohesion Bioscience, CSA4611), Cy7-TNFmAb, or Cy7-TNFmAb-CHP (200 μg/mL in PBS) overnight at 4 °C. Slides were mounted with antifade mounting medium the next day, followed by microscopic imaging using the EVOS M7000 imaging system (Light cube: Cy7, objective: 20×).

Paraffin-embedded sections of mouse skin and cryosections of mouse tendons were stained to test the TNFmAb-CHP conjugate’s affinity to denatured collagen (Supplementary Fig. 16). After removing the paraffin or OCT, the sections were heated in a 100 °C water bath for 1 min to purposefully denature the tissues’ collagen content. Then the sections were incubated in 0.3% H_2_O_2_ for 30 min, blocked with 10% goat serum for 1 h, and incubated with 200 μg/mL of Cy7-labeled or unlabeled TNFmAb or TNFmAb-CHP overnight at 4 °C. Sections stained with Cy7-labeled antibodies were fluorescently imaged using the Cy7 Light Cube on the EVOS M700 imaging system after washing. The sections were further incubated with HRP-conjugated anti-rat IgG (Solarbio, SE132) for 1 h at room temperature, and the tissue-bound mAbs were visualized with diaminobenzidine. All stained slides were then imaged in a bright field using the EVOS M7000 imaging system.

For the competitive blocking assay described in Supplementary Fig. 17a, pannus cryosections were first treated at 4 °C overnight with 200 µM Cy5-CHP (sequence: Cy5-Ahx-(GfO)_9_; m/z calculated: 3343.3 [M + Na]^+^, m/z found: 3343.8 [M + Na]^+^) and Cy5-^S^CHP (sequence: Cy5-Ahx-OfGGOfGfGfOfOGOfGOOfGGOOffG; m/z calculated: 3343.3 [M + Na]^+^, found: 3343.8 [M + Na]^+^), which were pre-heated at 45 °C for 5 min. The sections were then probed with Cy7-TNFmAb-CHP (200 µg/mL in 100 µL PBS) for 6 h at 4 °C, stained with DAPI, washed, and imaged using EVOS M7000 system (20× objective, Cy7: Cy7 cube, Light: 0.5, Exposure: 0.25, Gain: 20).

#### Clinical RA cartilage specimens

All experimental plans involving histological analysis of joint specimens from RA patients were approved by the Ethics Committee of The Fifth Affiliated Hospital of Sun Yat-sen University (number: K150-1). The articular cartilage specimen was surgically removed from an RA patient during a shoulder arthroplasty. The remaining cartilage was cryosectioned (5 μm thick) and used for testing Cy3-CHP or Cy7-TNFmAb-CHP binding, as described above.

#### Immunofluorescence

To detect MMPs, CTSK, and collagen types in the joint tissues, the following primary antibodies and dilution folds were used: MMP1 (1:200, Proteintech, 10371-2-AP), MMP2 (1:200, GeneTEX, GTX104577), MMP3 (1:200, Proteintech, 17873-1-AP), MMP8 (1:200, Proteintech, 17874-1-AP), MMP9 (1:200, Abcam, ab38898), MMP13 (1:200, Proteintech, 18165-1-AP), MMP14 (1:200, Abcam, ab51074, clone: EP1264Y), CTSK (1:200, Santa Cruz, SC-48353, clone: E-7), Col I (1:200, Abcam, ab34710), and Col IV (1:200, Abcam, ab6586). Cryosections of the pannus harvested from CAIA mice were stained (Fig. 1d, Supplementary Figs. 6, 8). Briefly, after the OCT was removed, the sections were blocked with 10% goat serum for 30 min and incubated with MMPs and collagen antibodies diluted in pre-heated 10 μM Cy3-CHP (in 1% goat serum) overnight at 4 °C; for the negative control, sections were incubated with 10 μM sequence-scrambled Cy3-^S^CHP in the absence of primary antibodies at this step. The staining of CTSK was performed using a mouse antibody (Santa Cruz, SC-48353). After serum blocking, the sections were further immuno-blocked with a Fab fragment against mouse IgG (H + L) (1:40 in 1% FBS, Jackson, 115-007-003) at room temperature for 2 h, followed by overnight staining with the anti-CTSK antibody diluted in pre-heated 10 μM Cy3-CHP (in 1% FBS) at 4 °C. After washing with PBS three times the next day, all sections were stained with an Alexa Fluor 647-conjugated secondary antibody (goat anti-rabbit IgG H&L, 1:1000, Jackson, 111-605-003) for 1 h at room temperature. After washing, the sections were stained with DAPI for 15 min and sealed in an antifade mounting medium. Cryosections of the hind paws pre-stained with Cy3-CHP before sectioning were also used and probed with primary antibodies targeting MMPs, CTSK, and Col I 3/4 fragments (Adipogen Life Sciences, AG-25T-0116-C025, 1:200) overnight at 4 °C (Fig. 1e, Supplementary Figs. 8–10) as described above. Stained slides were imaged using the EVOS M7000 imaging system (light cubes: RFP, Cy5, and DAPI) with the following parameters (Table 1). Pseudo colors were assigned to the images using the LUT color scheme available in the ImageJ software.

**Table 1:** Imaging channels and parameters for antibodies and probes

Imaging parameters	Light cube		pannus			cartilage		
		objective	Light	Exposure	Gain	Light	Exposure	Gain
2^nd^ antibody only	Cy5	20×	0.5	0.25	6	0.5	0.25	10
MMP1	Cy5	20×	0.5	0.25	6	0.5	0.25	2
MMP2	Cy5	20×	0.5	0.25	2	0.5	0.25	4
MMP3	Cy5	20×	0.5	0.25	5	0.5	0.25	6
MMP8	Cy5	20×	0.5	0.25	6	0.5	0.25	4
MMP9	Cy5	20×	0.5	0.25	1	0.5	0.2	1
MMP13	Cy5	20×	0.5	0.25	6	0.5	0.25	4
MMP14	Cy5	20×	0.5	0.25	6	0.5	0.25	10
CTSK	Cy5	20×	0.5	0.25	2	0.5	0.25	4
Col I	Cy5	20×	0.5	0.25	2	/	/	/
Col IV	Cy5	20×	0.5	0.25	5	/	/	/
Col I ¾ fragment	Cy5	10x	0.5	0.25	2	/	/	/
Cy3-CHP	RFP	10/20×	0.5	0.25	10	0.5	0.25	4
Cy3-^S^CHP	RFP	10/20×	0.5	0.25	10	/	/	/

#### Tissue clearing and light-sheet fluorescence microscopy

Two hours post-injection of Cy7-Ahx-K-Ahx-(GfO)_9_, mice were transcardially perfused with 0.02% heparin in PBS (m/v) and 4% PFA. Next, hind paws were collected and cleared following the PEGASOS method^39^ with slight modifications (Supplementary Fig. 4). After fixation in 4% PFA for 24 h at room temperature and removal of the skin, hind paw samples were decalcified in 20% EDTA for 1 week at room temperature, with the solution changed once a day. After being washed three times with PBS (1 h each round), the samples were degreased with a gradient (30%, 50%, 70%) of tert-butanol (Macklin, T819475) and followed by dehydration with 70% v/v tert-butanol, 27% v/v PEGMEMA500 (Sigma-Aldrich, 447943) and 3% w/v Quadrol (Sigma-Aldrich, 122262) for 2 days. Finally, the samples were immersed in a clearing medium (BB-PEG) made by 75% v/v benzyl benzoate (Sigma-Aldrich, W213802), 22% v/v PEGMEMA500, and 3% w/v Quadrol for at least 1 day. Care was taken to avoid light during the entire clearing process.

Each cleared hind paw specimen was imaged on a LaVision Biotec Ultramicroscope Blaze. The tissue was immersed in the imaging chamber filled with the BB-PEG medium. Each cleared specimen was scanned with a magnification of 4 on both sides, and each side was composed of three light-sheet beams with a 4 μm step in the Z-axis. The images were acquired by continuous light-sheet scanning and stitched using a blending algorithm on both sides. The optical filters ex/em were as follows: autofluorescence, 470/525 nm; Cy7, 710/810 nm. Images were acquired by ImSpector software (version 7.6.3, LaVision), saved as 16-bit grayscale TIFF images for each channel, and reconstructed with the Imaris software (version 9.8.0, Oxford Instruments). Movies were produced at a frame rate of 25 fps (Supplementary Movies).

Tracking Cy7-TNFmAb-CHP localization in RA paws was performed in the single-paw CAIA model. On day 1 or 2 post local LPS injection in the right hind paw, Cy7 labeled TNFmAb or TNFmAb-CHP (30 μg in 100 μL of PBS) was injected into the tail vein of each mouse. The mice were euthanized 2 h post i.v. injection of the Cy7-TNFmAbs and transcardially perfused, after which their hind paws were collected and cleared for light-sheet microscopy imaging as described above (Fig. 5, Supplementary Figs. 27, 28).

### In vivo near-infrared fluorescence imaging

Cy7-CHP or Cy7-^S^CHP (1 nmole in 100 μL of PBS) was injected into the tail vein of each mouse 2 h prior to fluorescence imaging using an IVIS Spectrum imager (PerkinElmer Lumina III). The animals in Fig. 1a were euthanized, and their hind paw specimens were collected and imaged after skin removal (Supplementary Fig. 3). For Figs. 2f–k, 4, the animals were euthanized after in vivo fluorescence imaging, and their hind paw specimens were collected for subsequent histological analysis. In Fig. 3f, Cy7-labeled TNFmAb or TNFmAb-CHP (30 μg in 100 μL of PBS) was injected into the tail vein of each mouse. Fluorescence imaging was performed before and 0.5, 1, 2, 3, 6, 12, 24, 48, and 72 h after the injection (Fig. 3f, g, Supplementary Fig. 18) with the following parameters: f/stop, 2; ex/em, 720/790 nm; exposure time, 1 s; binning, 4. All fluorescence images were analyzed using the Living Image software (PerkinElmer). The total or average fluorescence signal from each paw was measured by quantifying the radiant efficiency from the entire region of the paws (e.g., Fig. 2c).

### In vivo efficacy study

#### Therapeutic assessment

The study was performed in the regular CAIA model. Prior to intraperitoneal injection of LPS on day 0, mice were intravenously injected with non-fluorescently labeled TNFmAb or TNFmAb-CHP at a dose of 15 mg/kg per mouse (Fig. 4a). Joint swelling was scored, and paw thickness was monitored daily (Fig. 4b, c). On day 7, Cy7-CHP was injected into the tail vein of the mice, which were fluorescently imaged in vivo (Fig. 4d, e) before being euthanized. Their hind paws were collected for histological analysis (Fig. 4f, g). All paws were embedded in paraffin, sectioned to 4 μm thick and stained with H&E and Safranin-O. The H&E-stained slides were scored by pathologists blinded to the tests. The criteria used to evaluate the severity of synovial hyperplasia and bone resorption in different joints^68^ were as follows: 0, normal synovium; 1, hypertrophy and cell invasion present in the synovial membrane; 2, pannus and cartilage erosions present; 3, moderate erosions of cartilages and subchondral bone; and 4, severe infiltration associated with total or near total destruction of joint architecture. The scores in both hind paws were summed for each mouse (score per mouse total, 0 to 8). Immunofluorescence staining for CD86, CD206, and IL-10 was performed on paraffin-embedded sections of the ankles from these CAIA mice by Wuhan Servicebio Technology and imaged using an EVOS M7000 system (Thermo Fisher). TNFmAb-CHP (intravenously injected, 15 mg/kg per mouse) was also tested against TNFmAb in CAIA mice after their average arthritis scores progressed to 1 or 2 per paw (from 1 or 2 days post-LPS induction), where paw swelling was monitored daily for 7 days post-treatment (Supplementary Figs. 24, 25). All paw scoring and measurements were conducted in a blinded manner, where the investigator responsible for arthritis scoring was kept unaware of the specific treatment group allocations.

#### Biosafety assessment

On day 7 (endpoint) of the therapeutic efficacy study, the vital organs of each mouse (heart, liver, spleen, lung, and kidney) were collected, processed into formalin-fixed paraffin-embedded slides, and stained with H&E for histologic evaluation (Supplementary Fig. 33).

### Pharmacokinetics and biodistribution

CAIA mice (average total arthritis score per mouse: 7, *n* = 4 mice) and normal mice (*n* = 4 mice) received a single i.v. injection of Cy7-TNFmAb or Cy7-TNFmAb-CHP (30 μg in 100 μL of PBS), followed by serial retro-orbital blood collection at 0.2, 2, 4, 8, 24, 48, 72, and 96 h post-injection. Plasma samples were obtained by centrifuging the blood at 22 g (1000 rpm) for 10 min at 37 °C, and were transferred to a black 96-well plate (50 μL/well) to be fluorescently imaged using an IVIS Spectrum system (f/stop, 2; ex/em, 720/790 nm; exposure time, 1 s; binning, 4) and measured using a microplate reader (Tecan Infinite Plex; ex/em, 750/790 nm; Gain, 200). The concentration of Cy7-TNFmAb or Cy7-TNFmAb-CHP in each plasma sample was quantified by interpolating fluorescence values against respective standard curves.

For Supplementary Fig. 2d, Supplementary Fig. 5c, CAIA mice (average total arthritis score per mouse: 6.3, *n* = 3 mice) and normal control mice (*n* = 3 mice) received a single i.v. injection of 1 nmole of Cy7-CHP, (or Lys-modified-Cy7-CHP). Blood was collected at 0.2, 2, 4, 8, 12, and 24 h. Plasma samples were analyzed using the IVIS Spectrum system under the same imaging settings as described above.

CAIA mice (average total arthritis score per mouse: 7, *n* = 3 mice) received a single i.v. injection of Cy7-TNFmAb or Cy7-TNFmAb-CHP (30 μg in 100 μL of PBS), and were euthanized 24 h post-injection. The heart, liver, spleen, lungs, kidneys, stomach, intestines, uterus, fore paws, hind paws, and blood of each mouse were collected and fluorescently imaged using an IVIS Spectrum system (f/stop, 2; ex/em, 720/790 nm; exposure time, 1 s; binning, 4). The biodistribution of the Cy7-labeled mAbs was estimated based on the average fluorescence radiant efficiency from an identical region-of-interest for each organ/tissue measured across all mAb-dosed mice using the Living Image software after removing the value of the same organ/tissue of a CAIA mouse without any mAb injection. For Supplementary Fig. 5d, CAIA mice (average total arthritis score per mouse: 6.5, *n* = 2 mice) received a single i.v. injection of 1 nmole of Cy7-CHP or Lys-modified-Cy7-CHP. The mice were euthanized at 2 h post-injection, and the same set of tissues was collected and imaged.

### Statistics and reproducibility

All data in this study were analyzed and plotted using the Graphpad Prism software (version 10.0.0, Dotmatics). All statistical tests were two-sided. Data were presented as mean and standard deviation (normally distributed data, i.e., Fig. 1a) or median with interquartile range (non-normally distributed data, i.e., Fig. 2j), as indicated in the figure legends. For comparisons between two means of normally distributed data showing homoscedasticity, a two-sided unpaired Student’s *t*-test was used (i.e., Fig. 3d). For multiple group comparisons with normally distributed data, one-way ANOVA with post-hoc Tukey’s test (homoscedasticity) or Welch’s ANOVA with post-hoc Dunnett’s T3 test (heteroscedasticity) was used. Datasets with non-normal distributions were analyzed using the Mann-Whitney U test. One-way ANOVA with repeated measures was utilized for data across time points (normally distributed data, i.e., Fig. 1a), while the Friedman test for repeated measures on ranks was utilized for data across time points (non-normally distributed data, i.e., Fig. 4b). The Spearman correlation coefficients (for ordinal variables, e.g., arthritis score) were calculated to evaluate the correlation (i.e., Fig. 2e). *P* values below 0.05 were regarded as statistically significant, and exact *P* values are marked in the figures. *P*-values above 0.05 are not shown or marked as ns (not significant). Statistical methods, *P*-values, and sample size are indicated in figures and figure legends. All immunofluorescence and microscopy images shown are representative of at least three independent experiments.

### Reporting summary

Further information on research design is available in the Nature Portfolio Reporting Summary linked to this article.

## Supplementary information

Supplementary Information

Transparent Peer Review file

Reporting Summary

Description of Additional Supplementary Files

Supplementary Movie 1

Supplementary Movie 2

## Source data

Source data

## Data Availability

The main data supporting the results of this study are available within the paper and its supplementary information. Source data are provided with this paper.
