# Supplementary material for: Collagen hybridizing peptide imaging and delivery of therapeutic antibody in rheumatoid arthritis models
Source: Nat Commun. 2026 Apr 20;17:5423. doi: 10.1038/s41467-026-72038-y (PMC13279783; doi:10.1038/s41467-026-72038-y)
Supplement: Supplementary file 1 — Supplementary Information [file 41467_2026_72038_MOESM1_ESM.pdf]

Supplementary Information

Collagen hybridizing peptide for imaging and delivery of therapeutic antibody in rheumatoid arthritis models

Xiaoyun Mo[1,#], Yongjie Huang[1,2,#], Haimin Chen[1], Junwen Huang[3], Kui Huang[1], Suwen Zhao[1], Xufei Wang[1], Kuibo Zhang[3,*], and Yang Li[1,*]

[1]Guangdong Provincial Engineering Research Center of Molecular Imaging, Guangdong-Hong Kong-Macao University Joint Laboratory of Interventional Medicine, the Fifth Affiliated Hospital, Sun Yat-sen University, Zhuhai, Guangdong 519000, China.

[2]Department of Pathology, The Affiliated Qingyuan Hospital (Qingyuan People's Hospital), Guangzhou Medical University, Qingyuan, Guangdong 511518, China.

[3]Department of Spine Surgery, the Fifth Affiliated Hospital, Sun Yat-sen University, Zhuhai, Guangdong 519000, China.

[#]These authors contributed equally to this work: Xiaoyun Mo, Yongjie Huang

*Corresponding authors: liyang266@mail.sysu.edu.cn (Y.L.); zhangkb@mail.sysu.edu.cn (K.Z.)

This PDF file includes:

Supplementary Figures 1 to 33

Supplementary Tables 1 to 2

Supplementary Figures

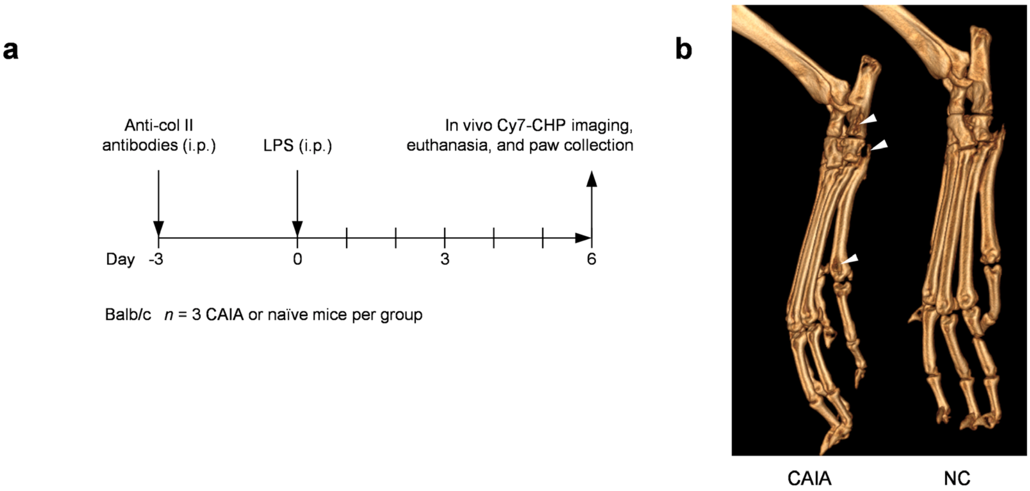

Supplementary Fig. 1. a Typical timeline of our mouse study of the CAIA model of RA, induced by an intraperitoneal (i.p.) injection of a cocktail of anti-collagen II antibodies (1.5 mg/mouse) followed by an i.p. injection of lipopolysaccharide (LPS, 25 µg) 3 days later (on day 0). For the results in Fig. 1a, normal or CAIA mice were intravenously (i.v.) injected with 1 nmole of Cy7-CHP (or Cy7-^{S}CHP) on day 6, 2 h before in vivo near-infrared fluorescence imaging, after which the mice were euthanized and their joint specimens were collected for further imaging (Figs.3) and histology. **b** Representative three-dimensional views of reconstructed micro-computed tomography (micro-CT) scans of the hind paws from normal control (NC) and CAIA mice. Arrowheads: regions of bone erosion.

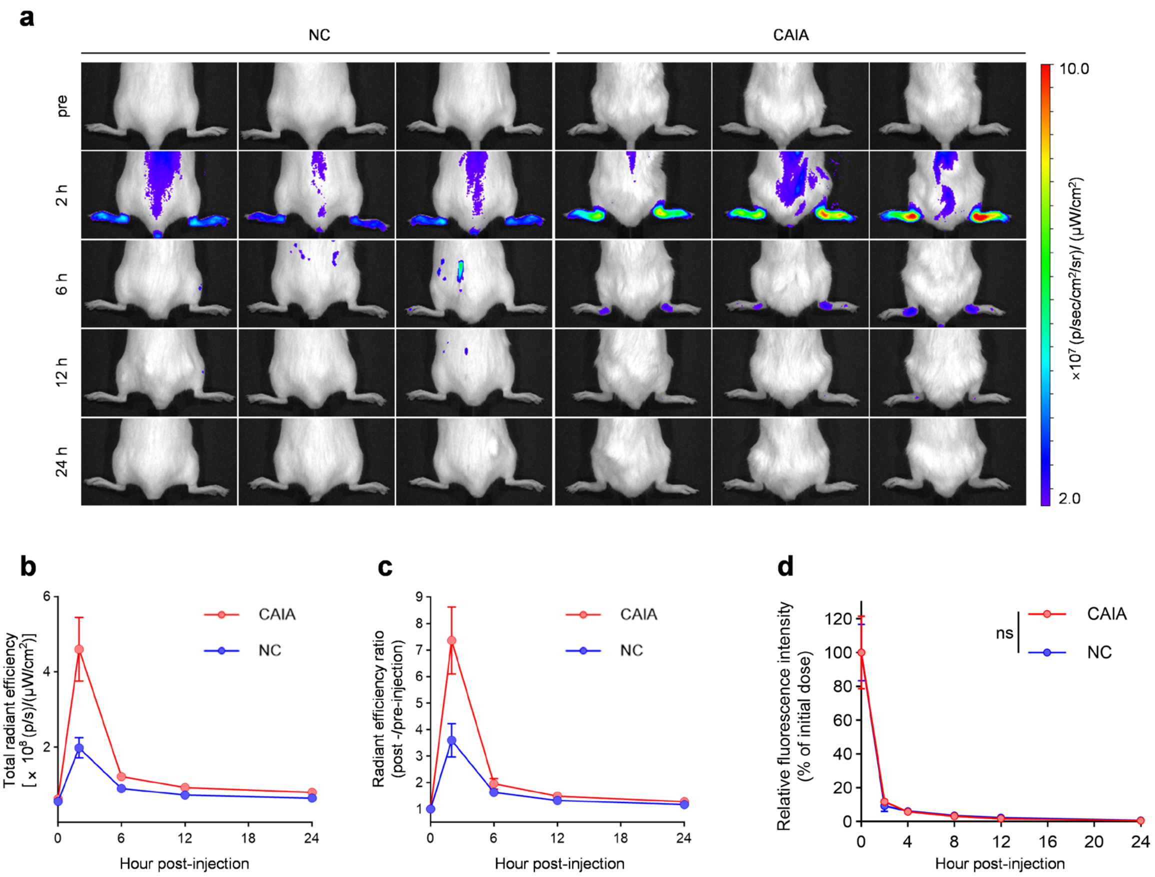

Supplementary Fig. 2. a In vivo fluorescence images of the CAIA or normal control mice before and 2, 6, 12, and 24 h post i.v. injection of 1 nmole of Cy7-CHP (n = 3 mice). As evidenced by the quantified fluorescence signals (**b**) and the remaining ratios (**c**) in the hind paws, with the 1 nmole i.v. dose, the Cy7-CHP uptake in the ankles peaked within 2 h post-injection and decreased to the baseline level within 24 h. **d** Fluorescence intensity of Cy7-CHP in plasma from NC and CAIA mice (tested on day 6 post-LPS injection) from 0.2 to 24 h post-injection, normalized to the data obtained at 0.2 h (n = 3 mice). Numbers were expressed as mean ± standard deviation (**b**–**d**). Statistical analysis: paired t-test (**d**).

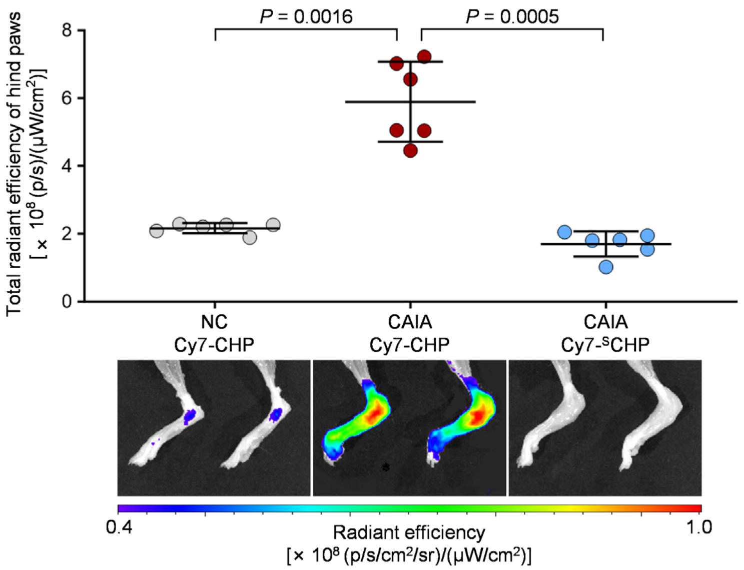

Supplementary Fig. 3. Representative fluorescence images and signal quantification of the hind paws from the NC and CAIA mice after paw dissection and skin removal. The mice were sacrificed 2 h post i.v. injection of 1 nmole Cy7-CHP or Cy7-^{S}CHP on day 6 post-LPS injection (n = 3 mice). Data points: individual hind paws. Numbers were expressed as mean ± standard deviation. Statistical analysis: Welch's ANOVA, Dunnett's T3 multiple comparisons test.

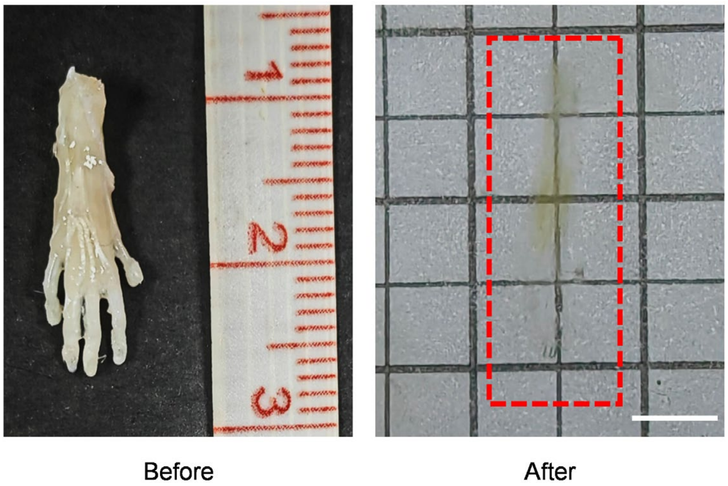

Supplementary Fig. 4. A mouse hind paw specimen before and after tissue clearing using the PEGASOS method. Ruler unit: cm (left panel). Scale bar: 5 mm.

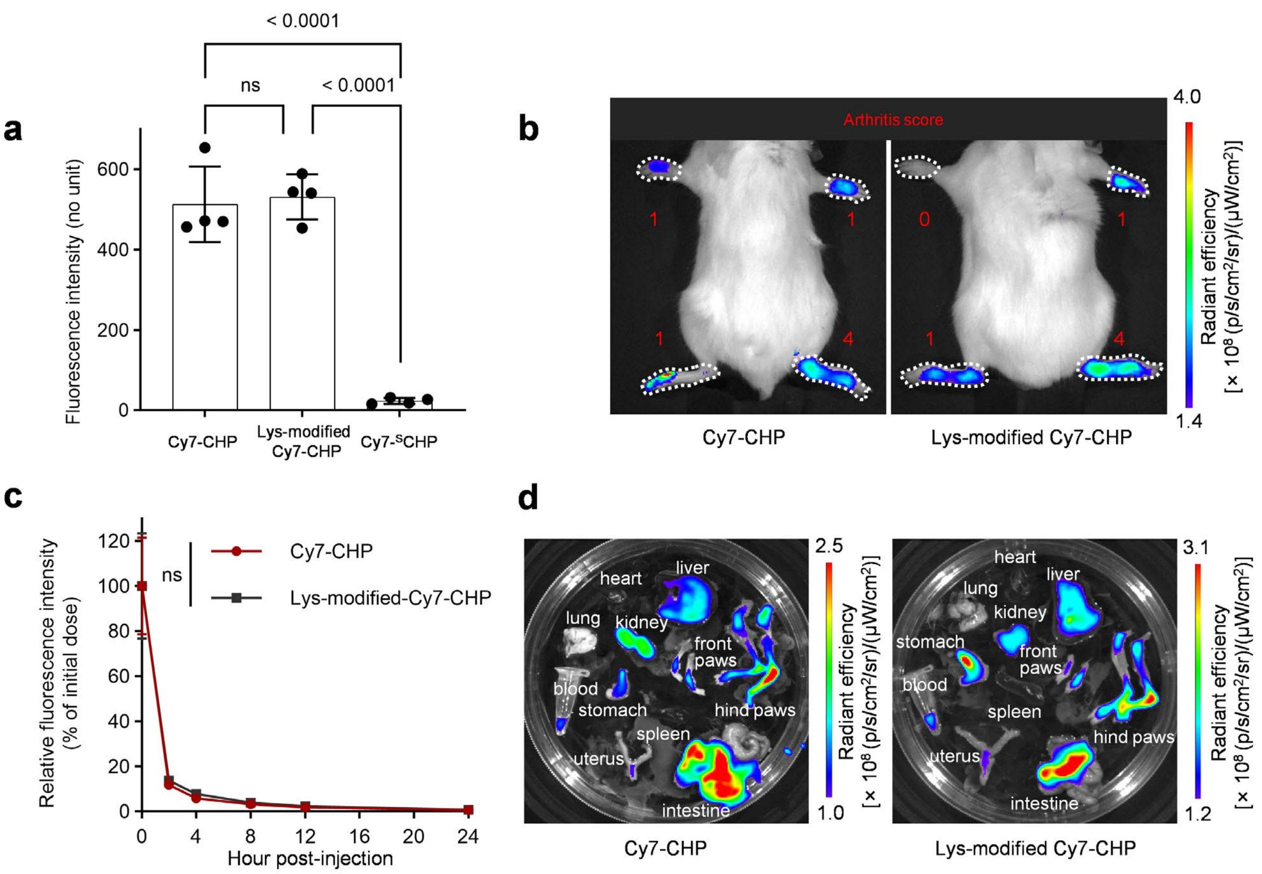

Supplementary Fig. 5. a Fluorescence gelatin binding experiments. Cy7-^{S}CHP: sequence-scrambled control peptide. **b** Representative in vivo images of CAIA mice showing each paw's fluorescence uptake of Cy7-CHP or Lys-modified-Cy7-CHP [sequence: Cy7-Ahx-K-Ahx-(GfO)$_9$, utilized for Fig. 1b] 2 h post i.v. injection of 1 nmole of probes. **c** Fluorescence intensity of Cy7-CHP or Lys-modified-Cy7-CHP in the plasma of CAIA mice (tested on day 6 post-LPS injection) from 0.2 to 24 h post-injection, normalized to the data at 0.2 h. **d** Representative fluorescence images of the major organs, paws, and blood of CAIA mice (tested on day 6 post-LPS injection) 2 h post i.v. injection of 1 nmole Cy7-CHP or Lys-modified-Cy7-CHP. The fluorescence signals observed in the stomach and intestine originate from the autofluorescence of the chlorophyll-containing diet. Data points: individual wells (**a**) or individual mice (**c**, n = 3). Numbers were expressed as mean ± standard deviation (**a**, **c**). Statistical analysis: one-way ANOVA with Tukey's test (**a**), paired t-test (**c**).

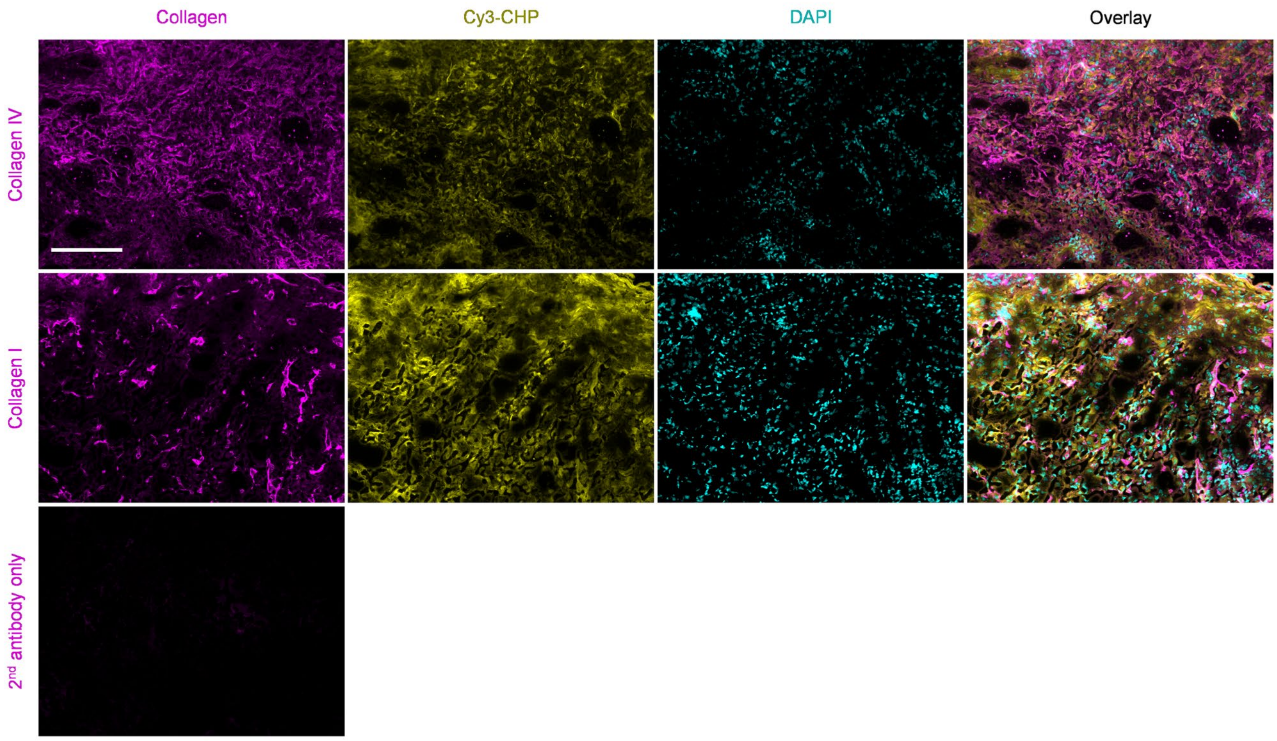

Supplementary Fig. 6. Representative fluorescence micrographs of the cryosections of the ankle synovial pannus from the CAIA mice. Each section was co-stained with DAPI, Cy3-CHP, and an antibody against collagen I or IV. All primary antibodies were detected with an Alexa Fluor 647 labeled secondary antibody. The sections in the 2nd-antibody-only group were stained without primary antibodies. Representative images from three independent experiments with similar results. Scale bar: 150 μm.

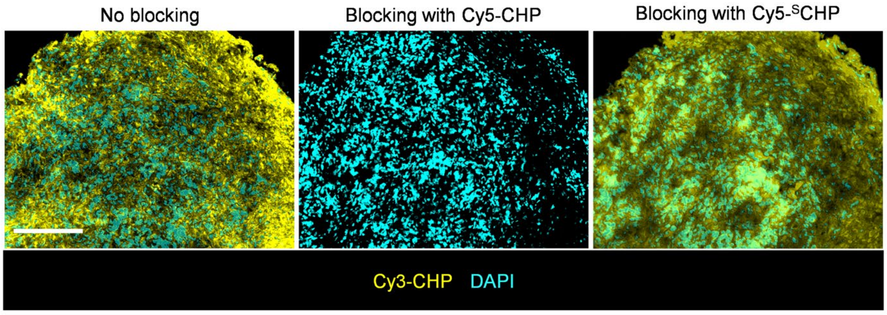

Supplementary Fig. 7. Fluorescence micrographs of neighboring cryosections from the ankle synovial pannus of CAIA mice, all stained with Cy3-CHP (10 μM) at 4 °C. The sections were pre-treated with PBS (no blocking), or excessive (200 μM) Cy5-CHP or the sequence-scrambled control peptide Cy5-^{S}CHP overnight at 4 °C. Representative images from three independent experiments with similar results. Scale bars: 150 μm.

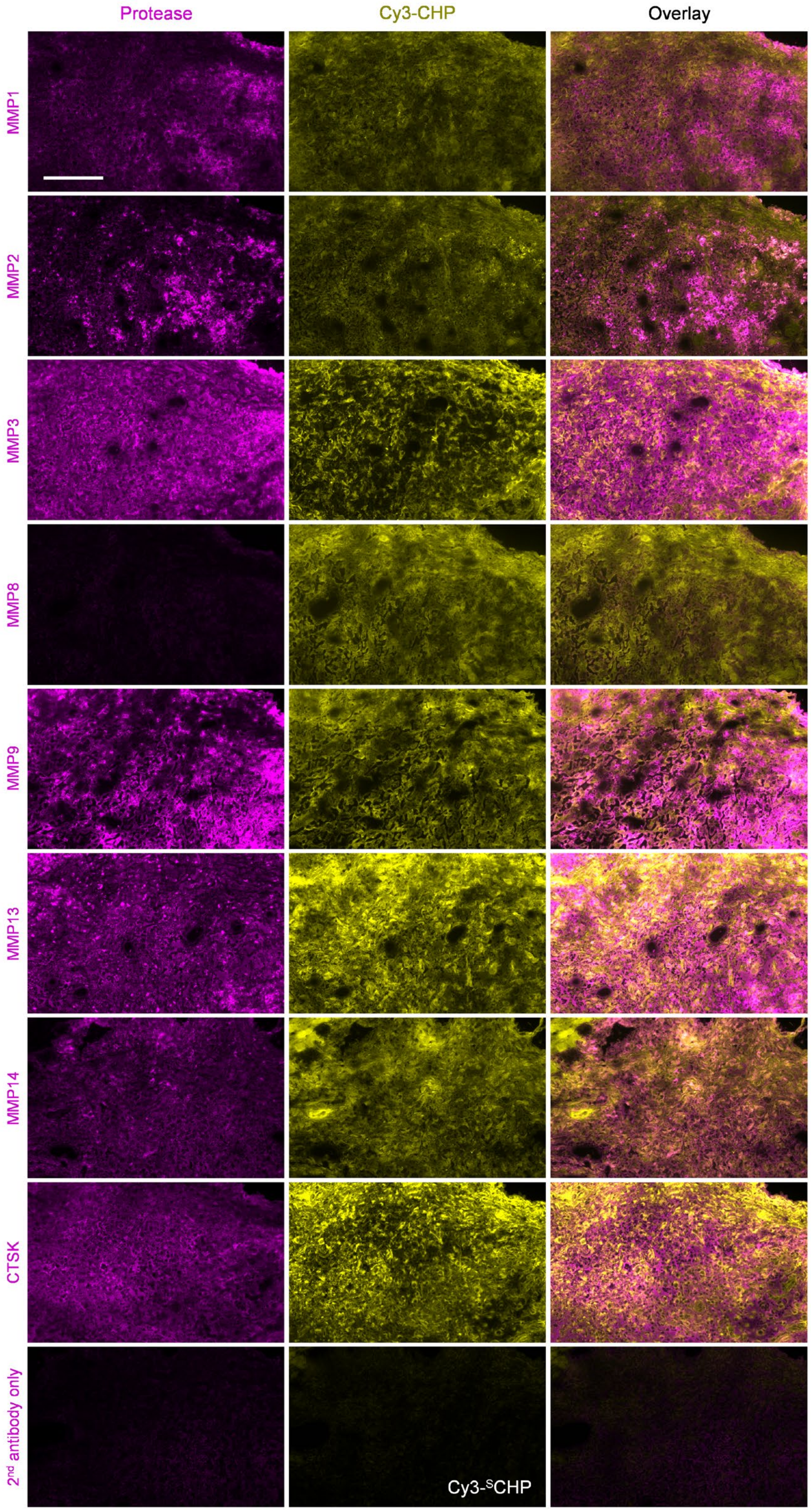

Supplementary Fig. 8. Representative fluorescence micrographs of the cryosections of the ankle synovial pannus from the CAIA mice. Each section was co-stained with Cy3-CHP (or Cy3-^{S}CHP for one set of sections as a negative control, bottom panels) and an antibody against an MMP subtype or cathepsin K (CTSK). All primary antibodies were detected with an Alexa Fluor 647-labeled secondary antibody. The sections in the 2nd-antibody-only group were stained without primary antibodies. Representative images from three independent experiments with similar results. Scale bar: 150 μm.

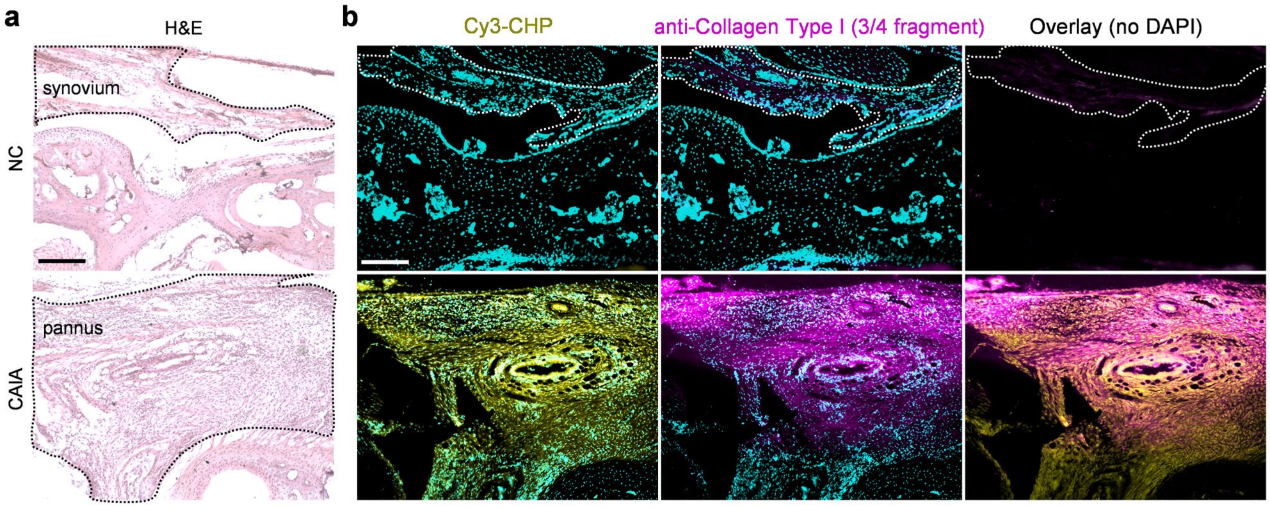

Supplementary Fig. 9. **a** Representative H&E staining of the cryosections of the normal and CAIA mice's ankle tissue. **b** Representative fluorescence micrographs of the cryosections of the normal and CAIA mice's ankle tissue that were co-stained with Cy3-CHP and an antibody against degraded collagen I fragment, which was detected with an Alexa Fluor 647 labeled secondary antibody (goat anti-rabbit IgG H&L). Cyan: DAPI. Representative images from three independent experiments with similar results. Scale bars: 250 μm (**a**, **b**).

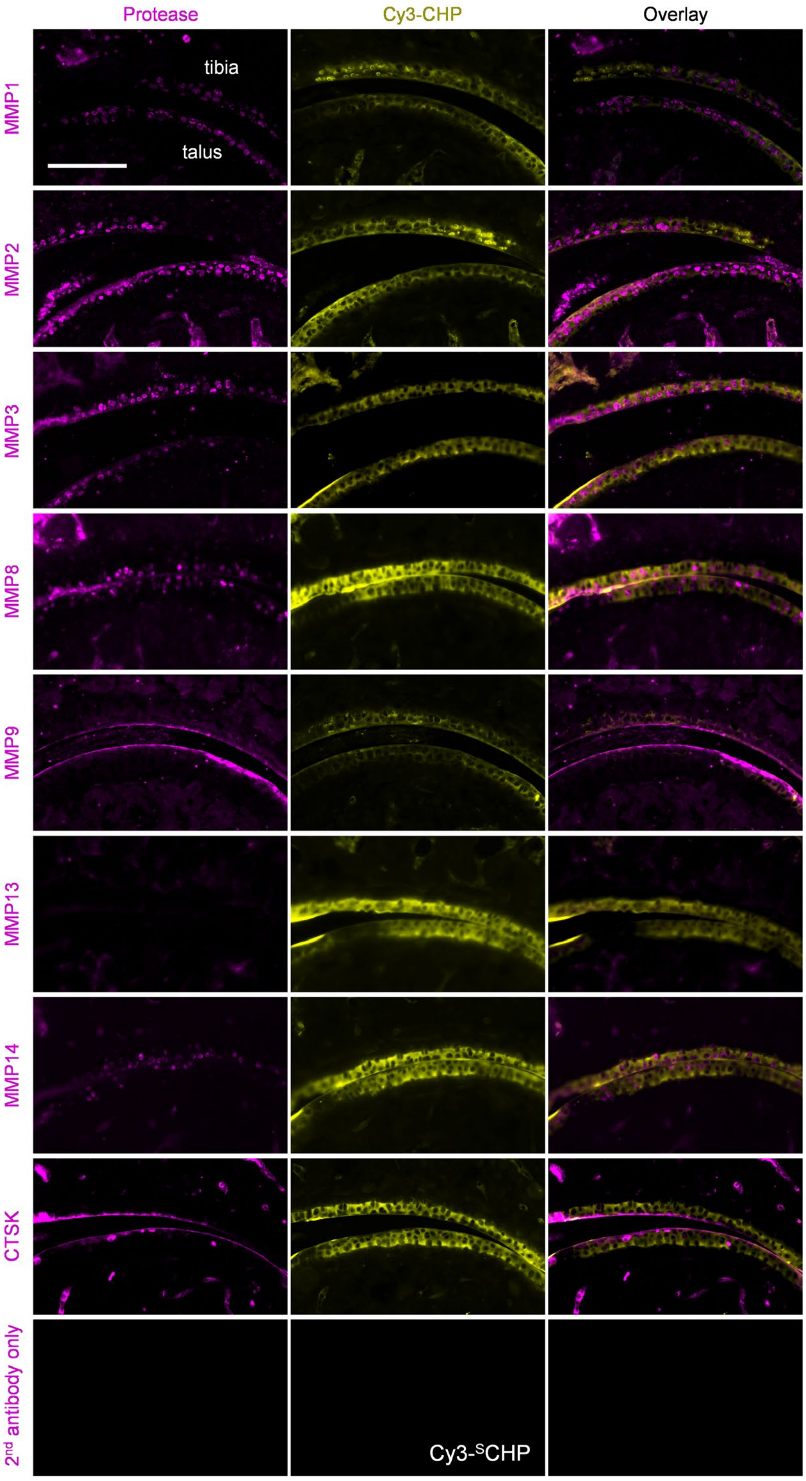

Supplementary Fig. 10. Representative fluorescence micrographs of the cryosections of the cartilage between the tibia and talus from the inflamed ankles of the CAIA mice. The paws were stained with Cy3-CHP (presumably capable of binding to denatured collagen II in the cartilage) (or Cy3-^{S}CHP for one paw as a negative control, bottom panels) before decalcification and stained with an antibody against an MMP subtype or CTSK on the cryosections. All primary antibodies were detected with an Alexa Fluor 647 labeled secondary antibody. The sections in the 2nd-antibody-only group were stained without primary antibodies. Representative images from three independent experiments with similar results. Scale bar: 150 µm.

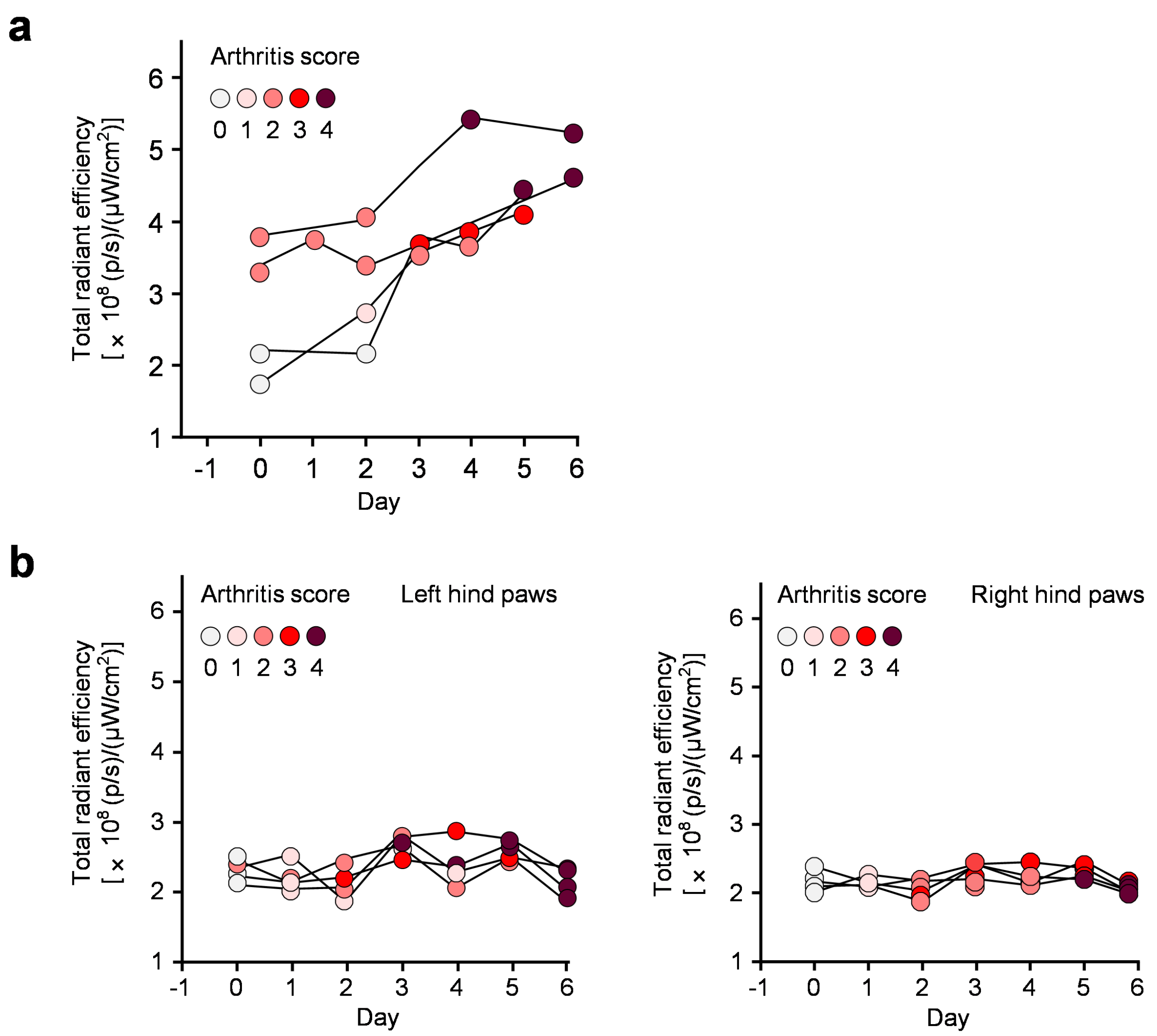

Supplementary Fig. 11. **a** Quantified in vivo Cy7-CHP fluorescence signals in the left hind paw of each CAIA mouse in Fig. 2d from the day before to 6 days after the LPS injection. **b** Quantified in vivo Cy7-^{S}CHP fluorescence signals in the left and right hind paws of each CAIA mouse from the day before to 6 days after the LPS injection. The left and right paws were shown in separate figures to avoid over-crowding and to allow tracking of the changes of induvial paws. Data points: individual hind paws.

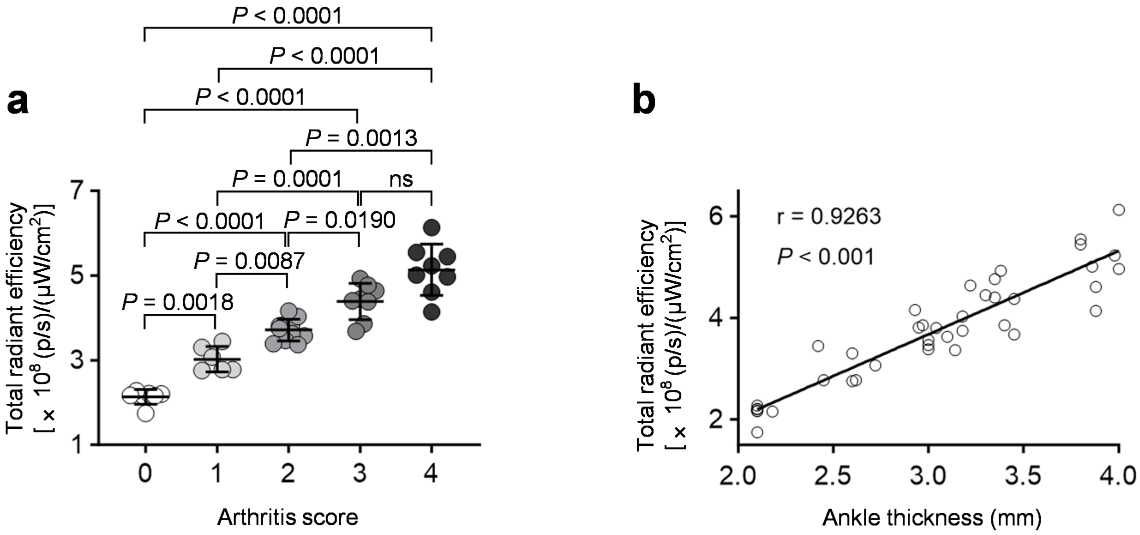

Supplementary Fig. 12. a Quantified Cy7-CHP fluorescence signals in the hind paws from all the in vivo images of the four CAIA mice studied in Fig. 2a, plotted according to the arthritis score. Numbers were expressed as mean ± standard deviation. Statistical analysis: Welch's ANOVA with Dunnett's T3 multiple comparisons test. **b** Correlation analysis between the Cy7-CHP fluorescence uptake and the corresponding ankle thickness of all 8 hind paws of 4 mice studied in Fig. 2a. Statistical analysis: Pearson correlation coefficient. Data points: individual hind paws (**a**, **b**).

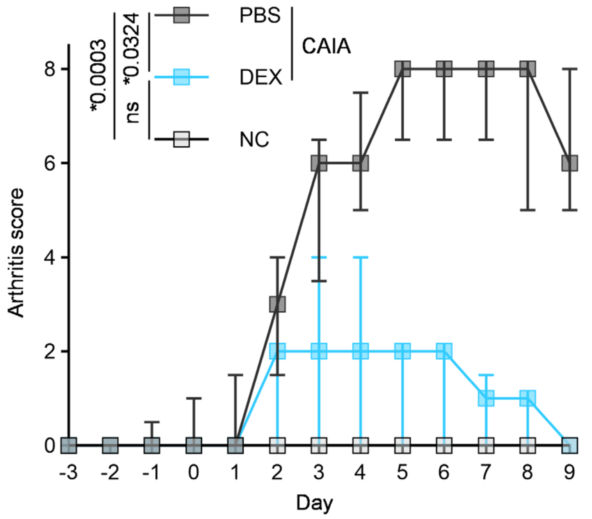

Supplementary Fig. 13. Arthritis scores of the hind paws of the NC mice or the CAIA mice treated with DEX or PBS throughout the study in Fig. 2f. Data were expressed as median with interquartile range. Statistical analysis: Friedman test for repeated measures on ranks and Dunn's multiple comparisons.

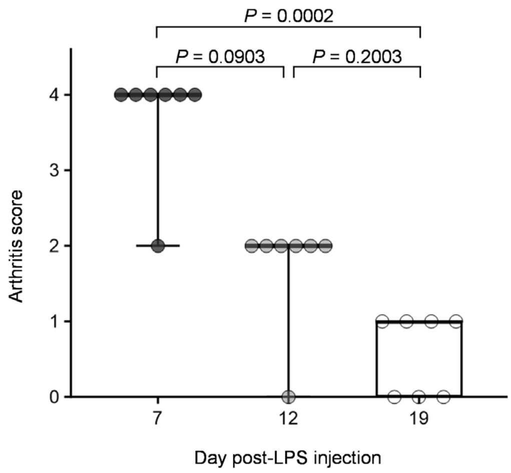

Supplementary Fig. 14. Arthritis scores of the seven inflamed hind paws of the DEX treated CAIA mice studied in Fig. 2l. Data points: individual hind paws. Numbers were expressed as median with interquartile range. Statistical analysis: Kruskal-Wallis test with post hoc Dunn's test.

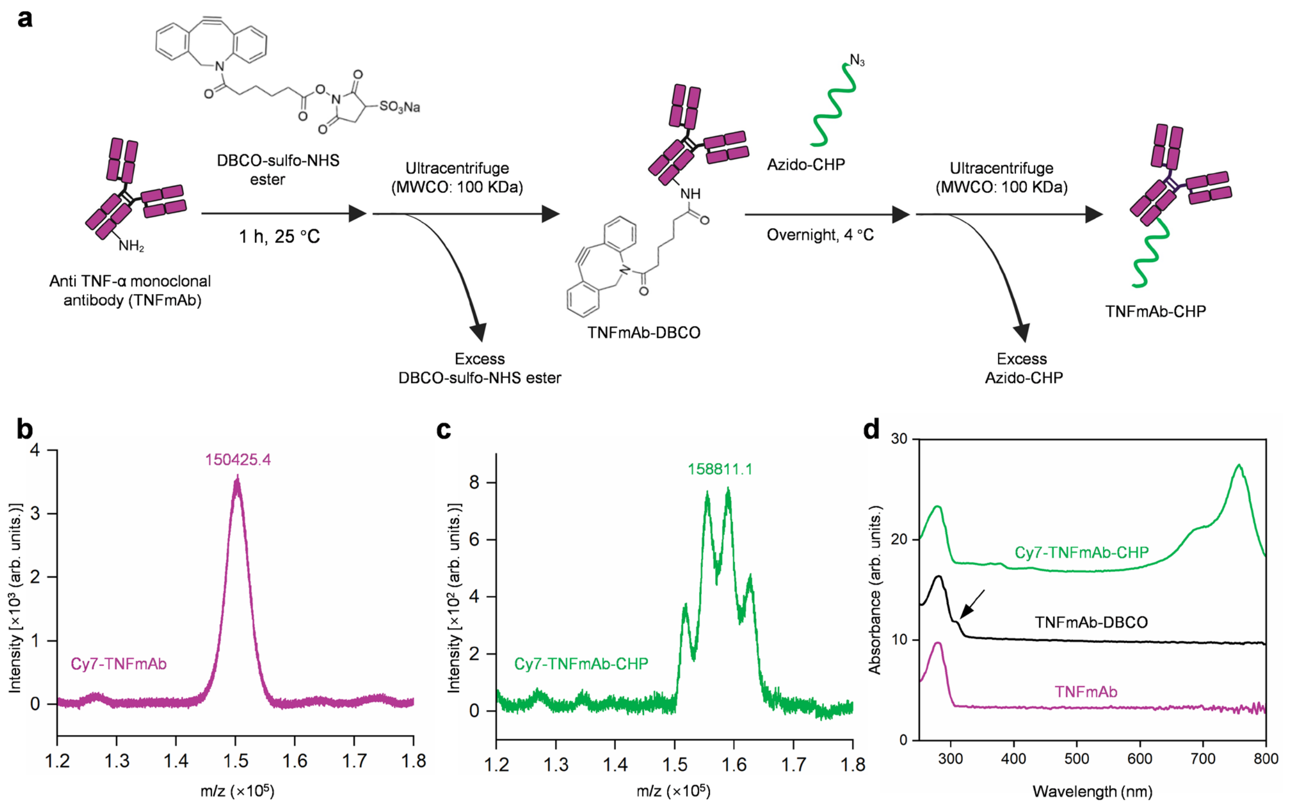

Supplementary Fig. 15. a Scheme of the bioconjugation of an azido-featuring CHP peptide to a DBCO-sulfo-NHS modified TNFmAb molecule through a DBCO-azide click reaction. **b-c** MALDI-MS spectra of Cy7-TNFmAb (**b**) and Cy7-TNFmAb-CHP (**c**). **d** UV-Vis absorbance spectra of native TNFmAb, the DBCO-coupled TNFmAb intermediate product, and the final Cy7-TNFmAb-CHP conjugate in PBS buffer, showing the characteristic DBCO absorbance peak for TNFmAb-DBCO at 310 nm (arrow). After coupling TNFmAb-DBCO with Cy7-K(N_3)-Ahx-(GfO)$_9$, the DBCO peak disappeared while a new Cy7 absorbance peak emerged near 750 nm, indicating the formation of the Cy7-TNFmAb-CHP product.

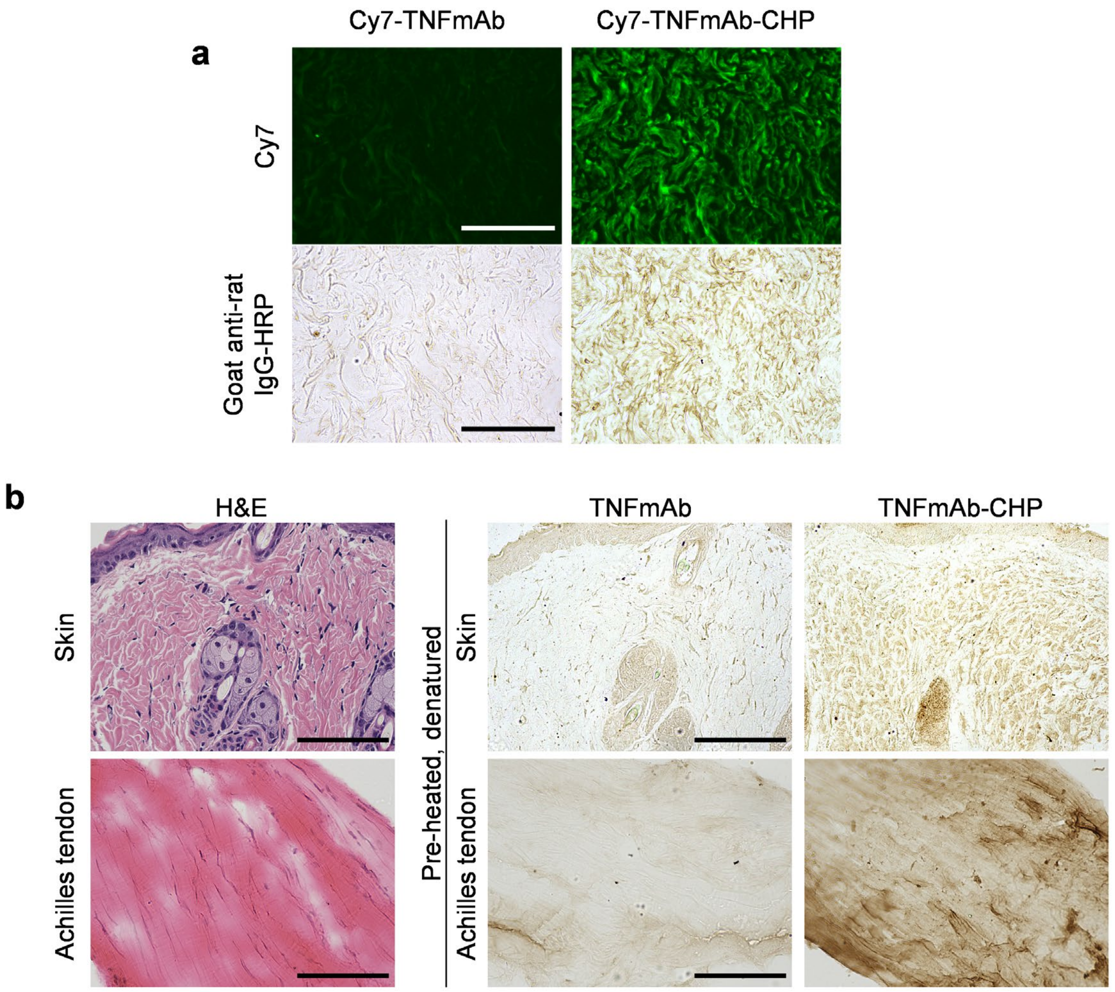

Supplementary Fig. 16. a To test the collagen-hybridizing capacity of the CHP-conjugated anti-TNF-α monoclonal antibody (a rat anti-mouse TNF-α IgG1), paraffin-embedded sections of normal mouse skin were pre-heated in a boiling water bath for 1 min after de-paraffinization to denature their collagen content. Subsequently, the sections were stained with fluorescently labeled Cy7-TNFmAb-CHP or Cy7-TNFmAb. After rinsing, pronounced Cy7 fluorescence was detected only in the dermis of the section stained with Cy7-TNFmAb-CHP. Next, tissue-bound TNFmAb in these very sections was further detected with horseradish peroxidase (HRP) conjugated secondary antibody (a goat anti-rat IgG) followed by a DAB chromogenic reaction. As expected, strong brown coloration was seen only in the dermis of the Cy7-TNFmAb-CHP-stained section. **b** We also tested the affinity to denatured collagen for the non-fluorescent, unlabeled TNFmAb-CHP histologically. Paraffin-embedded sections of normal mouse skin were deparaffinized and cryosections of normal mouse Achilles tendon were rinsed with PBS to remove the OCT embedding compound before they were heat-denatured in a boiling water bath for 1 min. The sections were then incubated with unlabeled TNFmAb-CHP or TNFmAb, which were subsequently detected by HRP-conjugated secondary antibody and DAB color development. Marked brown coloration was noted only in the TNFmAb-CHP treated sections. Adjacent sections were stained with H&E for reference. These binding tests on purposefully heat-denatured sections indicated that CHP-conjugation confers the TNFmAb affinity to the denatured collagen matrix in tissues. Representative images from three independent experiments with similar results. Scale bar: 100 μm.

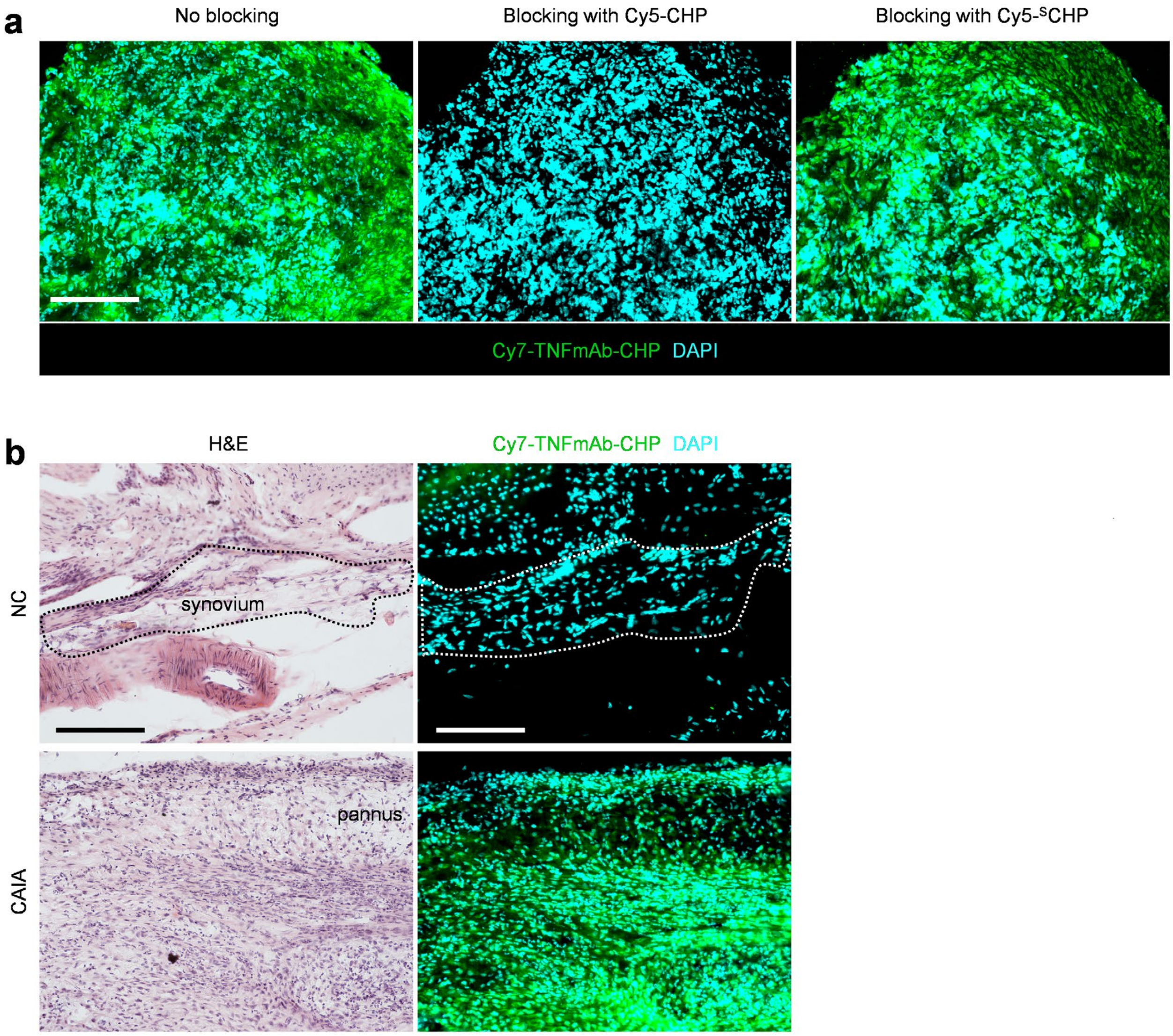

Supplementary Fig. 17. a Representative fluorescence micrographs of the cryosections from the ankle synovial pannus of CAIA mice, stained with Cy7-TNFmAb-CHP (200 μg/mL) overnight at 4 °C. Staining was performed with or without pre-blocking with either a high dose of Cy5-CHP or the sequence-scrambled control peptide Cy5-^{S}CHP (200 μM) overnight. **b** Representative fluorescence micrographs of cryosections of the normal mice's synovial tissue and the CAIA mice's pannus tissue stained with Cy7-TNFmAb-CHP. An adjacent section in each group was stained with H&E. Representative images from three independent experiments with similar results. Scale bars: 150 μm.

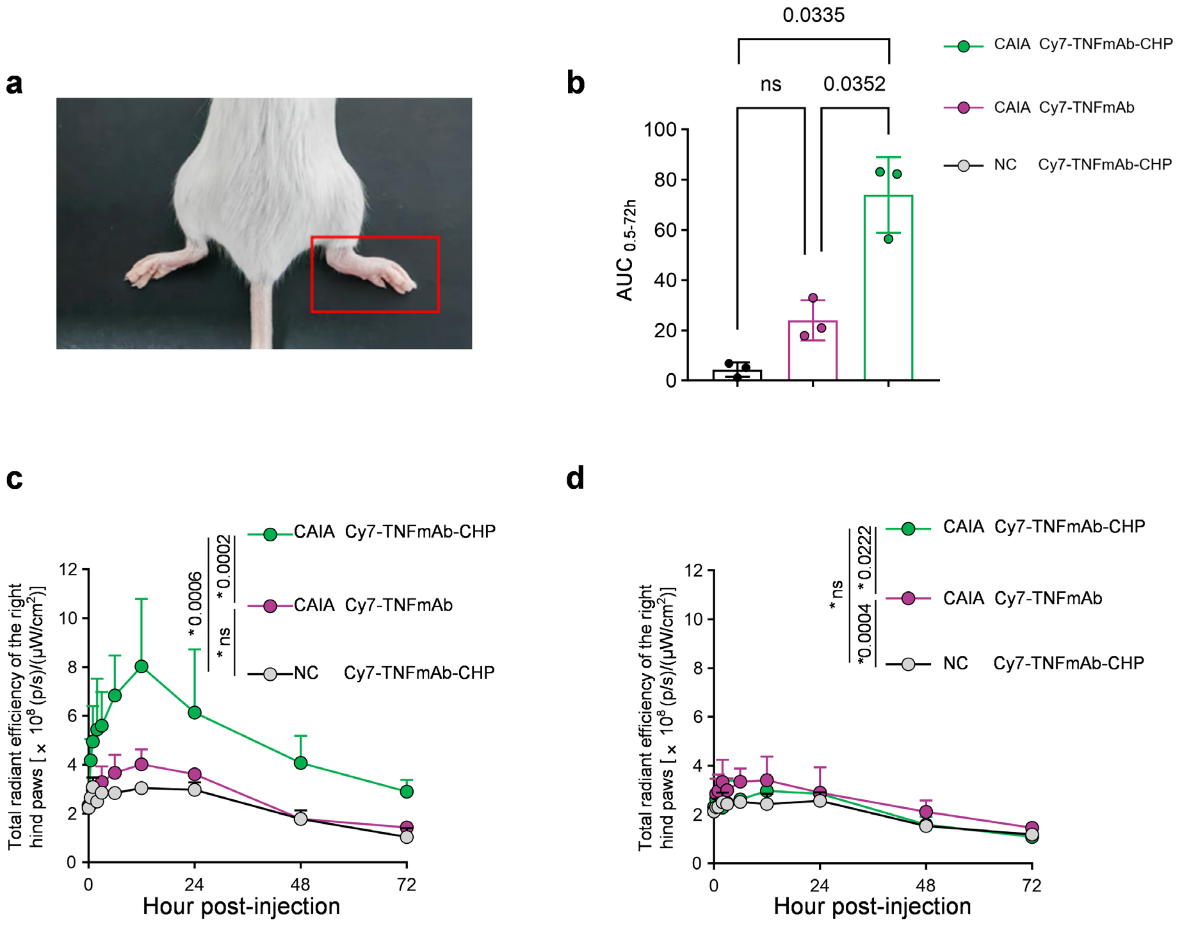

Supplementary Fig. 18. a Appearance of the inflamed arthritic right hind paw (red box) of a CAIA mouse triggered with local LPS injection (Fig. 3f). **b** Areas under the curve (AUC) of the radiant efficiency ratio of the arthritic paws (right hind) to the non-arthritic paws for Cy7-TNFmAb and Cy7-TNFmAb-CHP from 0.5 to 72 h post-injection in Fig. 3g (baseline ratio: 1.0). **c-d** Quantified in vivo Cy7 fluorescence signals from the right (**c**) and left (**d**) hind paws of the normal and CAIA mice studied in Fig. 3f from 0 to 72 h post-injection of Cy7-TNFmAb-CHP or Cy7-TNFmAb. Numbers were expressed as mean + standard deviation. Statistical analysis: Welch's ANOVA with post hoc Dunnett's T3 test (**b**), one-way repeated measures ANOVA with Geisser-Greenhouse correction for sphericity and Tukey's multiple comparisons (**c**, **d**).

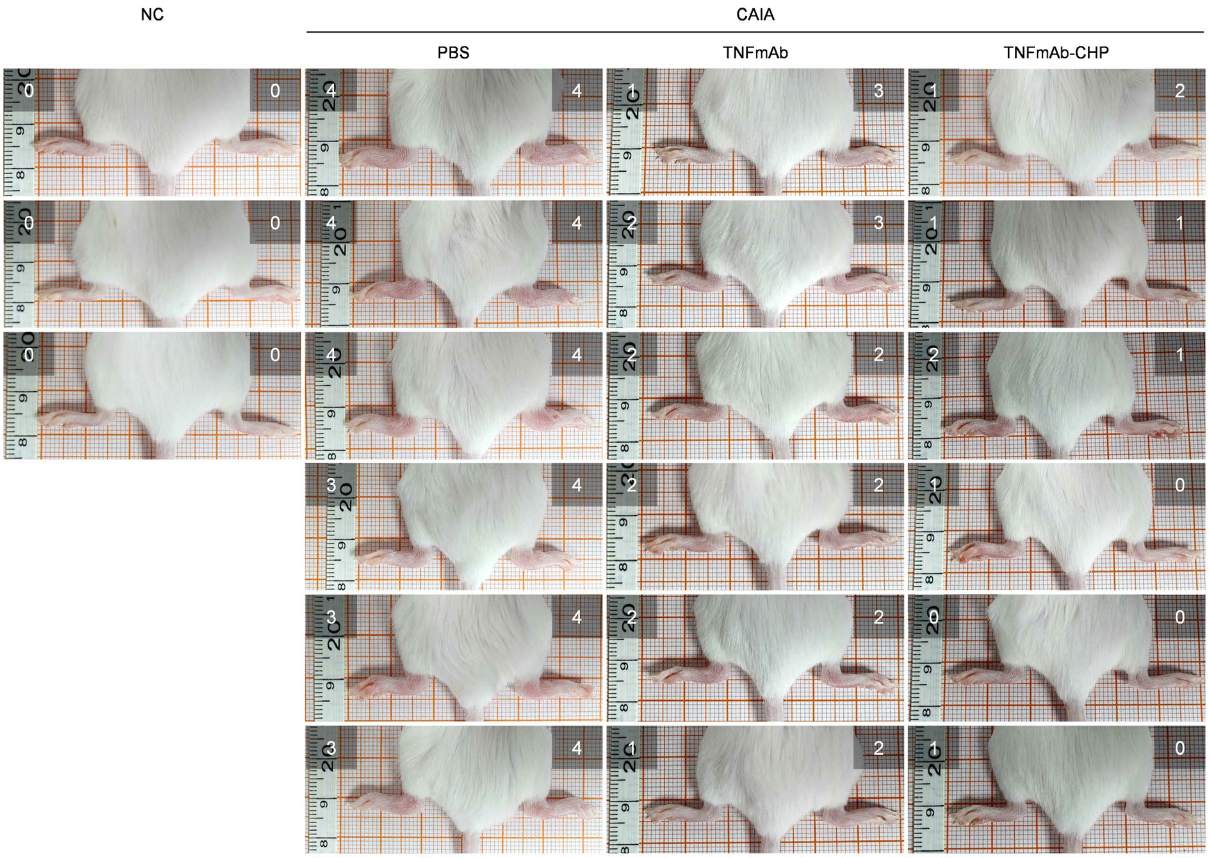

Supplementary Fig. 19. Photographs and arthritis scores of the hind paws of all NC and CAIA mice with different treatments.

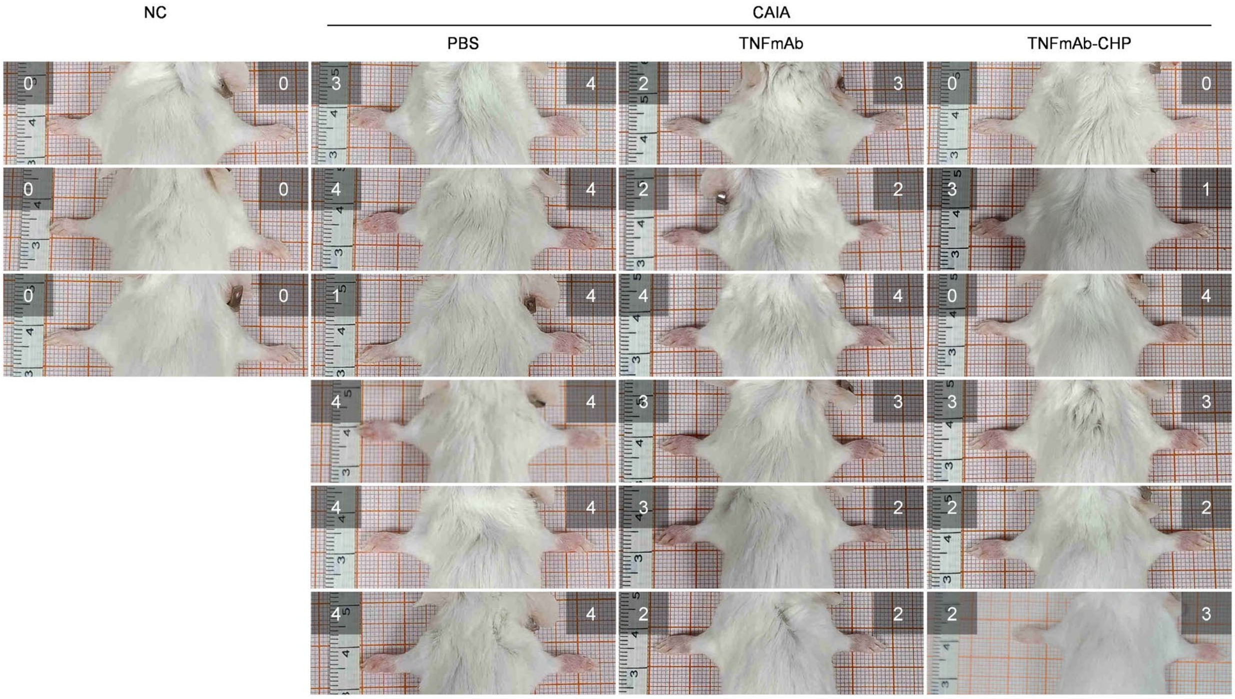

Supplementary Fig. 20. Photographs and arthritis scores of the front paws of all normal and CAIA mice with different treatments.

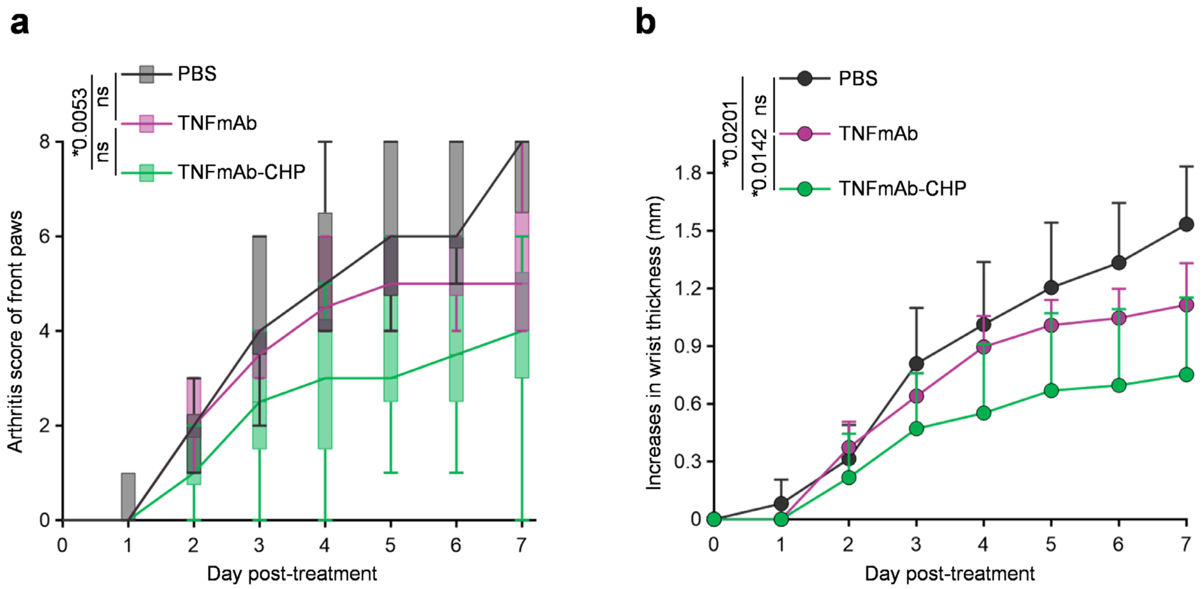

Supplementary Fig. 21. Combined arthritis scores (**a**) and increases in wrist thickness compared to day 0 (**b**) for the two front paws of the CAIA mice studied in Fig. 4a with different treatments. Numbers were expressed as median with interquartile range (**a**) and mean + standard deviation (**b**). Statistical analysis: Friedman test for repeated measures on ranks and Dunn's multiple comparisons (**a**), one-way repeated measures ANOVA with Geisser-Greenhouse correction for sphericity, and Tukey's multiple comparisons (**b**).

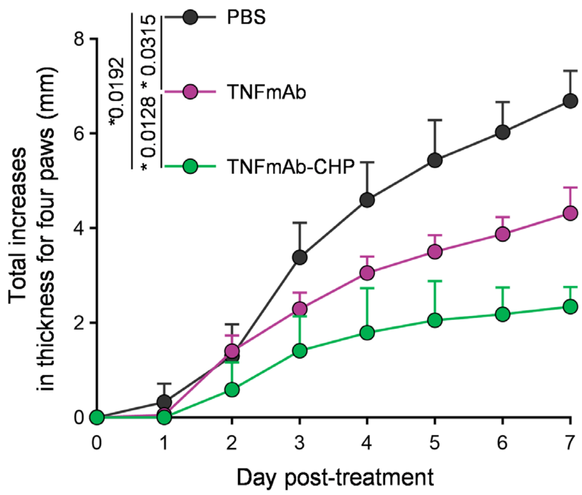

Supplementary Fig. 22. Total increases in thickness compared to day 0 for all four paws of the PBS, TNFmAb-, and TNFmAb-CHP-treated CAIA mice. Numbers were expressed as mean + standard deviation. Statistical analysis: one-way repeated measures ANOVA with Geisser-Greenhouse correction for sphericity and Tukey's multiple comparisons.

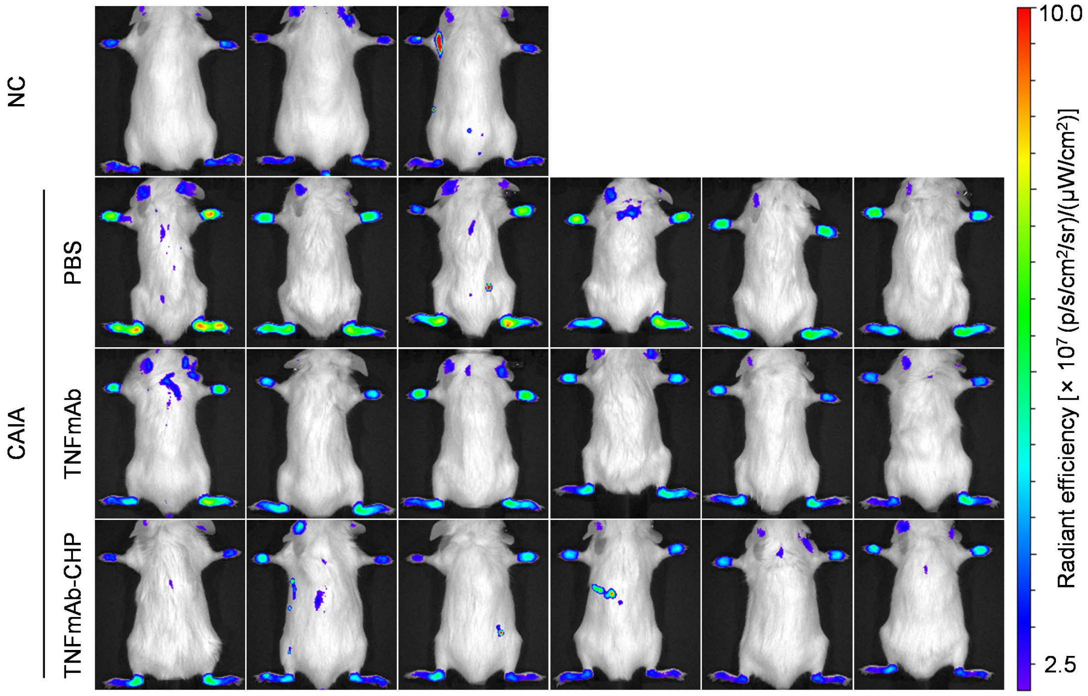

Supplementary Fig. 23. In vivo near-infrared fluorescence images of all the NC and the CAIA mice 7 days post different treatments. The mice were all intravenously injected with 1 nmole of Cy7-CHP 2 h before imaging.

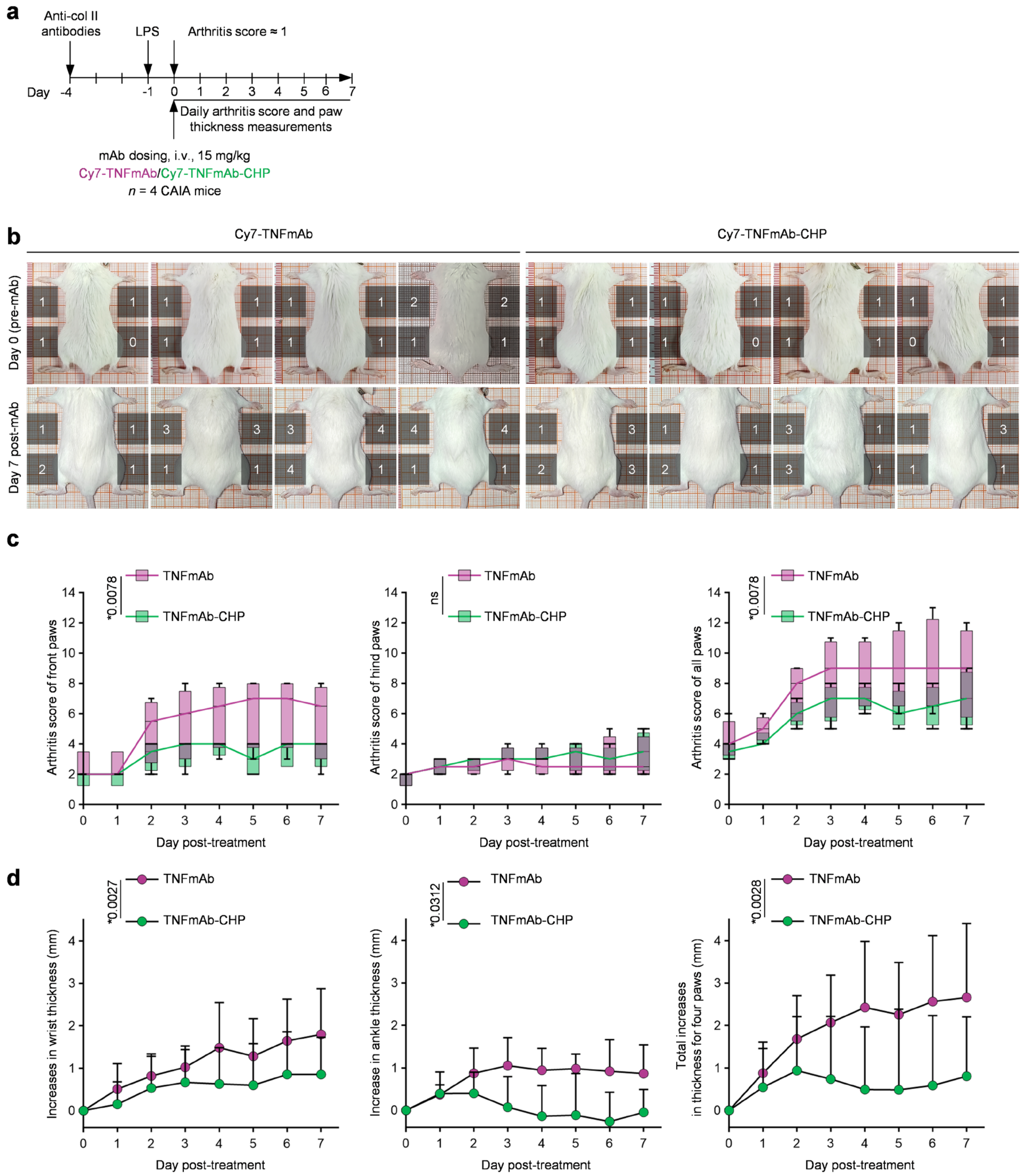

Supplementary Fig. 24. CHP-conjugation enhances the efficacy of TNFmAb in the CAIA mice (treatment start: average arthritis score per paw = 1). a Study timeline: each CAIA mouse (n = 4 mice) received a single i.v. dose of TNFmAb-CHP or TNFmAb (15 mg/kg) after the average arthritis score per paw reaches 1 (one day post-LPS injection). **b** Photographs and the arthritis score of each paw of the CAIA mice immediately before and 7 days after the treatments. **c** Combined arthritis scores for the front, hind, and all paws of the CAIA mice in the TNFmAb-CHP- and TNFmAb-treated groups. **d** Combined increases in ankle thickness compared to day 0 for the front, hind, and all paws of the CAIA mice in the TNFmAb-CHP- and TNFmAb-treated groups. Numbers were expressed as median with interquartile range (**c**) or mean with standard deviation (**d**). Statistical analysis: Wilcoxon matched-pairs signed rank test (**c**) and paired t-test (**d**).

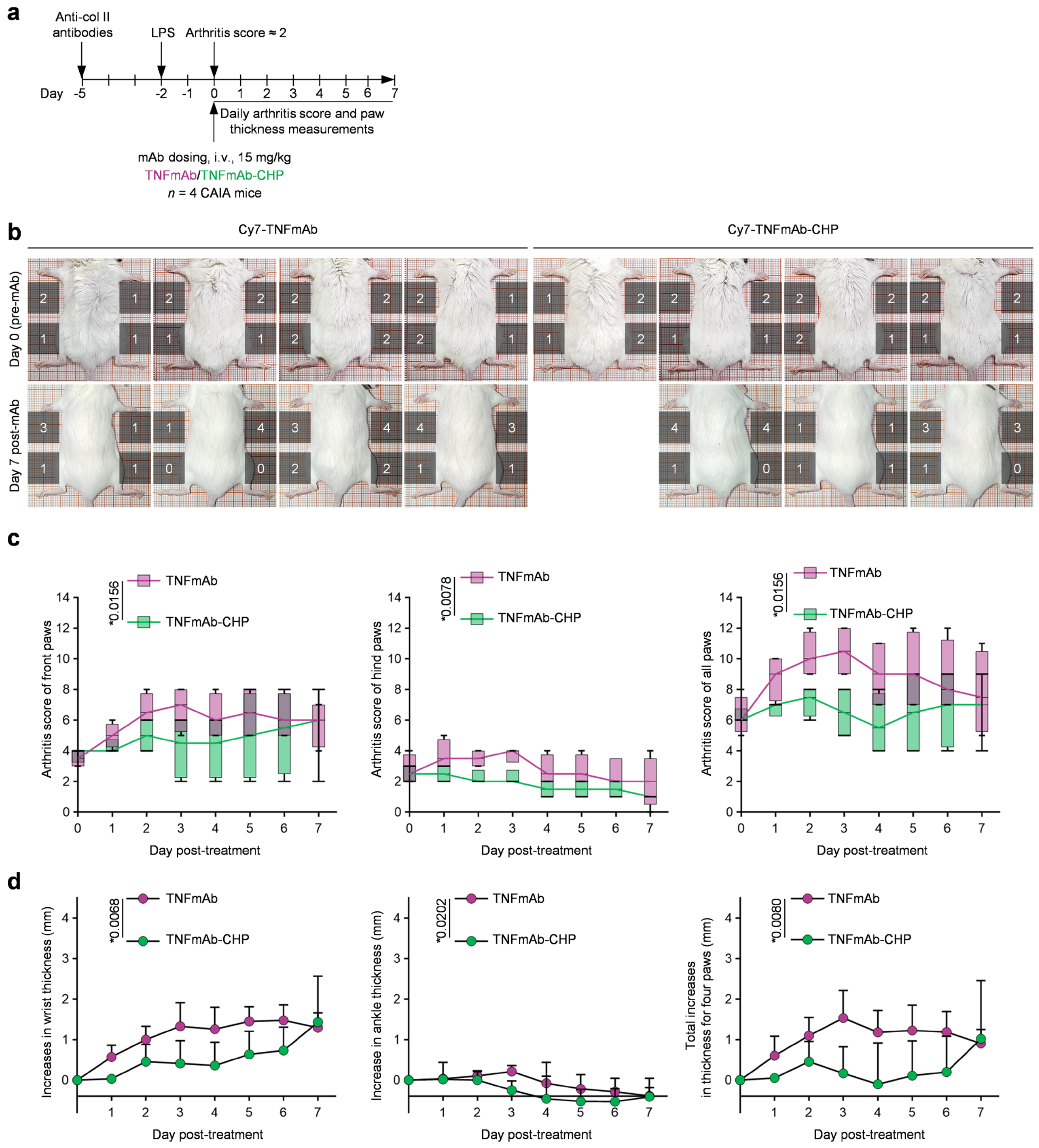

Supplementary Fig. 25. CHP-conjugation enhances the efficacy of TNFmAb in the CAIA mice (treatment start: average arthritis score per paw = 2). a Study timeline: each CAIA mouse (*n* = 4 mice) received a single i.v. dose of TNFmAb-CHP or TNFmAb (15 mg/kg) after the average arthritis score per paw reaches 2 (two days post-LPS injection). **b** Photographs and the arthritis score of each paw of the CAIA mice immediately before and 7 days after the treatments. **c** Combined arthritis scores for the front, hind, and all paws of the CAIA mice in the TNFmAb-CHP- and TNFmAb-treated groups. **d** Combined increases in ankle thickness compared to day 0 for the front, hind, and all paws of the CAIA mice in the TNFmAb-CHP- and TNFmAb-treated groups. Numbers were expressed as median with interquartile range (**c**) or mean with standard deviation (**d**). Statistical analysis: Wilcoxon matched-pairs signed rank test (**c**) and paired *t*-test (**d**).

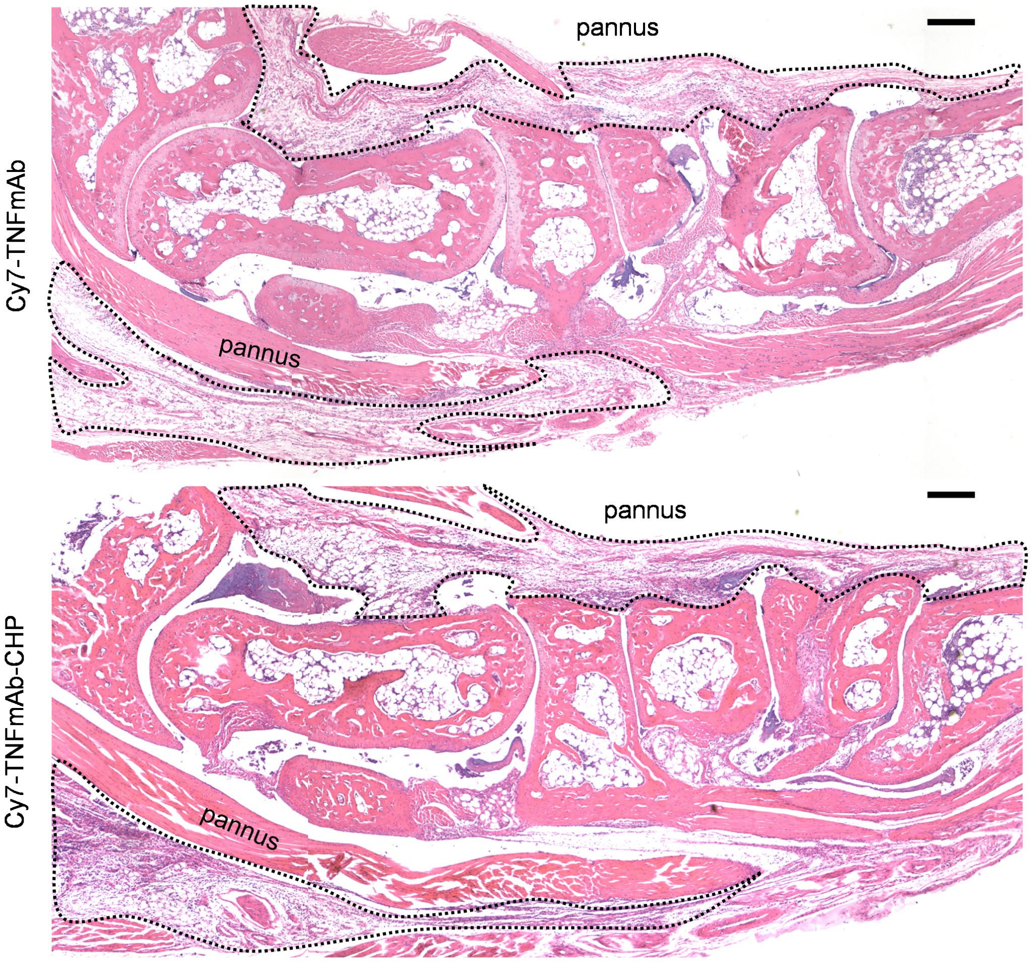

Supplementary Fig. 26. Representative H&E staining of paraffin-embedded sections of the ankle joints prepared from Fig. 5. Following light-sheet fluorescence imaging, the cleared tissues were rehydrated, processed for paraffin sectioning and stained with H&E. Scale bars: 100 μm.

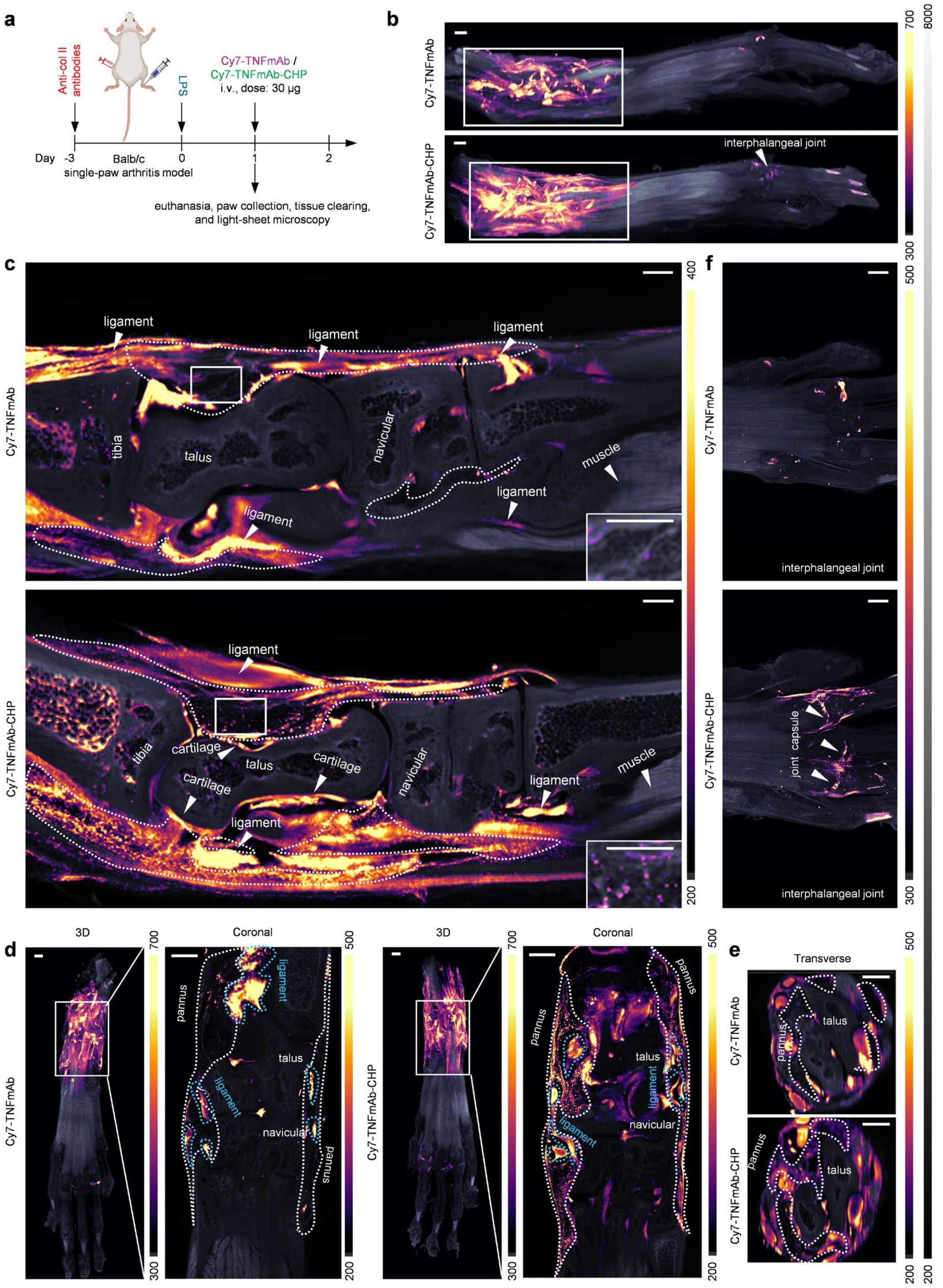

Supplementary Fig. 27. a Study timeline. Arthritis was selectively induced in the right hind paws of the CAIA mice by local LPS injection on day 0. The inflamed right hind paws were collected for tissue clearing 2 h post i.v. injection of Cy7-TNFmAb-CHP or Cy7-TNFmAb on day 1. **b-f** Representative light-sheet fluorescence microscopy images showing the distinct anatomical locations of Cy7-TNFmAb and Cy7-TNFmAb-CHP distribution within the arthritic hind paws (arthritis score: 1). 3D holistic view (**b**); 2D sagittal view (bottom right thumbnails: zoomed-in images for the boxed areas) (**c**); 3D and 2D coronal views (**d**); 2D transverse view (**e**); 3D coronal view of the toe digits (**f**). Pseudo-colors: mpl-inferno, Cy7 fluorescence; gray, autofluorescence. Representative images from three independent experiments with similar results (**b**, **c**, **d**, **e**, **f**). Scale bars: 250 µm (**c**), 500 µm (**b**, **d**, **e**, **f**).

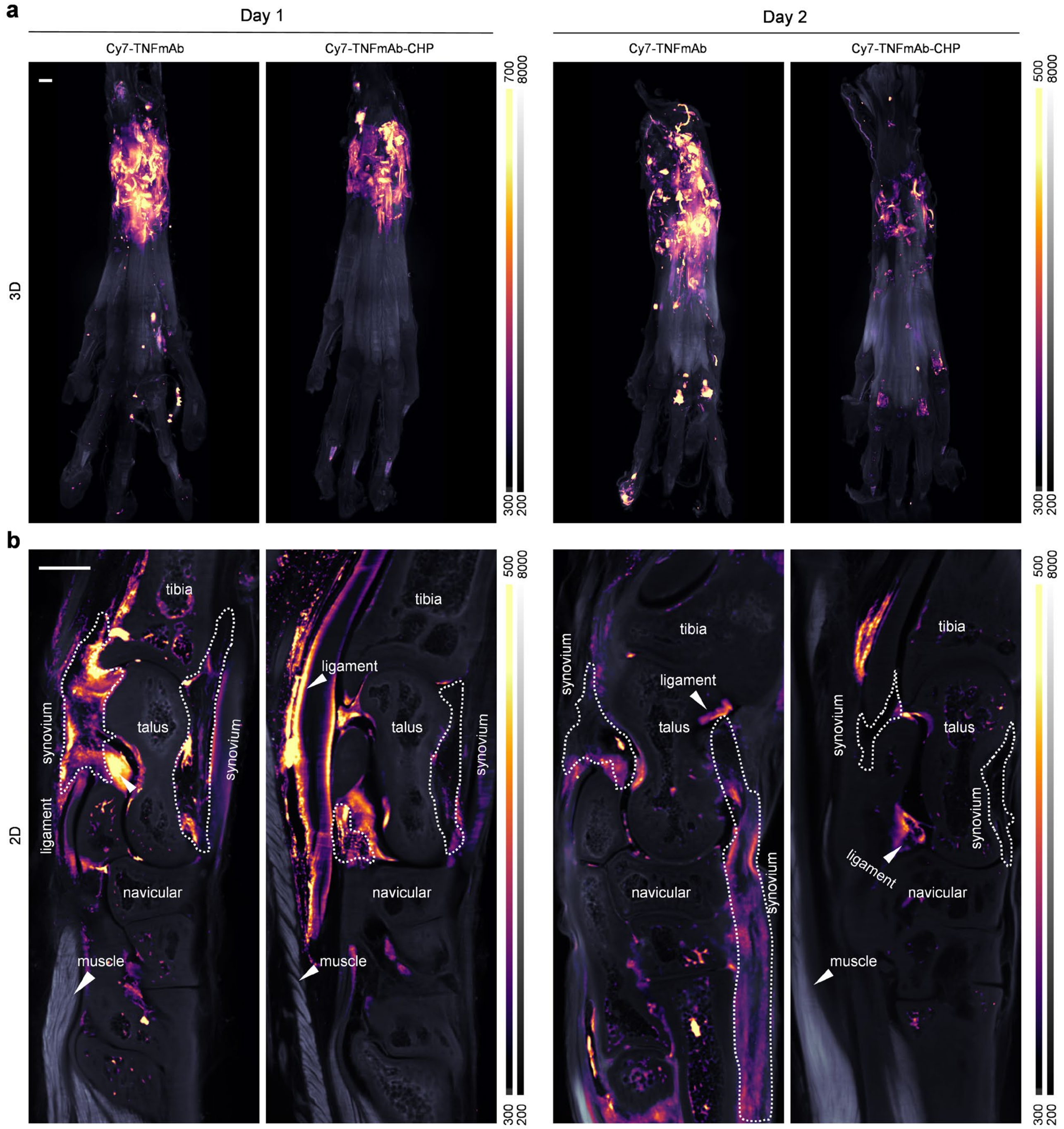

Supplementary Fig. 28. The 3D holistic views of the contralateral non-arthritic left hind paws from the CAIA mice collected 2 h post i.v. injection of Cy7-TNFmAb-CHP or Cy7-TNFmAb on day 1 or 2 post-LPS-injection in the right hind paws (shown in Supplementary Fig. 27a and Fig. 5a, respectively). 3D holistic view (**a**), 2D sagittal view (**b**). Representative images from three independent experiments with similar results. Scale bars: 500 μm.

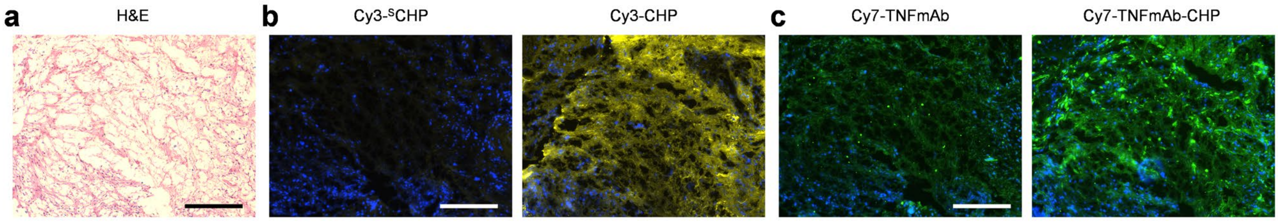

Supplementary Fig. 29. CHP recognizes and targets denatured collagen in the ankle pannus from collagen-induced arthritis (CIA) mice. **a** Representative H&E staining of the cryosections of the pannus tissues from the CIA mice. **b** Representative fluorescence micrographs showing binding of Cy3-CHP (but not the sequence-scrambled control peptide Cy3-^{S}CHP) to the cryosections of the pannus of the CIA mice (Cy3: yellow; DAPI: blue). **c** Representative fluorescence micrographs showing that Cy7-TNFmAb-CHP exhibits significantly stronger affinity to the CIA mice's synovial pannus than Cy7-TNFmAb (Cy7: green; DAPI: blue). Representative images from three independent experiments with similar results. Scale bars: 150 μm (**a**, **b**, **c**).

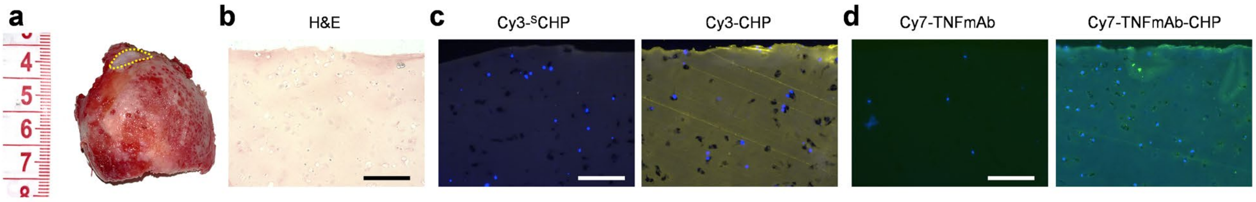

Supplementary Fig. 30. CHP and TNFmAb-CHP attach to denatured collagen in the damaged cartilage from an RA patient. a Photograph of an articular cartilage specimen surgically removed from an RA patient during shoulder arthroplasty. The remaining arthritic cartilage within the yellow-dotted region was cryosectioned. **b** Representative H&E stain. **c** Representative fluorescence micrographs showing the binding of Cy3-CHP (but not the sequence-scrambled control peptide Cy3-^{S}CHP) to the RA cartilage (Cy3: yellow; DAPI: blue). **d** Representative fluorescence micrographs showing that Cy7-TNFmAb-CHP exhibits significantly stronger affinity to the RA cartilage than Cy7-TNFmAb (Cy7: green; DAPI: blue). Representative images from three independent experiments with similar results. Scale bars: 150 µm (**b**, **c**, **d**).

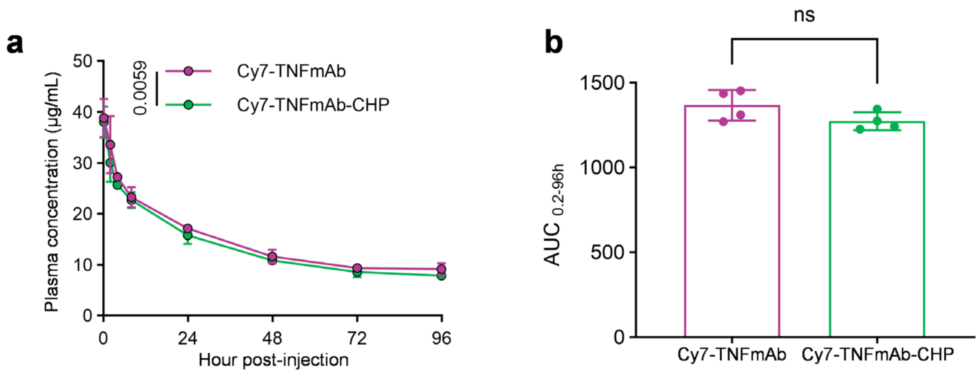

Supplementary Fig. 31. a Fluorescence based quantification of Cy7-TNFmAb and Cy7-TNFmAb-CHP concentrations in the plasma of NC mice at different time points post i.v. injection (dose: 30 μg). **b** AUC of the plasma concentration-time curves for Cy7-TNFmAb and Cy7-TNFmAb-CHP from 0.2 to 96 h post-injection. Statistical analysis: paired *t*-test (**a**) and unpaired *t*-test (**b**).

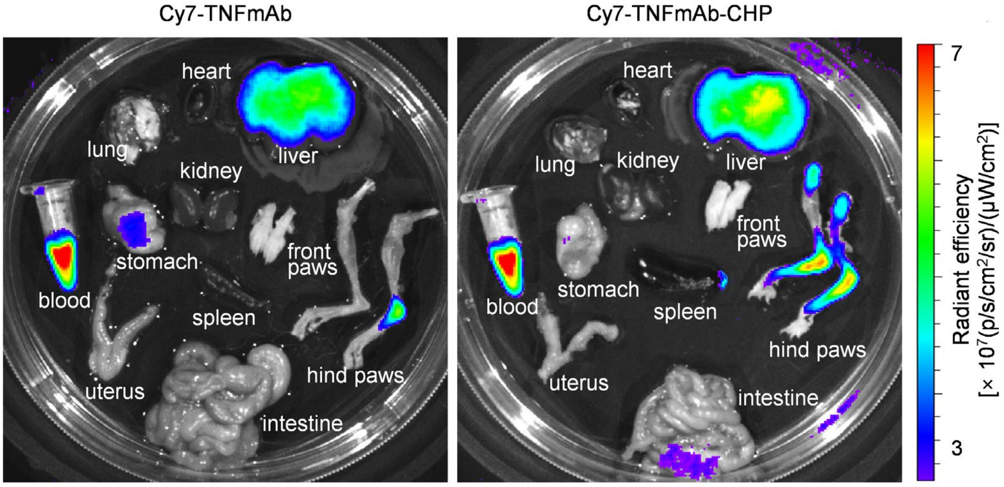

Supplementary Fig. 32. Representative fluorescence images of the major organs, paws, and blood of CAIA mice harvested 2 h post i.v. injection of 30 μg of Cy7-TNFmAb or Cy7-TNFmAb-CHP.

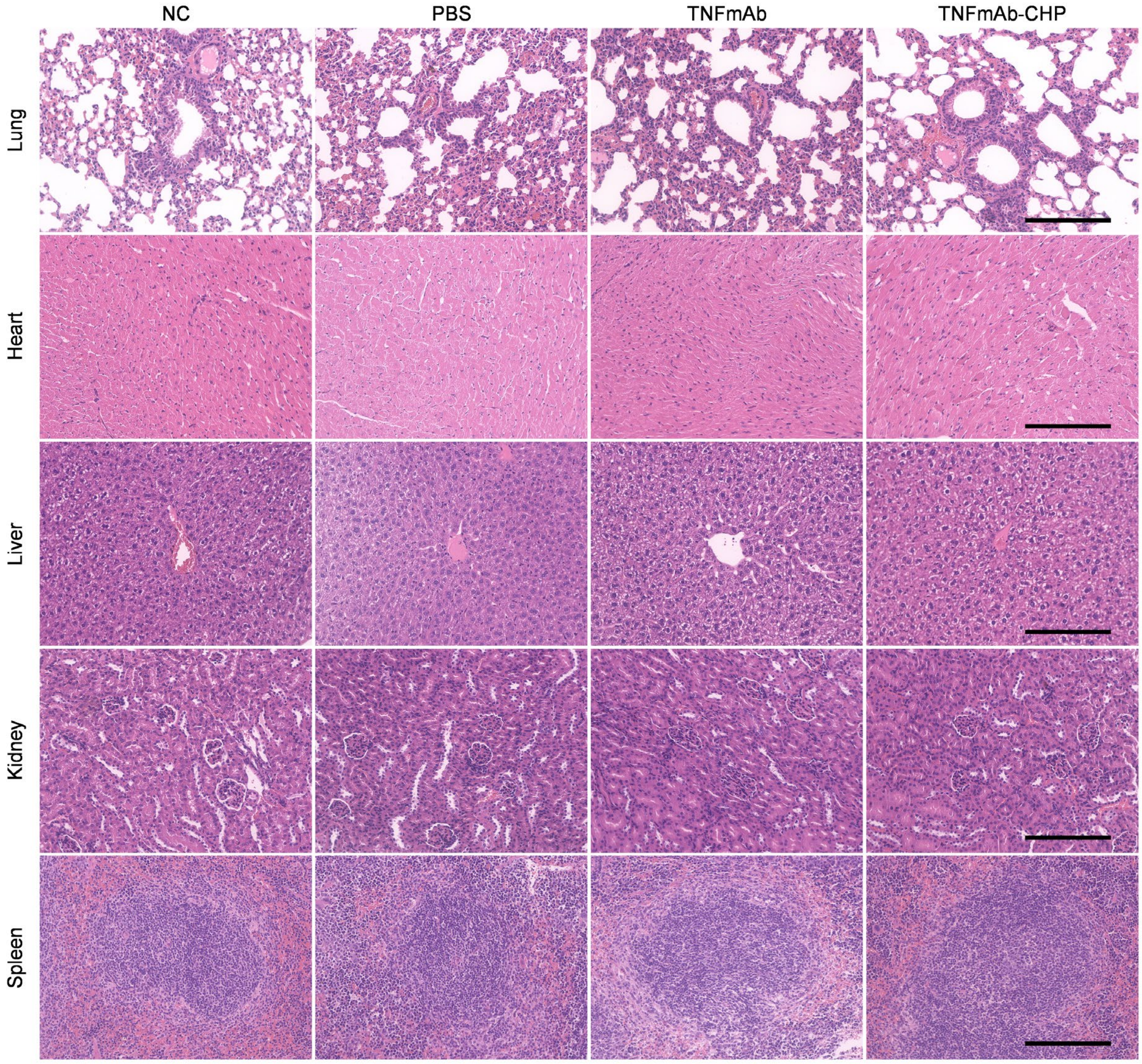

Supplementary Fig. 33. Representative micrographs of the H&E-stained paraffin-embedded tissue sections of the lungs, hearts, livers, kidneys, and spleens from all the NC or CAIA mice collected at the endpoint of the study described in Fig. 4a. Representative images from three independent experiments with similar results. Scale bars: 200 μm.

Supplementary Table 1. Designs of all mouse studies

Study	Grouping	Group size (*n*)	Aim	Tests	Data in Fig.
A	NC (Cy7-CHP), CAIA (Cy7-CHP), CAIA (Cy7-^{S}CHP)	3	Visualizing collagen breakdown in RA joints fluorescently; checking the degree of bone erosion in normal and inflamed paws by CT	In vivo and ex vivo Cy7-CHP fluorescence imaging, Micro-CT scanning	Fig. 1a, Supplementary Fig. 1, Supplementary Fig. 3
B	NC (Cy3-CHP), CAIA (Cy3-CHP), CAIA (Cy3-^{S}CHP)	3	Histological localization of collagen denaturation in joints	Ex vivo CHP staining and fluorescence imaging, Safranin O staining, immunostaining for MMPs, CTSK, and collagens	Figs. 1c–f, Supplementary Fig. 6–10
C	NC, CAIA [CHP: Cy7-Ahx-K-Ahx-(GfO)$_9$]	2	Three-dimensional spatial distribution of denatured collagen in hind paws	In vivo CHP injection, tissue clearing, and light-sheet fluorescence microscopy	Fig. 1b, Supplementary Movies 1&2
D	CAIA (Cy7-CHP) CAIA (Cy7-^{S}CHP)	4	Correlation between arthritis score and in vivo CHP uptake	In vivo CHP fluorescence imaging	Figs. 2a–e, Supplementary Figs. 11, 12
E	NC (Cy7-CHP), CAIA (PBS-treated, Cy7-CHP), CAIA (DEX-treated, Cy7-CHP)	5 per CAIA group, 3 for the NC group	Detecting or live monitoring changes in collagen destruction in inflamed ankles	In vivo CHP injection, ex vivo fluorescence imaging, histology with H&E and Safranin O staining	Figs. 2f–k, Supplementary Fig. 13
F	CAIA (Cy7-CHP)	5		In vivo CHP fluorescence imaging	Figs. 2l–n, Supplementary Fig. 14
G	NC (Cy7-TNFmAb-CHP), CAIA (Cy7-TNFmAb), CAIA (Cy7-TNFmAb-CHP)	3	Comparison of the accumulation of Cy7-labeled TNFmAb-CHP and TNFmAb in arthritic paws	In vivo TNFmAb injection, in vivo fluorescence imaging	Figs. 3f–g, Supplementary Fig. 18
H	NC, CAIA (PBS), CAIA (TNFmAb), CAIA (TNFmAb-CHP)	6 per CAIA group, 3 for the NC group	Efficacy test of the TNFmAbs in CAIA mice	In vivo CHP injection, in vivo fluorescence imaging, histology, H&E and Safranin O staining, histological biosafety assessment	Fig. 4, Supplementary Figs.19–23
I	NC (Cy7-CHP), CAIA (Cy7-CHP)	3	In vivo clearance of Cy7-CHP	In vivo fluorescence imaging	Supplementary Fig. 2
J	CAIA (Cy7-TNFmAb), CAIA (Cy7-TNFmAb-CHP)	2	Tracing the in vivo distribution of Cy7-TNFmAb-CHP in the arthritic paws.	Light-sheet fluorescence microscopy	Fig. 5, Supplementary Figs. 27, 28
K	CAIA (Cy7-TNFmAb), CAIA (Cy7-TNFmAb-CHP)	4	Pharmacokinetics of Cy7-TNFmAb and Cy7-TNFmAb-CHP	Fluorescence plasma imaging and measurement	Figs. 6a–d
L	CAIA (Cy7-TNFmAb), CAIA (Cy7-TNFmAb-CHP)	3	Biodistribution of Cy7-TNFmAb and Cy7-TNFmAb-CHP	Ex vivo fluorescence imaging	Figs. 6e, f
M	CAIA (Cy7-CHP), CAIA (Lys-modified-Cy7-CHP)	3	In vivo targeting, pharmacokinetics of Cy7-CHP and Lys-modified-Cy7-CHP,	In vivo and ex vivo fluorescence imaging, fluorescence plasma measurement	Supplementary Figs. 5b, c
N	CAIA (Cy7-CHP), CAIA (Lys-modified-Cy7-CHP)	2	Biodistribution of Cy7-CHP and Lys-modified-Cy7-CHP	Ex vivo fluorescence imaging	Supplementary Fig. 5d
P	CAIA (TNFmAb), CAIA (TNFmAb-CHP)	4	Efficacy tests of TNFmAb and TNFmAb-CHP in CAIA mice (arthritis scores: 1 or 2)	Arthritis score and paw thickness measurement	Supplementary Figs. 24 & 25

Supplementary Table 2. Fluorescently-labeled peptides used in this study and their MALDI MS information

Peptide	Sequence*	m/z calculated		m/z found
Cy3-CHP	Cy3-Ahx-GPOGPOGPOGPOGPOGPOGPOGPOGPO	$[M + H^+]$	3133.4	3134.0
Cy3-^{S}CHP	Cy3-Ahx-PGOGPGPOPOGOGOPPGOOPGGOOPPG	$[M + H^+]$	3133.3	3132.4
Cy7-CHP	Cy7-Ahx-GfOGfOGfOGfOGfOGfOGfOGfOGfO	$[M + Na^+]$	3409.37	3409.36
Cy7-^{S}CHP	Cy7-Ahx-OfGGOfGfGfOfOGOfGOOfGGOOffG	$[M + Na^+]$	3409.37	3409.91
Cy7-Ahx-K-Ahx-CHP	Cy7-Ahx-K-Ahx-fOGfOGfOGfOGfOGfOGfOGfOGfO	$[M + H^+]$	3628.5	3628.91
K(N_3)-CHP	NH_2-K(N_3)-Ahx-GfOGfOGfOGfOGfOGfOGfOGfOGfO	$[M + Na^+]$	2873.21	2873.35
Cy7-K(N_3)-CHP	Cy7-K(N_3)-Ahx-GfOGfOGfOGfOGfOGfOGfOGfOGfO	$[M + H^+]$	3540.45	3540.38

*Ahx: aminohexanoic acid, O: (2S,4R)-4-hydroxyproline, f: (2S,4S)-4-fluoro-proline, Cy3: sulfo-cyanine 3, Cy7: sulfo-cyanine 7, K(N_3): azidolysine.
