# Supplementary material for: Collagen hybridizing peptide imaging and delivery of therapeutic antibody in rheumatoid arthritis models
Source: Nat Commun. 2026 Apr 20;17:5423. doi: 10.1038/s41467-026-72038-y (PMC13279783; doi:10.1038/s41467-026-72038-y)
Supplement: Supplementary file 3 — Reporting Summary [file 41467_2026_72038_MOESM3_ESM.pdf]

nature portfolio

Corresponding author(s): Yang Li

Last updated by author(s): Mar 23, 2026

Reporting Summary

Nature Portfolio wishes to improve the reproducibility of the work that we publish. This form provides structure for consistency and transparency in reporting. For further information on Nature Portfolio policies, see our Editorial Policies and the Editorial Policy Checklist.

Statistics

For all statistical analyses, confirm that the following items are present in the figure legend, table legend, main text, or Methods section.

n/a	Confirmed	
☐	☒	The exact sample size (n) for each experimental group/condition, given as a discrete number and unit of measurement
☐	☒	A statement on whether measurements were taken from distinct samples or whether the same sample was measured repeatedly
☐	☒	The statistical test(s) used AND whether they are one- or two-sided<br>*Only common tests should be described solely by name; describe more complex techniques in the Methods section.*
☒	☐	A description of all covariates tested
☐	☒	A description of any assumptions or corrections, such as tests of normality and adjustment for multiple comparisons
☐	☒	A full description of the statistical parameters including central tendency (e.g. means) or other basic estimates (e.g. regression coefficient) AND variation (e.g. standard deviation) or associated estimates of uncertainty (e.g. confidence intervals)
☐	☒	For null hypothesis testing, the test statistic (e.g. F, t, r) with confidence intervals, effect sizes, degrees of freedom and P value noted<br>*Give P values as exact values whenever suitable.*
☒	☐	For Bayesian analysis, information on the choice of priors and Markov chain Monte Carlo settings
☒	☐	For hierarchical and complex designs, identification of the appropriate level for tests and full reporting of outcomes
☐	☒	Estimates of effect sizes (e.g. Cohen's d, Pearson's r), indicating how they were calculated

Our web collection on statistics for biologists contains articles on many of the points above.

Software and code

Policy information about availability of computer code

Data collection	H&E and fluorescence micrographs were acquired using an EVOS M7000 imaging system Software (Version 2.1.677.717). Light sheet microscopy fluorescence images were acquired using an ImSpector Main Application (Version 7.6.3). Mass spectra were obtained on a Shimadzu 8020 matrix-assisted laser desorption/ionization–time of flight (MALDI-TOF) mass spectrometer (ICS version 2.7.0.1245).
Data analysis	Light sheet fluorescence microscopy images were reconstructed with the Imaris software (Version 9.8.0, Oxford Instruments). In vivo fluorescence images were analyzed using Living Image Software (Version 4.5.5). All data in this study were analyzed and plotted using the Graphpad Prism software (version 10.0.0, Dotmatics).

For manuscripts utilizing custom algorithms or software that are central to the research but not yet described in published literature, software must be made available to editors and reviewers. We strongly encourage code deposition in a community repository (e.g. GitHub). See the Nature Portfolio guidelines for submitting code & software for further information.

Data

Policy information about availability of data

All manuscripts must include a data availability statement. This statement should provide the following information, where applicable:

- Accession codes, unique identifiers, or web links for publicly available datasets
- A description of any restrictions on data availability
- For clinical datasets or third party data, please ensure that the statement adheres to our policy

The main data supporting the results of this study are available within the paper and its supplementary information. All raw data generated in this study are

provided in the source data file.

Research involving human participants, their data, or biological material

Policy information about studies with human participants or human data. See also policy information about sex, gender (identity/presentation), and sexual orientation and race, ethnicity and racism.

Reporting on sex and gender	This study utilized joint samples from a female patient with Rheumatoid Arthritis.
Reporting on race, ethnicity, or other socially relevant groupings	This information was not specified for the patient in this study.
Population characteristics	The study utilized an articular cartilage specimen from a female patient with rheumatoid arthritis, obtained during shoulder arthroplasty surgery.
Recruitment	The articular cartilage specimen was surgically removed from an RA patient during a planned shoulder arthroplasty procedure at The Fifth Affiliated Hospital of Sun Yat-sen University.
Ethics oversight	All experimental plans involving histological analysis of joint specimens from RA patients were approved by the Ethics Committee of The Fifth Affiliated Hospital of Sun Yat-sen University (Approval Number: K150-1). The specimen was obtained in accordance with ethical standards.

Note that full information on the approval of the study protocol must also be provided in the manuscript.

Field-specific reporting

Please select the one below that is the best fit for your research. If you are not sure, read the appropriate sections before making your selection.

☒ Life sciences ☐ Behavioural & social sciences ☐ Ecological, evolutionary & environmental sciences

For a reference copy of the document with all sections, see nature.com/documents/nr-reporting-summary-flat.pdf

Life sciences study design

All studies must disclose on these points even when the disclosure is negative.

Sample size	The sample sizes for all in vivo mouse experiments were chosen based on established standards and conventions in the field for preliminary proof-of-concept and efficacy studies. A minimum group size of n ≥ 3 was used for all quantitative analyses to account for biological variability and to allow for meaningful statistical comparison.
Data exclusions	No data were excluded from the analyses.
Replication	All experiments involving were performed at least three independent times, with each independent experiment containing multiple technical replicates to ensure consistency.
Randomization	All allocation was random.
Blinding	Blinding was implemented for all subjective assessments in this study. Specifically, H&E-stained slides were scored by pathologists who were blinded to the experimental groups (Fig. 2i–k). All clinical arthritis scoring and paw measurements were also conducted in a blinded manner, with the investigator responsible for the scoring kept unaware of treatment allocations throughout the experiment (Figs. 2 and 4b).<br>For experiments involving automated data acquisition, including IVIS imaging, EVOS imaging, and microplate reader measurements, blinding was not performed, as data were acquired automatically by the instruments, providing objective measurements not susceptible to observer bias.

Reporting for specific materials, systems and methods

We require information from authors about some types of materials, experimental systems and methods used in many studies. Here, indicate whether each material, system or method listed is relevant to your study. If you are not sure if a list item applies to your research, read the appropriate section before selecting a response.

Materials & experimental systems

n/a	Involved in the study	
	x	Antibodies
x		Eukaryotic cell lines
x		Palaeontology and archaeology
	x	Animals and other organisms
x		Clinical data
x		Dual use research of concern
x		Plants

Methods

n/a	Involved in the study	
x		ChIP-seq
x		Flow cytometry
x		MRI-based neuroimaging

Antibodies

Antibodies used

1. Anti-MMP1 antibody: rabbit polyclonal to MMP1, Proteintech, catalog number: 10371-2-AP.
2. Anti-MMP2 antibody: rabbit polyclonal to MMP2, GeneTex, catalog number: GTX104577.
3. Anti-MMP3 antibody: rabbit polyclonal to MMP3, Proteintech, catalog number: 17873-1-AP.
4. Anti-MMP8 antibody: rabbit polyclonal to MMP8, Proteintech, catalog number: 17874-1-AP.
5. Anti-MMP9 antibody: rabbit polyclonal to MMP9, Abcam, catalog number: ab38898.
6. Anti-MMP13 antibody: rabbit polyclonal to MMP13, Proteintech, catalog number: 18165-1-AP.
7. Anti-MMP14 antibody: rabbit polyclonal to MMP14, Abcam, catalog number: ab51074, clone: EP1264Y.
8. Anti-CTSK antibody: mouse monoclonal to CTSK, Santa Cruz Biotechnology, catalog number: sc-48353, clone: E-7.
9. Anti-Collagen I antibody: rabbit polyclonal to collagen I, Abcam, catalog number: ab34710, lot number: GR3432109-2.
10. Anti-Collagen IV antibody: rabbit polyclonal to collagen IV, Abcam, catalog number: ab6586.
11. Anti-Collagen Type I 3/4 fragment antibody: rabbit polyclonal, Adipogen Life Sciences, catalog number: AG-25T-0116-C025.
12. Anti-TNF-α monoclonal antibody: Rat monoclonal to mouse TNF-α, Bio X Cell, catalog number: BE0058, clone XT3.11.

Validation

1. Anti-MMP1 antibody 10371-2-AP (Proteintech) KO Validated. This rabbit polyclonal antibody has been KD/KO validated and is suitable for FC, IF, IHC, IP, WB, and ELISA . It reacts with human, mouse, and rat.
https://www.ptglab.com/products/MMP1-Antibody-10371-2-AP.htm

2. Anti-MMP2 antibody GTX104577 (GeneTex) Validated by multiple applications. This rabbit polyclonal antibody has been validated for WB, ICC/IF, IHC-P, IHC-Fr, and IP . It reacts with human, mouse, rat, and pig.
https://www.genetex.com/Product/Detail/MMP2-antibody/GTX104577

3. Anti-MMP3 antibody 17873-1-AP (Proteintech) Validated by broad application testing. This rabbit polyclonal antibody has been validated for WB, IHC (Paraffin), ICC/IF, and Flow Cytometry . It reacts with human, mouse, and rat.
https://www.ptglab.com/products/MMP3-Antibody-17873-1-AP.htm

4. Anti-MMP8 antibody 17874-1-AP (Proteintech) Validated by multiple applications. This rabbit polyclonal antibody has been validated for IHC, IP, WB, and ELISA . It reacts with human, mouse, and rat . Validation includes western blot on HepG2 cells and IHC on human placenta.
https://www.ptglab.com/products/MMP8-Antibody-17874-1-AP.htm

5. Anti-MMP9 antibody ab38898 (Abcam) Widely published & specific. This rabbit polyclonal antibody has been cited in over 1,030 publications . It is validated for WB and reacts with mouse.
https://www.abcam.com/en-us/products/primary-antibodies/mmp9-antibody-ab38898

6. Anti-MMP13 antibody 18165-1-AP (Proteintech) Validated by multiple applications and independent testing. This rabbit polyclonal antibody has been validated for WB, IP, IHC, IF, and ELISA . Independent validation by NYU Langone was performed for IHC. The RRID is AB_2144858.
https://www.ptglab.com/products/MMP13-Antibody-18165-1-AP.htm

7. Anti-MMP14 antibody ab51074 (Abcam) KO Validated & Recombinant. This rabbit monoclonal antibody [EP1264Y] is the most cited clone for MMP14 . It has been validated in knockout (KO) models and is suitable for WB, Flow Cytometry, IP, IHC-P, and ICC/IF. It reacts with human, mouse, and rat.
https://www.abcam.com/en-us/products/primary-antibodies/mmp14-antibody-ep1264y-ab51074

8. Anti-CTSK antibody sc-48353 (Santa Cruz) Validated by peer-reviewed publications. This mouse monoclonal antibody [E-7] has been cited in 165 publications . It detects cathepsin K in mouse, rat, and human samples . Validated applications include WB, IP, IF, IHC(P), and ELISA.
https://www.scbt.com/p/cathepsin-k-antibody-e-7

9. Anti-Collagen I antibody ab34710 (Abcam) Widely published. This rabbit polyclonal antibody has been cited in over 1,850 publications . It is suitable for IHC-P, WB, and ICC/IF.
https://www.abcam.com/en-us/products/primary-antibodies/collagen-i-collagen-iii-antibody-ab34710

10. Anti-Collagen IV antibody ab6586 (Abcam) Widely published & multi-species validated. This rabbit polyclonal antibody has been cited in over 920 publications . It reacts with human, mouse, rat, cow, pig, zebrafish, and more . Validated applications include IHC-P, IHC-Fr, WB, IP, ICC/IF, and ELISA . It has negligible cross-reactivity with Collagen types I, II, III, V, or VI.
https://www.abcam.com/en-us/products/primary-antibodies/collagen-iv-antibody-ab6586

11. Anti-Collagen Type I 3/4 fragment antibody AG-25T-0116-C025 (Adipogen Life Sciences) Specific for the cleaved fragment. This rabbit polyclonal antibody is designed to specifically recognize the C-terminal end of the N-terminal three-quarter collagen fragment (Col1 ¾) generated by MMP cleavage . It reacts with rat and bovine Collagen I, and is predicted to react with human, mouse, and other species based on sequence identity . Validated applications include ICC and WB.
https://adipogen.com/ag-25t-0116-anti-collagen-type-1-3-4-fragment-pab.html

12. Anti-TNF-α monoclonal antibody (for CHP conjugation): Rat monoclonal to mouse TNF-α (clone XT3.11), Bio X Cell, catalog number: BE0058. It is validated for use in in vitro and in vivo TNF-α neutralization assays, as well as Western blotting.
Application reference 1: Shaabani, N., et al (2018). "The probacterial effect of type I interferon signaling requires its own negative regulator USP18" Sci Immunol 3(27).
Application reference 2:Baeyens, A., et al (2015). "Effector T cells boost regulatory T cell expansion by IL-2, TNF, OX40, and plasmacytoid dendritic cells depending on the immune context" J Immunol 194(3): 999-1010.
Application reference 3:Christensen, A. D., et al (2015). "Depletion of regulatory T cells in a hapten-induced inflammation model results in prolonged and increased inflammation driven by T cells" Clin Exp Immunol 179(3): 485-499.
Application reference 4:Grinberg-Bleyer, Y., et al (2015). "Cutting edge: NF-kappaB p65 and c-Rel control epidermal development and immune homeostasis in the skin" J Immunol 194(6): 2472-2476.
https://bioxcell.com/invivomab-anti-mouse-tnf-alpha-be0058

Animals and other research organisms

Policy information about studies involving animals; ARRIVE guidelines recommended for reporting animal research, and Sex and Gender in Research

Laboratory animals	Female Balb/c mice (7 to 8 weeks old) were purchased from Zhiyuan (Guangdong) Biomedical Technology Co., Ltd. (stock number: XW20240620-01) and co-housed in a specific-pathogen-free (SPF) grade facility with a 12-hour cycle of light and darkness at a controlled temperature of 22 ± 2 °C and humidity of 50 ± 10%, with free access to food and water.
Wild animals	The study did not involve wild animals.
Reporting on sex	Findings apply to one sex only. This study utilized exclusively female BALB/c mice (8–12 weeks old). The decision was based on clinical relevance (RA prevalence is significantly higher in women) and model reliability (female mice exhibit more consistent susceptibility in the CAIA model).
Field-collected samples	This study does not involve field-collected samples.
Ethics oversight	Experimental animal use and ethics committee of the Fifth Affiliated Hospital of Sun Yat-sen University.

Note that full information on the approval of the study protocol must also be provided in the manuscript.

Plants

Seed stocks	This study did not involve any plant materials, seed stocks, or specimens collected from the field.
Novel plant genotypes	This study did not involve the generation or analysis of novel plant genotypes, including those produced by transgenic approaches, gene editing, mutagenesis, or hybridization.
Authentication	Not applicable. This study did not utilize or generate any plant seed stocks or genotypes.
