# Supplementary material for: Collagen hybridizing peptide imaging and delivery of therapeutic antibody in rheumatoid arthritis models
Source: Nat Commun. 2026 Apr 20;17:5423. doi: 10.1038/s41467-026-72038-y (PMC13279783; doi:10.1038/s41467-026-72038-y)
Supplement: Supplementary file 4 — Description of Additional Supplementary Files [file 41467_2026_72038_MOESM4_ESM.pdf]

Description of Additional Supplementary Files

Supplementary Movie 1

Light-sheet fluorescence microscopy imaging showing CHP's in vivo binding to denatured collagen molecules within a cleared inflamed paw (arthritis score: 4), harvested from a CAIA mouse injected with Cy7-Ahx-K-Ahx-$(GfO)_9$ 2 h before sample collection.

Supplementary Movie 2

Light-sheet fluorescence microscopy imaging showing CHP's in vivo binding to denatured collagen molecules within a cleared non-inflamed paw (arthritis score: 0), harvested from a normal mouse injected with Cy7-Ahx-K-Ahx-$(GfO)_9$ 2 h before sample collection.
