# Supplementary material for: Collagen hybridizing peptide imaging and delivery of therapeutic antibody in rheumatoid arthritis models
Source: Nat Commun. 2026 Apr 20;17:5423. doi: 10.1038/s41467-026-72038-y (PMC13279783; doi:10.1038/s41467-026-72038-y)
Supplement: Supplementary file 2 — Transparent Peer Review file [file 41467_2026_72038_MOESM2_ESM.pdf]

nature portfolio

Peer Review File

Collagen hybridizing peptide for imaging and delivery of therapeutic antibody in rheumatoid arthritis models

Corresponding Author: Professor Yang Li

This file contains all reviewer reports in order by version, followed by all author rebuttals in order by version.

Version 0:

Reviewer comments:

Reviewer #1

(Remarks to the Author)
The authors present a valuable and extensive study supported by a large, well-curated dataset. The use of state-of-the-art imaging techniques is a major strength and enables novel insights that advance the field, while the approach of bioconjugation with mAb is innovative and carefully executed. However, several questions remain regarding the interpretation of the data and additional clarification would help strengthen the manuscript.

Major comments:
• Can the authors explain how the uptake of CHP in the pannus explained since this is targeting the degraded collagen? So how specific is the probe for depiction of degraded collagen which is not present in the pannus itself? (Fig 1)
• Do the authors have any Isotype Control for your IF staining? How do the authors set up your baseline parameters for imaging Cy3-CHP to make sure signal is not due to background signal? (Supp. Fig.6)
• The longitudinal data of the control CHP probe is not shown: how much of the signal in the joint is caused by a higher perfusion associated by increase of arthritis activity over time? Can the authors add the longitudinal data showing the increase of uptake over time and is a plateau of uptake reached? (Fig 2)
• Does the ankle thickness correlate with the fluorescence uptake too? Why did the authors choose to show right hind paws only, while Fig. 2e includes both paws? A mean or total score of both paws could also be chosen (Fig.2d-e)
• CHP binds to degraded cartilage tissue; and still they see an increase of binding as opposed to TNF binding? Could the authors explain their reasoning? (e.g. line 221/Fig3)
• A negative antibody control should be included, as the minimal binding of the TNF mAb appears unusual. The authors could check the sCHP control peptide conjugated with TNF mAb shows no or lower binding compared to the unconjugated TNF mAb. Histology in NC mice would also be a useful negative control. Finally, the phrasing around the reported affinity needs clarification — it is unclear whether the authors mean that the CHP–anti-TNF complex has higher affinity than the anti-TNF antibody alone. (Fig 3e)
• The data appear to show a similar decline between the 2 variants. Also, no clear plateau is reached, which may instead suggest slower clearance of the CHP–TNF mAb from circulation rather than enhanced or specific uptake compared to TNF mAb. (Fig. 3g, Suppl.Fig.13 b)
• Pannus in TNF mAb seems much less pronounced. Can the authors provide anatomical data (e.g. ultrasound/MRI) showing that the pannus formation was similar? (Fig.5)
• The area under the concentration-time curve for Cy7-TNFmAb-CHP was merely 28.2% that of TNFmAb within 96 h post-injection'(Line 352/353): Is this observation to be expected? The chemical structure indeed becomes larger upon conjugation of TNF mAb with CHP, which could explain the higher perfusion levels observed in the ankle. Is there PK data available for the 2 compounds in healthy mice?
• The authors should clarify the clearance route of the compounds Cy7-TNFmAb and Cy7-TNFmAb-CHP: is it primarily renal? What are the uptake differences in feces/bowels and urine? The marked difference observed in blood levels does not appear to be reflected in other organ systems; this discrepancy cannot be fully explained by the differences observed in the front paws alone. (Fig.6)

Minor comments:
• Introduction:
No comment
• Results:

Line 88: swollen swelling
Line 108: in contrast compared with (would fit better as no superlative form is used, and considering the important difference between the two collagen signals)
Line 109: “immunofluorescently” not commonly used in scientific writing, consider a more standard phrasing (by/with/using immunofluorescence)
Line 109: “upregulated usually refers to a baseline reference (upregulated compared to unstimulated, healthy control). Other phrasing such as high expression, abundance, prevalence, would be more suitable
Overall note about this part (line 106-116): co-localization can be mentioned here, any quantitative data would strengthen the interpretation and clarity of the findings (Fluorescence intensity; co-localization of signals).
Line 222: not only does not
Line 223: “confers the mAb an affinity” requires English rephrasing; Does it mean that the affinity of CHP–anti-TNF is higher than that of anti-TNF alone?
Line 237: “1.5 times greater” data not shown. AUC graph could be added to the Suppl. Fig. 13
Line 271: Why a p-value is reported in this figure but not in the others (inconsistency in reporting statistical presentation).
Line 274: The description of the histological changes could be more specific.
Line 323/324: The phrasing in this section is too vague; please provide more detailed information when comparing with the control paw.
Suppl.Fig.22: This figure would benefit from including inset coronal views, similar to those shown in Suppl.Fig.21/ Fig.5, to allow a more precise and consistent description of the results.
Line 341/342: “provided with an affinity” requires rephrasing (ref. line 223).
Line 356: The difference observed in blood in Fig.6f may be highlighted too, or alternatively, mentioned earlier alongside the plasma concentration data to fully describe the blood compartment. Additionally, only the front paws show a significant difference.
• Discussion:
Line 421/422: Examples?
Line 466: No significant differences were reported for the listed organs. Could the authors provide an explanation for the differences observed in the front paws but not in the hind paws? PK data could be more discussed here (ref. Fig.6 major comments).
• M&M:
Line 683/684: Does the 2nd Ab control contains also Cy3-CHP?

Advice:
Major revisions

Reviewer #2

(Remarks to the Author)
I co-reviewed this manuscript with one of the reviewers who provided the listed reports. This is part of the Nature Communications initiative to facilitate training in peer review and to provide appropriate recognition for Early Career Researchers who co-review manuscripts.

Reviewer #3

(Remarks to the Author)
In this manuscript, Chen et al report on the use of a CHP peptide for targeted imaging and therapy in mouse models of RA. The overall goal is to reduce off-target toxicity of immunosuppressive therapies by CHP-targeting strategy and improve imaging of collagen breakdown during disease progression and regression.

The authors validate the Cy7-labeled CHP probe in a CAIA mouse model in which they inject the and image the mice 2 hours after i.v. injection. They also use a Cy7-labeled scrambled peptide as control. The Cy7 signal is observed only in the case of CHP-tagged probe and in RA animals, conforming that the probe does not bind to healthy joints nor does the scrambled CHP bind to RA joints. They also use a Cy7-CHP that is modified with lysine in order to localize the Cy7 fluorescence by light sheet imaging. The use of this lysine-modified probe is on order to improve retention during the tissue clearing procedure.

The authors use the probe to track progression of RA and regression when RA mice are treated with DEX by both an arthritis score and the Cy7-CHP signal. The authors then show that CHP tagging enabled localization of mAB to inflamed paw. In these experiments, the authors attach a TNF-alpha mAB (TNFmAB) to CHP and Cy7 and show that this drug accumulated more in the inflamed paw compared to the TNFmAB tagged with Cy7. They further show that CHP conjugation to the mAB enhanced the efficacy of this drug based on the arthritis score, ancle thickness, and Cy7-CHP imaging. Finally, the authors also show the pharmacokinetics of the mAB-CHP conjugates.

Overall the manuscript is well constructed and well written, clear and interesting. The authors also have clear controls, and well-designed experiments. However, there remains some outstanding questions and comments which require address before publication of this manuscript:

Figure 2h: The radiant efficiency values for the normal controls is very different from that reported for normal controls in figure 1. Both distributions are very tight, so why are the values in Figure 2 almost double that in Figure 1?
Further, why is the distribution in the PBS group so varied? The values for CAIA animals injected with Cy7-CHP in figure 1 are much tighten, with standard deviation being < 1 X 108 Total radiant efficiency in hind paw in vivo. Whereas in figure 2h in PBS treated group, which presumably should not be much different than the CAIA shows a standard deviation of ~ 4 X 108 Total radiant efficiency. Explain these differences.
Figure 2H and 2J: why the differences in the statistical analysis?

Figure 3: It is not clear to me why Cy7 signal is not observed over the entire mouse body if the injection of the dye was IV. If the antibody without the CHP targeting is non-specific and has off-target effects, why is signal not observed elsewhere?

The data shows that even upon binding, the CHP derivatives washout from the target tissue within 24 hours, meaning that either binding is weak or that the bound complex is cleared from the body. Given this, the authors developed Lys-featuring CHP sequence to enhance retention, since this lysine can react with formaldehyde and neighboring protein lysines to cross-link the probe. However, this is lysine-modified derivative is a *new* and different compound. Why did the authors not use this lysine-containing probe for all studies? How does the performance of this probe compare to the non-lysine-containing probe? How does the blood half life and tissue half-life differ in these two probes? These comparisons are crucial to ensure that the introduction of this lysine does not change the pharmacokinetics and distribution of the probe.

Further, for the antibody conjugate, tissue was collected at 2 hours post injection (for data discussed staring line 308, line 312). The authors show that the antibody conjugate has slow clearance compared to CHP-Cy7, so at 2 hours post injection wouldn't there be significant non-specific binding of the probe?
The authors should show clearly the clearance half lives of both the CHP-7 and the Cy7-labeled TNFmAb-CHP from the blood of NC and both models of CAIA models, as well the normal/uninjured paw and the CAIA paws in both models. It is not clear how the timepoint for imaging and/or tissue harvest were determined.
Line 348: the authors say that the CHP-TNFmAB conjugate has a shorter blood half life than the other Cy7- TNFmAB conjugate. How can this be explained? The pharmacokinetics would be primarily governed by the large size of the antibody, and introduction of CHP would only serve to increase the size of the probe which would have either minimal effect of blood half-life or prolong blood half life. What is the blood half life of a scrambled CHP-antibody conjugate?

Why are females only used in this study?
Were the arthritis scores blinded?

Version 1:

Reviewer comments:

Reviewer #1

(Remarks to the Author)
The authors have addressed all the comments very well and have performed valuable additional experiments. The correction of the experiments in which they previously identified microaggregates, is in particular very relevant.

I have no further comments.

Reviewer #2

(Remarks to the Author)
I co-reviewed this manuscript with one of the reviewers who provided the listed reports. This is part of the Nature Communications initiative to facilitate training in peer review and to provide appropriate recognition for Early Career Researchers who co-review manuscripts.

Reviewer #3

(Remarks to the Author)
In the revision of the manuscript titled “Imaging and Therapeutic Antibody Delivery in Rheumatoid Arthritis by Collagen Hybridization” Chen et al did a admirable job addressing all the reviewer comments. Based on this and the additions to the manuscript, my recommendation is to publish the manuscript after addressing these final minor issues:
For Supplementary figure 13, please show the time points where there are significant differences between the groups

Comment 5: can you please show quantification of the DLS data? It looks to me like the CHP conjugate is much larger in size than the non-CHP conjugate, but it is unclear if this is within experimental error or not.

Does the aggregation happen with the other CHP conjugates? Have the authors verified that their other probes with CHP do not aggregate? How is this verified and is the verification done before every experiment?

Open Access This Peer Review File is licensed under a Creative Commons Attribution 4.0 International License, which permits use, sharing, adaptation, distribution and reproduction in any medium or format, as long as you give appropriate credit to the original author(s) and the source, provide a link to the Creative Commons license, and indicate if changes were made.
In cases where reviewers are anonymous, credit should be given to 'Anonymous Referee' and the source.
The images or other third party material in this Peer Review File are included in the article's Creative Commons license, unless indicated otherwise in a credit line to the material. If material is not included in the article's Creative Commons license and your intended use is not permitted by statutory regulation or exceeds the permitted use, you will need to obtain permission directly from the copyright holder.
To view a copy of this license, visit https://creativecommons.org/licenses/by/4.0/

RESPONSE TO REVIEWERS' COMMENTS

Reviewer #1 (Remarks to the Author):

The authors present a valuable and extensive study supported by a large, well-curated dataset. The use of state-of-the-art imaging techniques is a major strength and enables novel insights that advance the field, while the approach of bioconjugation with mAb is innovative and carefully executed. However, several questions remain regarding the interpretation of the data and additional clarification would help strengthen the manuscript.

Thank you for the favorable comments and constructive suggestions. Please see below our point-by-point responses to individual reviewer comments (in blue).

Major comments:

1. Can the authors explain how the uptake of CHP in the pannus explained since this is targeting the degraded collagen? So how specific is the probe for depiction of degraded collagen <u>which is not present in the pannus itself</u>? (Fig 1)

Original Fig. 1

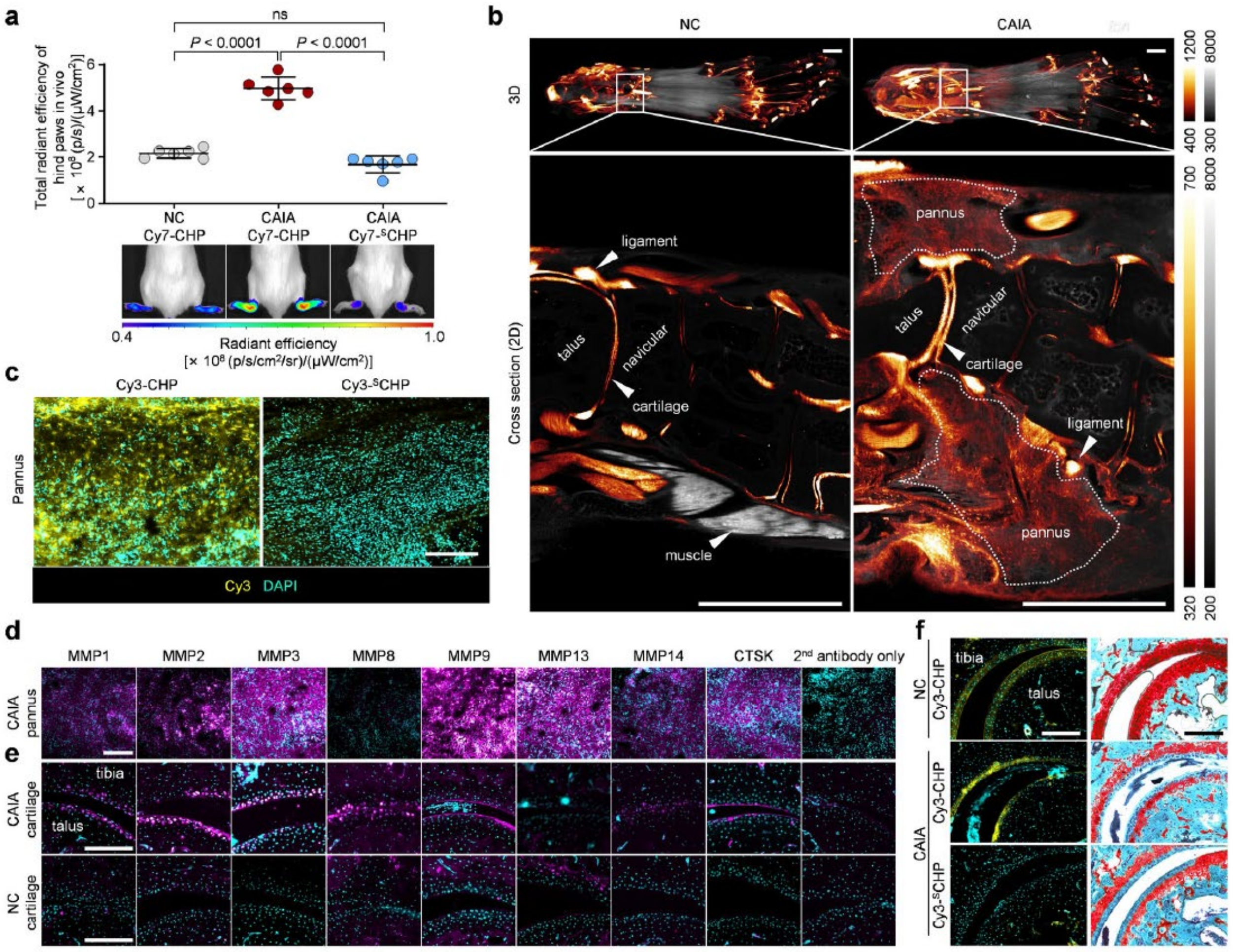

Fig. 1 | Tracing collagen breakdown in the inflamed RA joints in situ and in vivo via collagen hybridization.

Thank you for raising this important question regarding the specificity of CHP in pannus. While we respectfully disagree with the premise that degraded collagen is absent from the pannus, we agree that we need to introduce more about the composition of pannus and provide more evidence to support CHP's high specificity.

While healthy synovium (a thin delicate membrane that lines the joint capsule) is a naturally collagen-rich membrane, under inflammatory RA conditions, it transforms into a thickened and invasive pannus

characterized by both dense collagen deposition and accelerated proteolytic remodeling [ref 30: *Nat Rev Rheumatol* ***16****, 316–333 (2020). https://doi.org/10.1038/s41584-020-0413-5*]. The pannus is not merely a passive overgrowth of synovial tissue; it is characterized by high metabolic activity and the secretion of MMPs and cathepsins. These enzymes actively remodel and degrade the underlying cartilage and the internal structural collagen within the pannus itself as it expands.

To further address your concern, we have strengthened our manuscript with the following points and new data:

- *New direct evidence* (new Supplementary Fig. 9): We have included new immunofluorescence staining images of the synovium from NC mice and the pannus tissue from CAIA mice using a commercial antibody specific to degraded collagen I fragments (Adipogen Life Sciences, AG-25T-0116-C025). This antibody specifically recognizes the C-terminal end of the three-quarter fragment generated by matrix metalloproteinase cleavage of type I collagen, and it has been utilized in a dozen of published studies related to matrix remodeling. The immunofluorescence shows robust signals in the pannus of the CAIA model, whereas the fluorescence in the normal synovial tissue is almost negligible. This provides direct visual and biochemical confirmation that degraded collagen is indeed a constituent of the active pannus, serving as the fundamental premise for CHP targeting of the pannus for this study.

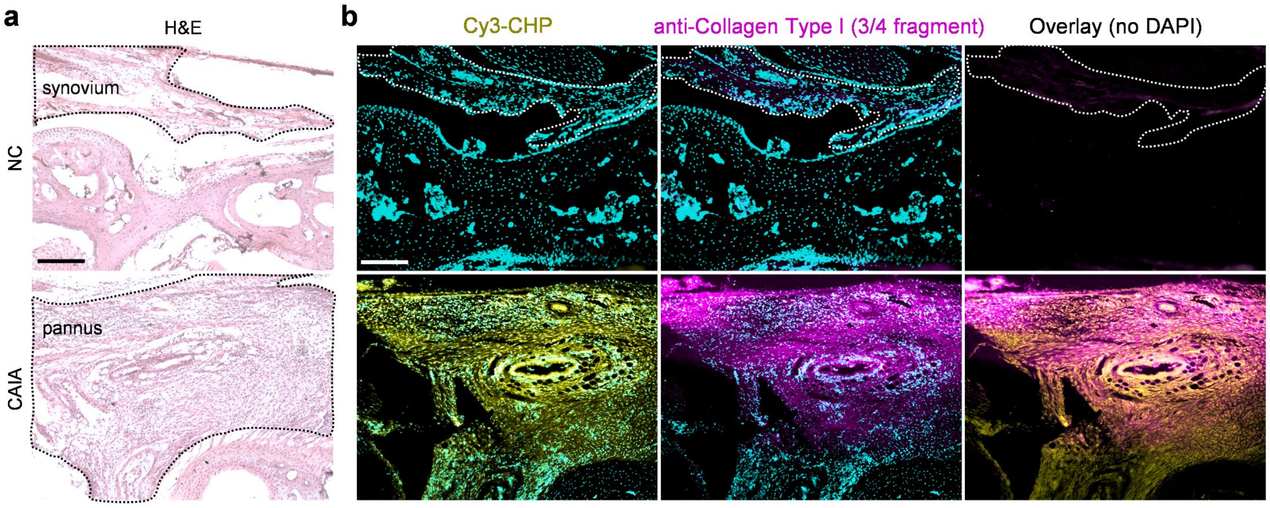

New **Supplementary Fig. 9. a** Representative H&E staining of the cryosections of the normal and CAIA mice's ankle tissue. **b** Representative fluorescence micrographs of the cryosections of the normal and CAIA mice's ankle tissue that were co-stained with Cy3-CHP and an antibody against degraded collagen I fragment, which was detected with an Alexa Fluor 647 labeled secondary antibody (goat anti-rabbit IgG H&L). Cyan: DAPI. Scale bars: 250 μm (**a**, **b**).

Also, as demonstrated in the images above, the minimal Cy3-CHP fluorescence observed in healthy synovial tissue stands in stark contrast to the robust and extensive Cy3-CHP signals localized within the RA pannus. This difference highlights the probe's selectivity for the degraded and denatured collagen generated during the pathological remodeling of the RA synovium.

- *Indirect evidence reiteration:* As noted in our original submission, we have provided evidence for the pannus' rich collagen content (original Supplementary Fig. 5) and the extensive MMP expression in the pannus of the CAIA mice (original Fig. 1e):

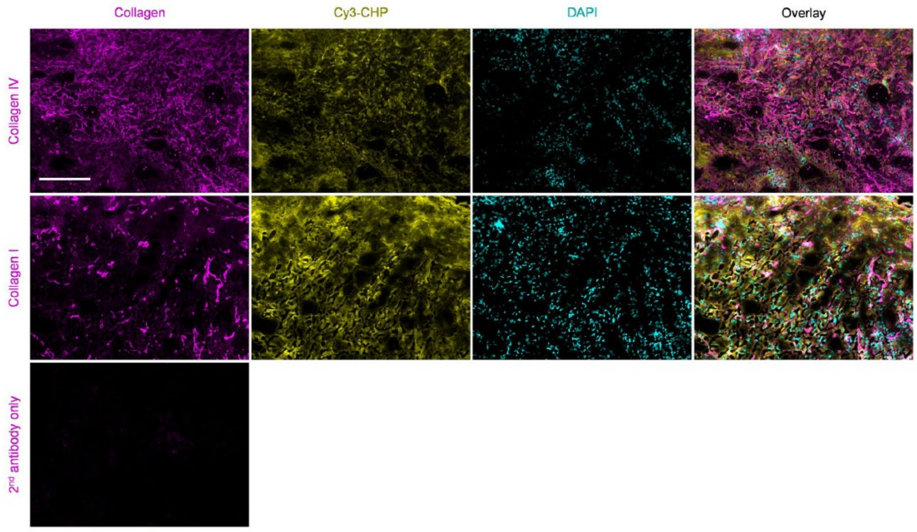

Original **Supplementary Fig. 5.** Representative fluorescence micrographs of the cryosections of the ankle synovial pannus from the CAIA mice. Each section was co-stained with DAPI, Cy3-CHP, and an antibody against collagen I or IV. All primary antibodies were detected with an Alexa Fluor 647 labeled secondary antibody. The sections in the 2nd-antibody-only group were stained without primary antibodies. Scale bar: 150 μm.

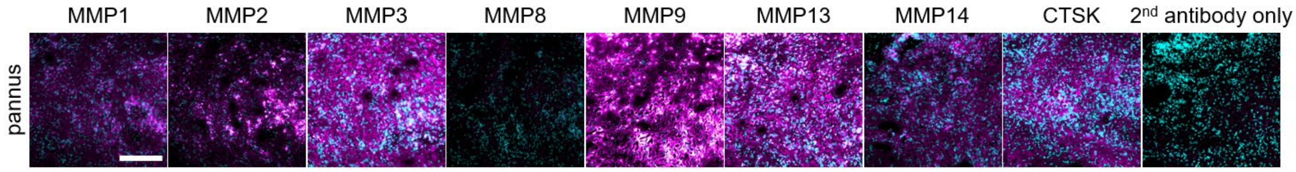

Fig. 1e. Representative fluorescence micrographs of the cryosections of the CAIA mice's ankle pannus tissue immunostained against various MMPs and cathepsin K (CTSK). The sections in the 2nd-antibody-only group were stained without any primary antibody. Scale bar: 150 μm.

We believe that the above-mentioned points and data are sufficient to explain the uptake of CHP in the pannus. Yet, we have collected one final set of new data from a blocking experiment to fully demonstrate that the CHP's binding in the pannus is specific to degraded collagen. As shown in New Supplementary Fig 7, pre-treating the pannus cryosection with a 20-fold molar excess of Cy5-CHP abolished the Cy3-CHP signal, whereas pre-incubation with an equivalent amount of the sequence scrambled control peptide Cy5-^{S}CHP showed a negligible effect on Cy3-CHP binding. These results confirm that the probe's localization is driven by sequence-specific hybridization to denatured collagen strands rather than non-specific electrostatic or hydrophobic interactions within the hyperplastic tissue.

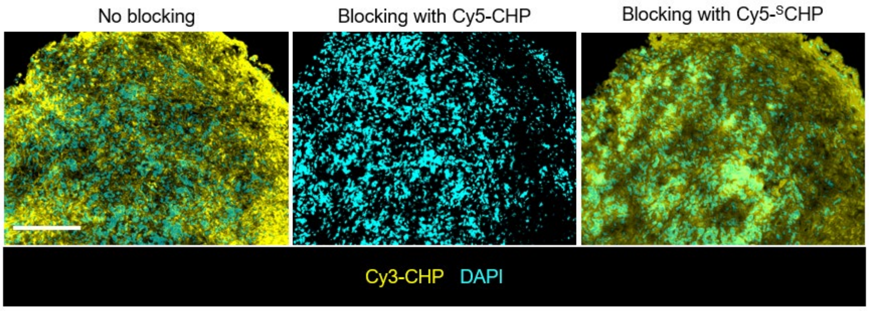

New **Supplementary Fig. 7**. Fluorescence micrographs of neighboring cryosections from the ankle synovial pannus of CAIA mice, all stained with Cy3-CHP (10 μM) for 6 h at 4 °C after the section was pre-treated with PBS (no blocking), excessive (200 μM) Cy5-CHP or the sequence-scrambled control peptide Cy5-^{S}CHP. Scale bars: 150 μm.

In addition to the new experimental data and figures, we have updated the corresponding sections in the Results and Methods to integrate these findings and clarify the relationship between pannus hyperplasia and collagen turnover.

Results:

Histological analysis of inflamed ankle cryosections revealed robust Cy3-CHP fluorescence extensively distributed throughout the pannus, a hyperplastic and collagen-rich synovial tissue (Fig. 1c)[30]. CHP fluorescence signals preferentially colocalized with collagen IV immunostaining rather than collagen I (Supplementary Fig. 6). The absence of binding for the sequence-scrambled control (Cy3-^{S}CHP) suggests that triple-helical hybridization is required for target engagement (Fig. 1c), a finding further supported by the competitive blocking experiments (Supplementary Fig. 7). Moreover, immunofluorescence analysis revealed prevalence of MMP1, 2, 3, 9, 13, 14 (esp. MMP1, 3, 9, and 13) and CTSK in the pannus cryosections (Fig. 1d) surrounded by the Cy3-CHP-marked denatured collagen (Supplementary Fig. 8), suggesting extensive matrix degradation. Strikingly, Cy3-CHP co-localized with the immunofluorescence from a commercial antibody targeting degraded collagen specifically in the pannus, but not the normal synovium (Supplementary Fig. 9). This finding directly supports the peptide's specific binding to degraded collagen fragments in the pannus.

Updates in Methods:

antifade mounting medium. Cryosections of the hind paws pre-stained with Cy3-CHP before sectioning were also used and probed with primary antibodies targeting MMPs, CTSK, and Col I 3/4 fragments (Adipogen Life Sciences, AG-25T-0116-C025, 1:200) overnight at 4 °C (Fig. 1e, Supplementary Figs. 8–10) as described above. Stained slides were imaged using the EVOS M7000 imaging system (light cubes: RFP, Cy5, and DAPI) with the following parameters. Pseudo colors were assigned to the images using the LUT color scheme available in the ImageJ software.

For Supplementary Fig. 7, pannus cryosections from a CAIA model mouse were washed with PBS to remove OCT. For competitive blocking, Cy5-CHP or Cy5-^{S}CHP (200 μM in 100 μL of PBS) were pre-heated at 80 °C for 10 min, immediately cooled down, and applied to the sections for overnight incubation at 4 °C [Cy5-CHP: Cy5-Ahx-(GPO)$_9$, m/z calculated: 3181.4 [M+Na]$^+$, m/z found: 3182.1 [M+Na]$^+$; Cy5-^{S}CHP: Cy5-Ahx-PGOGPGPOPOGOGOPPGOOPGGOOPPG, m/z calculated: 3181.4 [M+Na]$^+$, m/z found: 3182.1 [M+Na]$^+$). After removing the Cy5-peptide solutions, the sections were incubated with Cy3-CHP (10 μM in 100 μL PBS) at 4 °C for 6 h, followed by DAPI staining and PBS washes. Imaging was performed on an EVOS M7000 system (20× objective, Cy3: RFP cube, Light: 0.5, Exposure: 0.25, Gain: 10).

2. Do the authors have any Isotype Control for your IF staining? How do the authors set up your baseline parameters for imaging Cy3-CHP to make sure signal is not due to background signal? (Supp. Fig.6)

Original Supplementary Fig. 6

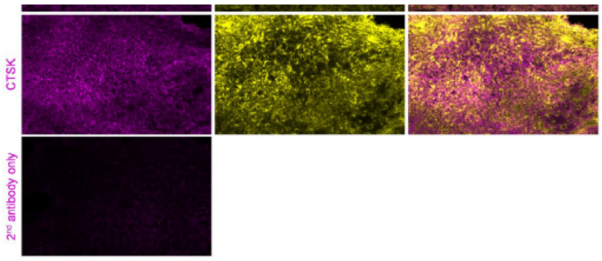

Supplementary Fig. 6. Representative fluorescence micrographs of the cryosections of the ankle synovial pannus from the CAIA mice. Each section was co-stained with Cy3-CHP and an antibody against an MMP subtype or cathepsin K (CTSK). All primary antibodies were detected with an Alexa Fluor 647-labeled secondary antibody. The sections in the 2nd-antibody-only group were stained without primary antibodies. Scale bar: 150 μm.

Thank you for these technical inquiries.

(1) Regarding your inquiry about isotype controls for the immunofluorescence staining, we have addressed this by utilizing several layers of internal and experimental validation.

First, the primary antibodies used for labeling the MMPs and Cathepsin K demonstrated negligible staining in healthy cartilage tissues (Fig. 1d). This lack of background in healthy tissue serves as a robust internal negative control, confirming that these commercial antibodies do not bind non-specifically to intact extracellular matrix components.

Second, we emphasize that the high expression of proteases like MMPs and Cathepsin K in the RA pannus and at the cartilage-pannus junction is a well-established pathological hallmark in the literature; our immunofluorescence data is intended as supporting evidence to contextualize the local collagen degradation detected by our CHP probe.

Finally, we performed a secondary-antibody-only control (where the primary antibody was replaced with buffer), which yielded no signal (under the same imaging parameters), further ruling out non-specific binding of the secondary antibody or tissue autofluorescence.

Collectively, we think the high biological consistency of our results, where protease expression spatially aligns in general with CHP-detected collagen damage, supports the specificity of our findings without the need for additional isotype controls.

(2) To ensure the Cy3-CHP signals are specific and not a result of tissue autofluorescence or non-specific probe retention, we standardized our imaging protocol using a sequence-scrambled control peptide (Cy3-^{S}CHP). Baseline acquisition parameters, including excitation light intensity, exposure, and most importantly, gain, were first calibrated on the ^{S}CHP-stained pannus sections to a level where no background was detectable. These identical parameters were then strictly maintained for all subsequent Cy3-CHP imaging to ensure an objective comparison of fluorescence intensity.

While this control data was originally omitted for conciseness, we have now included it in the updated Supplementary Fig. 6 (now as **Supplementary Fig. 8**) as well as Supplementary Fig. 7 (now as **Supplementary Fig. 10**). As demonstrated below, the Cy3-^{S}CHP exhibits virtually no background signal in the pannus and cartilage, standing in stark contrast to the robust and localized Cy3-CHP signal. This direct comparison confirms that our imaging parameters effectively eliminate background interference and that the observed fluorescence is a result of sequence-specific hybridization to denatured collagen.

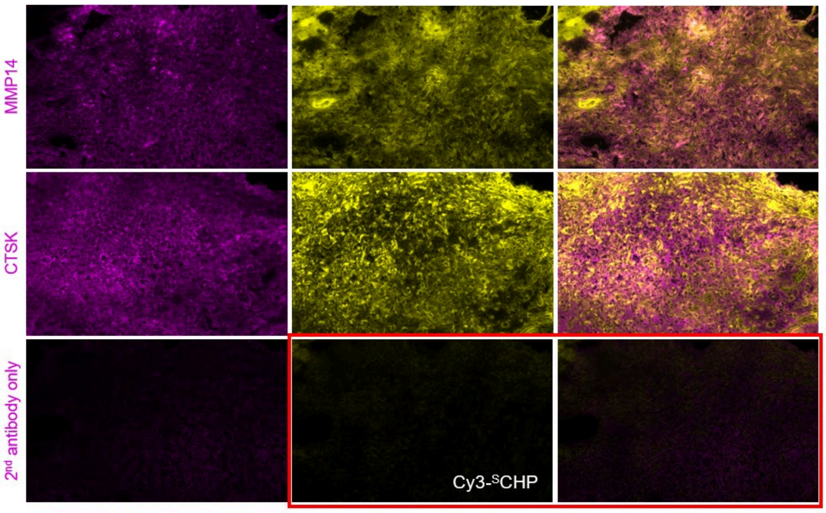

Supplementary Fig. 8 (originally Supplementary Fig. 6). Representative fluorescence micrographs of the cryosections of the ankle synovial pannus from the CAIA mice. Each section was co-stained with Cy3-CHP (or Cy3-^{S}CHP for one set of sections as a negative control, bottom panels) and an antibody against an MMP subtype or cathepsin K (CTSK). All primary antibodies were detected with an Alexa Fluor 647-labeled secondary antibody. The sections in the 2nd-antibody-only group were stained without primary antibodies. Scale bar: 150 µm.

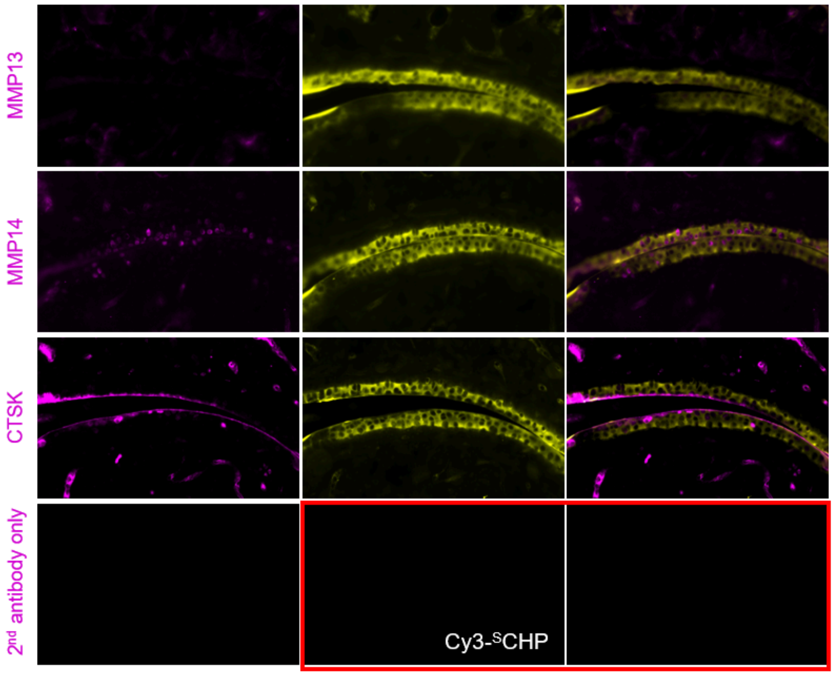

Supplementary Fig. 10 (originally Supplementary Fig. 7). Representative fluorescence micrographs of the cryosections of the cartilage between the tibia and talus from the inflamed ankles of the CAIA mice. The paws were stained with Cy3-CHP (presumably capable of binding to denatured collagen II in the cartilage) (or Cy3-^{S}CHP for one paw as a negative control, bottom panels) before decalcification and stained with an antibody against an MMP subtype or CTSK on the cryosections. All primary antibodies were detected with an Alexa Fluor 647 labeled secondary antibody. The sections in the 2nd-antibody-only group were stained without primary antibodies. Scale bar: 150 µm.

We have updated the Methods section to explicitly describe this normalization procedure.

sections. For fluorescence imaging of bound Cy3-CHP (Fig. 1c, 1f, and Supplementary Fig. 10), cryosectioned slides were rinsed with PBS for 5 min to remove OCT, stained with DAPI (Beyotime C1002, 1:1000 dilution in ddH_2O) for 15 min, sealed in an antifade mounting medium (Vector, H-1000) and imaged with an EVOS M7000 imaging system (Thermo Fisher) (Light cubes: RFP and DAPI, objective: 20×). To ensure signal specificity and eliminate background interference, imaging parameters, including excitation intensity, exposure time, and most importantly, detector gain, were first calibrated using pannus sections stained with the sequence-scrambled control Cy3-^{S}CHP to establish a baseline of negligible fluorescence, with these identical settings strictly maintained for all Cy3-CHP acquisitions. Pseudocolors were assigned to the images using the LUT color scheme available in the ImageJ software (Fiji 2.14.0). Adjacent sections were then stained with Safranin-O/fast green staining kit (Solarbio, G1371).

3. The longitudinal data of the control CHP probe is not shown: how much of the signal in the joint is caused by a higher perfusion associated by increase of arthritis activity over time? Can the authors add the longitudinal data showing the increase of uptake over time and is a plateau of uptake reached? (Fig 2)

Original Fig. 2

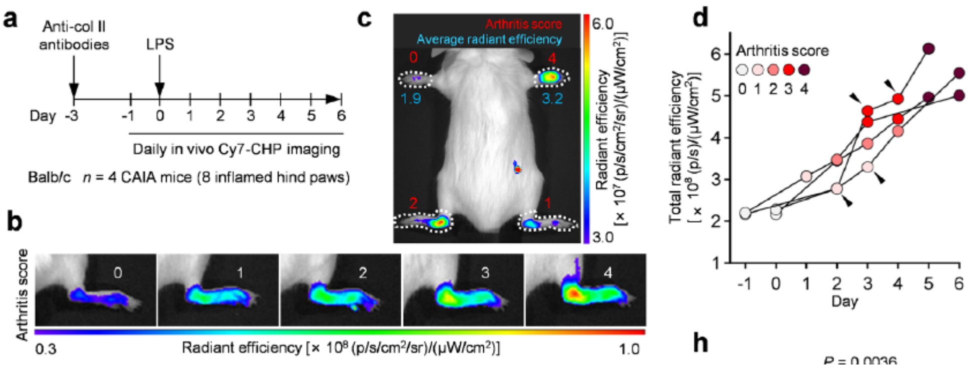

Fig. 2 | Measuring and live monitoring changes in collagen destruction in the inflamed ankles.

We sincerely thank you for raising this critical point regarding the potential for non-specific probe accumulation driven by the increased perfusion and vascular permeability characteristic of inflamed joints.

Accordingly, we have performed an additional longitudinal study using the sequence-scrambled control probe (Cy3-^{S}CHP) across the same disease progression timeline as our primary data to rigorously distinguish between specific collagen binding and non-specific retention. As now presented in **Supplementary Fig 11b**, our results demonstrate that while the arthritis scores and associated inflammation increase over time, the signal from the scrambled control probe remains consistently at baseline levels.

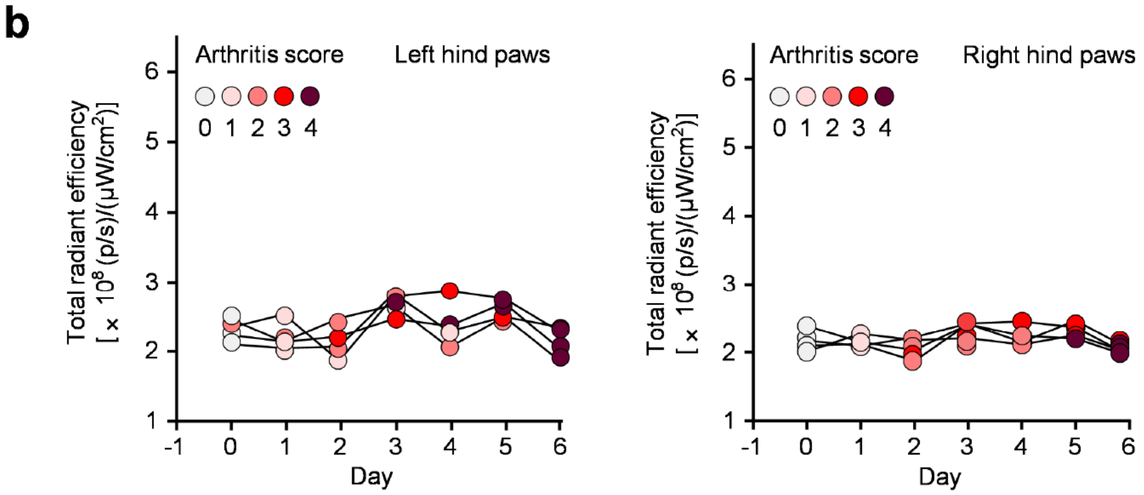

Supplementary Fig. 11 b Quantified in vivo Cy7-^{S}CHP fluorescence signals in the left and right hind paws of each CAIA mouse from the day before to 6 days after the LPS injection. The left and right paws were shown in separate figures to avoid over-crowding and to allow tracking of the changes of induvial paws.

This divergence, where the Cy3-CHP signal increases in correlation with disease severity while the ^{S}CHP signal does not, directly confirms that the joint enrichment of our probe is a function of specific molecular hybridization to denatured collagen rather than a passive result of hyperperfusion or inflammatory swelling.

We have updated the **Results section** to include these vital control data.

Dynamic monitoring of RA progression and regression

Upon confirming that the 1 nmole i.v. dose of Cy7-CHP can be entirely cleared from both inflamed and normal ankle joints within 24 h (Supplementary Fig. 2), we proceeded to monitor collagen degradation in the hind paws of four CAIA mice throughout the disease onset and progression via daily Cy7-CHP injection and live imaging (Fig. 2a). Quantification of in vivo Cy7-CHP fluorescence in individual hind paws revealed a progressive increase in signal intensity over the six days following LPS injection, closely correlating with the rising arthritis scores (Fig. 2b–d and Supplementary Fig. 11a). In contrast, the signal from the scrambled control probe ^{S}CHP remained consistently at baseline levels while the arthritis scores and associated inflammation increase over time (Supplementary Fig.11b). This divergence demonstrates that the observed joint enrichment is a result of specific CHP hybridization to the progressive accumulation of denatured collagen, rather than a passive byproduct of hyperperfusion or vascular permeability. Moreover, slight increase of the Cy7-CHP signal was

Updated description of Fig. 2d

each paw's fluorescence uptake quantification that corresponds to its arthritis score. **d** Quantified in vivo Cy7-CHP fluorescence signals in the right hind paw of each CAIA mouse (left hind paws: Supplementary Fig. 11a; Cy7-^{S}CHP: Supplementary Fig. 11b) from the day before to 6 days after the LPS injection. Arrows: moderately increasing Cy7-CHP uptake in the same paws over 24 h without change in the arthritis score. **e** Correlation analysis between the Cy7-CHP fluorescence uptake and the

4. Does the ankle thickness correlate with the fluorescence uptake too? Why did the authors choose to show right hind paws only, while Fig. 2e includes both paws? A mean or total score of both paws could also be chosen (Fig.2d-e)

Original Figs. 2d,e

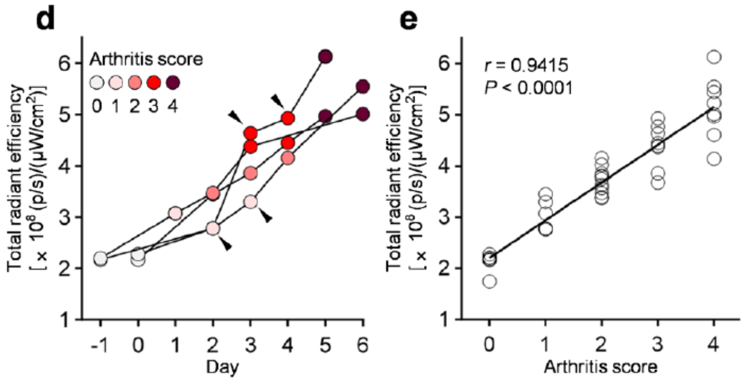

Fig. 2 | Measuring and live monitoring changes in collagen destruction in the inflamed ankles.

Thank you for your inquiry into the correlation between ankle thickness and fluorescence uptake, as well as our rationale for the presentation of paw-specific data.

1) Does the ankle thickness correlate with the fluorescence uptake too?

Yes. During the CAIA study, we systematically recorded ankle thickness, arthritis scores, and fluorescence uptake daily for all mice. In the original submission, we focused on the correlation between Cy7-CHP signal and the arthritis scores, as it is a comprehensive metric that inherently incorporates joint swelling. Per your suggestion, we have now performed a direct correlation analysis between ankle thickness and fluorescence intensity. The results reveal a robust and significant positive correlation (Pearson $r = 0.9263$, $P < 0.001$), which has been added to the revised manuscript as **Supplementary Fig. 12b**. This further validates that Cy7-CHP uptake accurately reflects the physical progression of joint inflammation and remodeling.

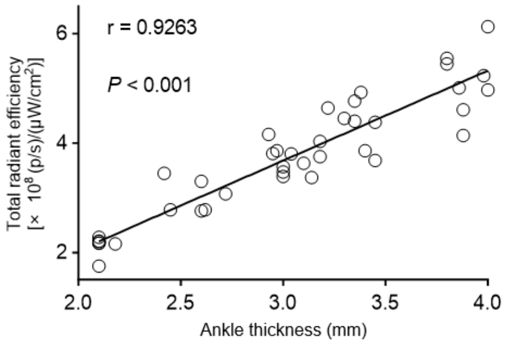

Supplementary Fig. 12 b Correlation analysis between the Cy7-CHP fluorescence uptake and the corresponding ankle thickness of all 8 hind paws of the 4 mice studied in Fig. 2a. Statistical analysis: Pearson correlation coefficient.

2) Why did the authors choose to show right hind paws only, while Fig. 2e includes both paws? A mean or total score of both paws could also be chosen.

Regarding the choice to display only the right hind paws in Fig. 2d: our primary objective in these panels was to demonstrate the longitudinal evolution profile of the signal within individual paws using a line chart. Including all eight hind paws from 4 mice in a single plot creates significant element overlapping, making it difficult to track the trajectories of individual paws. So initially, we chose to only show the right hind paws in Fig. 2d.

To maintain visual clarity while ensuring full data transparency, we have now provided the corresponding longitudinal data for the left hind paws separately in **Supplementary Fig 11a**.

We think that it is more helpful to present individual paw data rather than a mean or total score of both paws, because individual data points provide a more granular and precise assessment of the probe's performance, demonstrating its sensitivity to varying degrees of localized inflammation that may otherwise be masked by averaging.

a

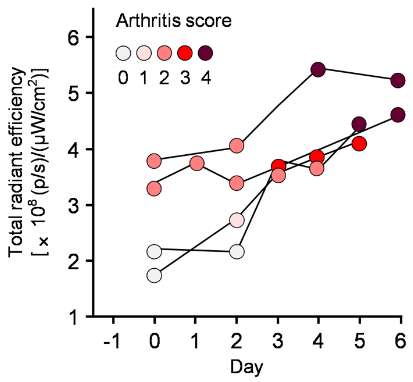

Supplementary Fig. 11a Quantified in vivo Cy7-CHP fluorescence signals in the left hind paw of each CAIA mouse in Fig. 2d from the day before to 6 days after the LPS injection. The left and right paws were shown in separate figures to avoid over-crowding and to ensure tracking of the changes of induvial paws.

We have also updated the corresponding section in Results to incorporate these new data sets.

Dynamic monitoring of RA progression and regression

Upon confirming that the 1 nmole i.v. dose of Cy7-CHP can be entirely cleared from both inflamed and normal ankle joints within 24 h (Supplementary Fig. 2), we proceeded to monitor collagen degradation in the hind paws of four CAIA mice throughout the disease onset and progression via daily Cy7-CHP injection and live imaging (Fig. 2a). Quantification of in vivo Cy7-CHP fluorescence in individual hind paws revealed a progressive increase in signal intensity over the six days following LPS injection, closely correlating with the rising arthritis scores (Fig. 2b–d and Supplementary Fig. 11a). In contrast, the signal from the scrambled control probe ^{S}CHP remained consistently at baseline levels while the arthritis scores and associated inflammation increase over time (Supplementary Fig.11b). This divergence demonstrates that the observed joint enrichment is a result of specific CHP hybridization to the progressive accumulation of denatured collagen, rather than a passive byproduct of hyperperfusion or vascular permeability. Moreover, slight increase of the Cy7-CHP signal was sensitively detected on sequential days even for paws with no change in the arthritis score (arrowheads, Fig. 2d). The varying CHP fluorescence levels in the four paws of a single mouse typically corresponded with each paw's arthritis score (Fig. 2c). Statistically, the CHP fluorescence uptake in the hind paws measured from all images over the experimental duration was differentiable according to the arthritis score (Supplementary Fig. 12a); furthermore, strong positive correlations were observed between in vivo CHP fluorescence uptake and arthritis score (Spearman r = 0.9415, Fig. 2e) as well as between in vivo CHP uptake and ankle thickness (Pearson r = 0.9263, Supplementary Fig. 12b).

5. CHP binds to degraded cartilage tissue; and still they see an increase of binding as opposed to TNF binding? Could the authors explain their reasoning? (e.g. line 221/Fig3)

6. A negative antibody control should be included, as the minimal binding of the TNFmAb appears unusual. The authors could check the ^{S}CHP control peptide conjugated with TNFmAb shows no or lower binding compared to the unconjugated TNFmAb. Histology in NC mice would also be a useful negative control. Finally, the phrasing around the reported affinity needs clarification — it is unclear whether the authors mean that the CHP–anti-TNF complex has higher affinity than the anti-TNF antibody alone. (Fig 3e)

Original line 221:

antibody (Supplementary Fig. 12b). Importantly, the Cy7-TNFmAb-CHP's binding to the cryosections of the synovial pannus of the CAIA mice was strikingly greater than that of Cy7-TNFmAb (Fig. 3e).

Original Fig. 3d-e:

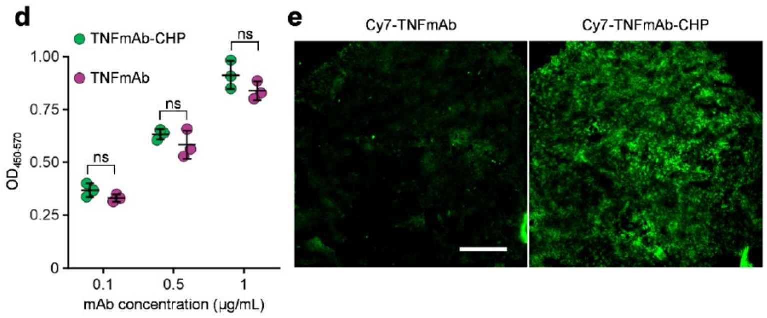

Fig. 3d ELISA assays comparing the reactivity of TNFmAb-CHP and TNFmAb against the recombinant mouse TNF-α protein (*n* = 3 independent experiments)

Fig. 3e Representative fluorescence micrographs of cryosections of the CAIA mice's synovial pannus stained with Cy7-labeled TNFmAb-CHP or TNFmAb.

We fully agree with your assessment. The CHP peptide is a collagen-targeting peptide and does not possess the biochemical capacity to enhance the intrinsic affinity of an mAb for TNF-α. This is consistent with our data in **Fig. 3d**, which shows no significant difference in the binding affinity to TNF-α between TNFmAb and the TNFmAb-CHP conjugate.

Our findings instead describe a dual-targeting mechanism: the CHP moiety confers the *conjugate* with the ability to hybridize specifically with degraded and denatured collagen chains. This allows a higher density of TNFmAb-CHP to be retained within the pannus tissue of RA joints compared to TNFmAb alone at the same concentration. While a portion of the conjugate binds to soluble or membrane-bound TNF-α, a substantial fraction is anchored via the CHP-collagen interaction within the inflamed and damaged extracellular matrix. **Fig. 3e** was intended to demonstrate this differential tissue accumulation.

To address your specific concerns regarding the controls and phrasing, we have performed additional experiments and revised the manuscript as follows:

(i) Inclusion of a negative antibody control: Following your suggestion to include a negative antibody control, we have updated **Fig. 3e**. We utilized a Cy7-labeled secondary antibody as a negative control. Under higher-sensitivity imaging parameters, the secondary antibody showed minimal non-specific background, whereas Cy7-TNFmAb exhibited a distinct signal, confirming the antibody's specific binding to endogenous TNF-α in the pannus tissue. Notably, under the same imaging parameters, the Cy7-TNFmAb-CHP group showed overexposed fluorescence. This confirms that the CHP-mediated hybridization with unfolded collagen significantly increases the total amount of the conjugate anchored within the inflamed pannus matrix.

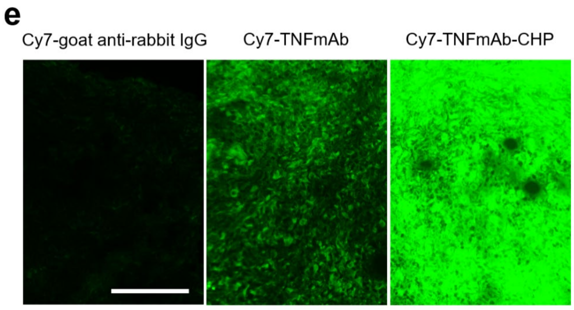

Updated Fig. 3e Representative fluorescence micrographs of cryosections of the CAIA mice's synovial pannus stained with Cy7-TNFmAb, Cy7-TNFmAb-CHP, or a Cy7-labeled goat-anti-rabbit IgG (2nd antibody).

Furthermore, we added a competitive blocking experiment to confirm that such increase in binding of the Cy7-TNFmAb-CHP conjugate to the pannus tissue was predominantly mediated by the CHP-collagen hybridization.

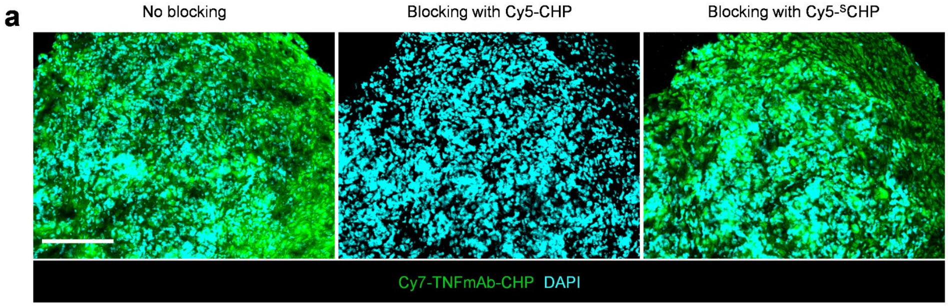

Supplementary Fig. 17. a Representative fluorescence micrographs of the cryosections from the ankle synovial pannus of CAIA mice, stained with Cy7-TNFmAb-CHP (200 µg/mL) overnight at 4 °C. Staining was performed with or without pre-blocking with either a high dose of Cy5-CHP or the sequence-scrambled control peptide Cy5-^{S}CHP (200 µM) overnight.

(ii) Histology in Normal Control mice: In line with your suggestion, we have added histological analysis of synovial tissue from NC mice. Our results show that Cy7-TNFmAb exhibits negligible signal in normal synovial tissue but displays extensive binding in the RA pannus tissue. This result clearly validates the specificity of the antibody.

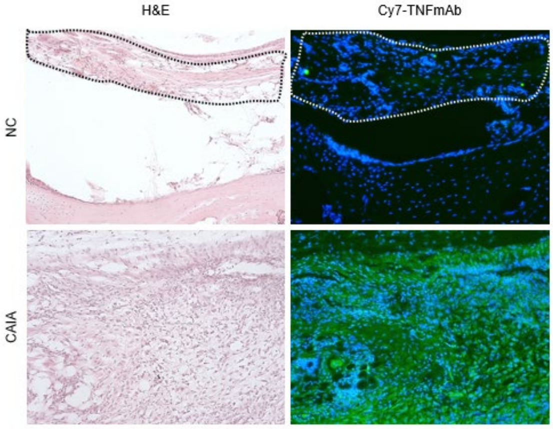

Representative fluorescence micrographs of cryosections of the NC mice's healthy synovial tissue (dotted circle) and CAIA mice's pannus stained with Cy7-labeled TNFmAb (green) and DAPI (blue).

(iii) Clarification of phrasing: We have carefully revised the manuscript to eliminate any ambiguity regarding the antibody "affinity". We now explicitly state that the TNFmAb-CHP conjugate gains an *additional binding capacity* toward the damaged collagen matrix compared to TNFmAb itself, rather than an *increased* affinity for the TNF-α cytokine itself.

Examples of these revisions include:

antibody (Supplementary Fig. 16b). In cryosections, although Cy7-TNFmAb exhibited preferential binding to the inflamed pannus of the CAIA mice; however, conjugation with CHP enhanced tissue accumulation multi-fold (Fig. 3e). Blocking experiments confirmed that this increased binding to the tissue is primarily driven by CHP-mediated hybridization with degraded collagen (Supplementary Fig. 17a). Furthermore, the Cy7-TNFmAb-CHP conjugate showed minimal binding to normal synovial tissues compared to the RA pannus (Supplementary Fig. 17b). Collectively, these in vitro and histological results demonstrate that the CHP conjugation preserves the intrinsic antigen-recognition capability of TNFmAb while endowing the conjugate a second affinity for the denatured collagen prevalent in the RA joint matrix.

Furthermore, we demonstrated that the CHP peptide selectively binds to the inflamed synovial pannus of a collagen-induced arthritis (CIA) mouse model, as well as to the arthritic shoulder joint cartilage from an RA patient (Supplementary Figs. 29 & 30). Crucially, this collagen hybridization-mediated binding also enabled the TNFmAb-CHP conjugate to adhere to these pathological tissues (Supplementary Figs. 29 & 30). These data implied that our targeting strategy would be effective across different RA models and potentially in human RA.

We also updated the related parts in Methods:

Section staining with Cy7-labeled TNFmAbs

Pannus tissue was carefully dissected from the inflamed paw of a CAIA mouse and cryosectioned into 4 µm sections. After the OCT was removed with PBS, sections were incubated in 0.3% H_2O_2 for 20 min, blocked with 10% goat serum for 1 h, incubated with Cy7-goat anti-rabbit IgG (Cohesion Bioscience, CSA4611) , Cy7-TNFmAb, or Cy7-TNFmAb-CHP (200 µg/mL in PBS) overnight at 4 °C. Slides were mounted with antifade mounting medium the next day, followed by microscopic imaging using the EVOS M7000 imaging system (Light cube: Cy7, objective: 20×).

For the competitive blocking assay described in Supplementary Fig. 17a, pannus cryosections were first treated at 4 °C overnight with 200 µM Cy5-CHP (sequence: Cy5-Ahx-$(GfO)_9$; m/z calculated: 3343.3 $[M+Na]^+$, m/z found: 3343.8 $[M+Na]^+$) and Cy5-^{S}CHP (sequence: Cy5-Ahx-OfGGOfGfGfOfOGOfGOOfGGOOffG; m/z calculated: 3343.3 $[M+Na]^+$, found: 3343.8 $[M+Na]^+$), which were pre-heated at 45 °C for 5 min. The sections were then probed with Cy7-TNFmAb-CHP (200 µg/mL in 100 µL PBS) for 6 h at 4 °C, stained with DAPI, washed, and imaged using EVOS M7000 system (20× objective, Cy7: Cy7 cube, Light: 0.5, Exposure: 0.25, Gain: 20).

7. Pannus in TNF mAb seems much less pronounced. Can the authors provide anatomical data (e.g. ultrasound/MRI) showing that the pannus formation was similar? (Fig.5)

Originally Fig. 5c

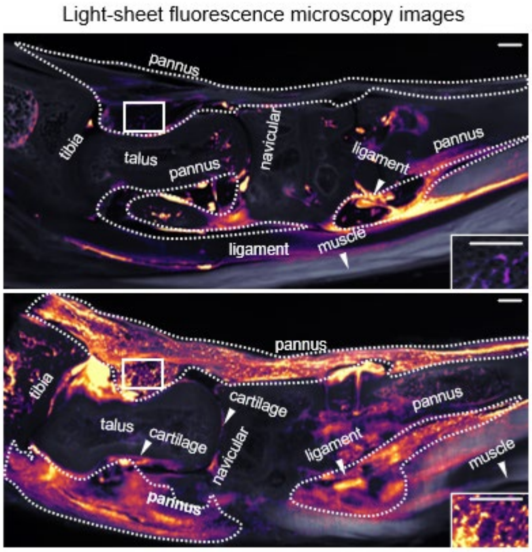

Representative light-sheet fluorescence microscopy images showing the distinct anatomical locations of Cy7-TNFmAb and Cy7-TNFmAb-CHP distribution within the arthritic hind paws (arthritis score: 2) in 2D sagittal view (Bottom right thumbnails: zoomed-in images for the boxed areas). Scale bars: 250 μm.

We thank you for raising this important point. We fully understand the concern that potential variations in pannus formation between individual animals could confound the assessment of targeting efficiency. To ensure experimental rigor and comparability between groups, we implemented the following measures and performed additional validation.

In our experimental design, all mice used for light-sheet fluorescence microscopy in Fig. 5 were carefully selected based on synchronized disease progression. Specifically, we used right hind paw samples from CAIA mice that shared an identical clinical arthritis score (a score of 2) at the time of imaging.

Anatomical locating CHP-delivered TNFmAb in the arthritic paws

We traced the distribution of the CHP-conjugated TNFmAb-CHP within the anatomical locations of the inflamed hind paws of the CAIA mice using light-sheet microscopy. On day 2 post-LPS-induction of arthritis in the right hind footpad (Fig. 5a, arthritis score: 2), both mice were intravenously injected with an equal dose of Cy7-labeled TNFmAb-CHP or TNFmAb. Two hours post-injection, their hind

The pronounced difference in fluorescence distribution and intensity between the Cy7-TNFmAb and Cy7-TNFmAb-CHP groups is a direct result of their differing targeting capacity, rather than a discrepancy in the severity of the pannus. In the original images in **Fig. 5c**, the Cy7-TNFmAb signal appears faint and fails to outline the pannus. This is not due to an absence of pannus tissue in that animal, but rather the limited targeting and retention capacity of the unconjugated antibody within the synovial matrix. In contrast, the Cy7-TNFmAb-CHP conjugate specifically hybridizes with the degraded collagen matrix within the inflamed tissue, allowing the signal to strongly and specifically delineate the pathological profile of the pannus.

To provide definitive evidence that the disease severity was equivalent between the two groups, we performed a post-hoc histological analysis—the gold standard for evaluating pannus severity. Following lightsheet imaging, the exact hind paw samples from **Fig. 5** were rehydrated and processed for paraffin sectioning and H&E staining.

As shown in the newly added **Supplementary Fig. 26**, the histopathological evaluation clearly demonstrates that the extent and severity of pannus formation were comparable between the Cy7-TNFmAb and Cy7-TNFmAb-CHP treatment groups.

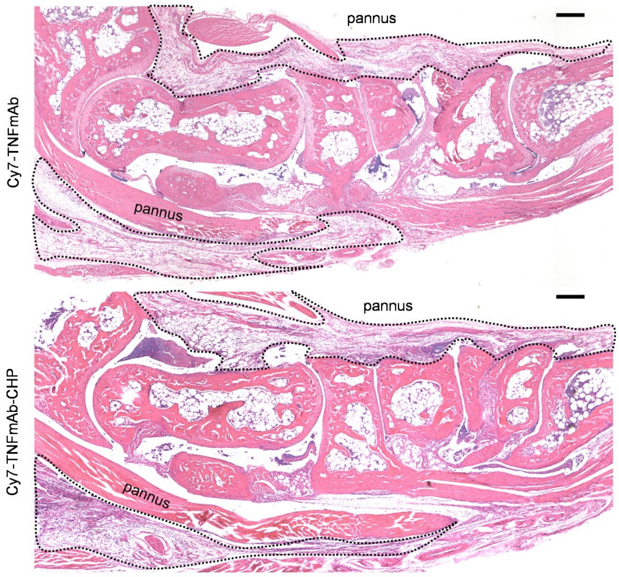

Supplementary Fig. 26 Representative H&E staining of paraffin-embedded sections of the ankle joints prepared from Fig. 5. Following light-sheet fluorescence imaging, the cleared tissues were rehydrated, processed for paraffin sectioning and stained with H&E. Scale bars: 100 μm.

We have also updated the corresponding sections in the Results.

CHP, but not Cy7-TNFmAb (Fig. 5c-e). The only fluorescence that could be seen in the magnified fields of view of the pannus was from the Cy7-TNFmAb-CHP bound to the tissue (granular signals, Fig. 5c inset). Critically, this difference in signal intensity was not attributable to variations in disease severity. Histopathological analysis of the same paw specimens post light-sheet microscopy confirmed equivalent levels of RA-associated pathology across both groups (Supplementary Fig. 26). Furthermore, in stark contrast to Cy7-TNFmAb, Cy7-TNFmAb-CHP demonstrated substantial uptake within the inflamed interphalangeal joints (Fig. 5f and b, arrowheads). Meanwhile, we noted that the

8. The area under the concentration-time curve for Cy7-TNFmAb-CHP was merely 28.2% that of TNFmAb within 96 h post-injection' (Line 352/353): Is this observation to be expected? The chemical structure indeed becomes larger upon conjugation of TNF mAb with CHP, which could explain the higher perfusion levels observed in the ankle. Is there PK data available for the 2 compounds in healthy mice?

Original lines 350-352:

was notably higher from 2 h (Fig. 6b, c). Indeed, with the identical dosage, the area under the concentration-time curve for Cy7-TNFmAb-CHP was merely 28.2% that of TNFmAb within 96 h post-injection (Fig. 6d), suggesting that CHP-conjugation may significantly reduce the systemic

Original Figs. 6b–d

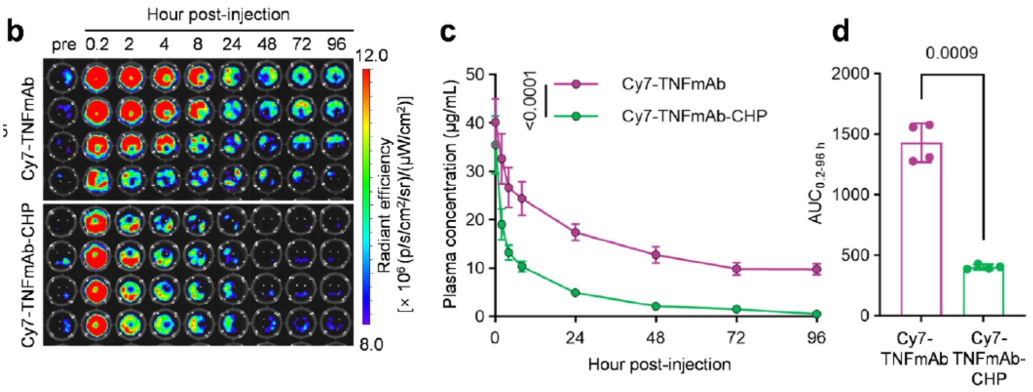

Fig. b Fluorescence intensity of the Cy7-TNFmAb or Cy7-TNFmAb-CHP containing plasma (50 µL/well) at different time points. **c** Quantification of the Cy7-TNFmAb and Cy7-TNFmAb-CHP concentrations in the plasma of the CAIA mice in (**b**). **d** Areas under the curve (AUC) of the plasma concentration-time curves for Cy7-TNFmAb and Cy7-TNFmAb-CHP from 0.2 to 96 h post-injection.

We sincerely thank you for this profound and critical observation. We agree that the initially observed discrepancy, where the serum concentration AUC of Cy7-TNFmAb-CHP was merely 28.2% of that of TNFmAb, was indeed unexpected and inconsistent with the molecules' intended behavior.

Following a systematic investigation and validation process, we discovered that this anomaly was caused by a degree of micro-aggregation in the specific batch of Cy7-TNFmAb-CHP (but not Cy7-TNFmAb) used for the original Fig. 6. This aggregation most likely occurred during several months of storage prior to those specific experiments.

Below is the detailed analysis, the solution implemented, and the updated data.

(1) Evidence of Cy7-TNFmAb-CHP conjugate aggregation

- *Dynamic Light Scattering (DLS) Analysis:* We performed size analysis on the clear sample solutions at 37 °C. The results showed that while Cy7-TNFmAb exhibited a single peak at nearly 10 nm, the Cy7-TNFmAb-CHP batch showed a new peak at 100 nm in addition to the primary 10 nm peak. This confirms the presence of partial antibody aggregation.

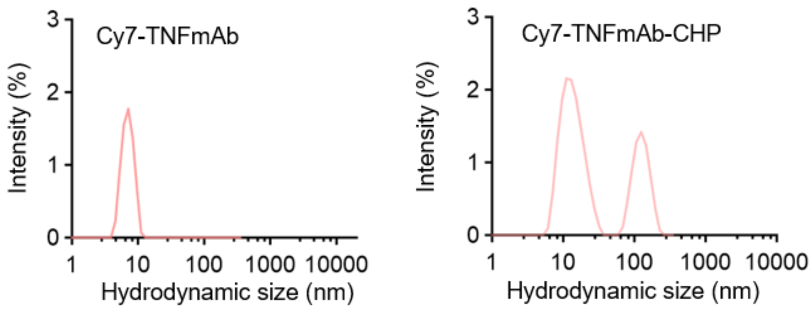

DLS analysis confirmed partial aggregation of Cy7-TNFmAb-CHP compared to Cy7-TNFmAb. (Both samples were measured in PBS at a concentration of 25 μg/mL at 37 °C.)

- *Impact on pharmacokinetics:* Protein aggregates are known to be cleared more rapidly from the bloodstream by the reticuloendothelial system, which significantly shortens their half-life. This explains why the Cy7-TNFmAb-CHP signal in the original Fig. 6 declined much faster than Cy7-TNFmAb, leading to the low AUC ratio.
- *Reversibility:* DLS measurements demonstrated that heating the affected Cy7-TNFmAb-CHP solution to 55 °C for 5 minutes caused the 100 nm peak to disappear. This indicates that the aggregation was reversible under mild thermal treatment.

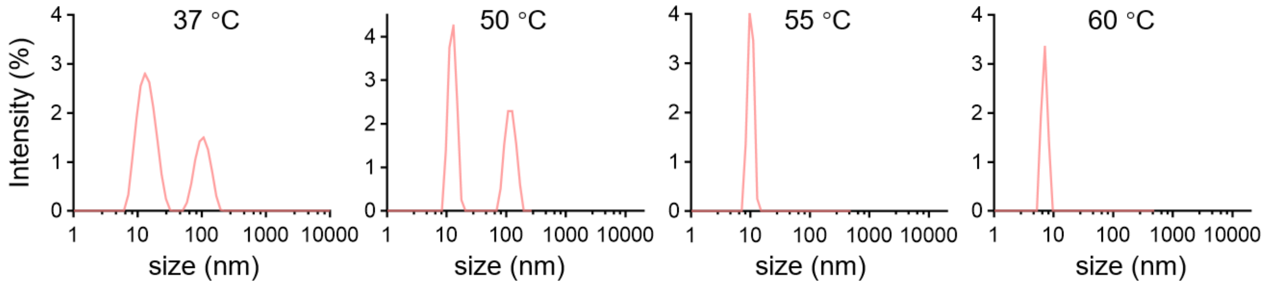

DLS measurements of Cy7-TNFmAb-CHP particle size after heating at different temperatures for 5 min showed that disaggregation of Cy7-TNFmAb-CHP aggregates began upon heating at 55 °C for 5 min. All samples were measured in PBS at a concentration of 25 μg/mL.

(2) Scope of the issue

Crucially, the unlabeled TNFmAb and TNFmAb-CHP used for all therapeutic efficacy experiments (e.g., Fig. 4) did *not* undergo aggregation. Their experimental results remain valid and unaffected. The issue was strictly confined to the *fluorescently labeled* batches used for the metabolic and imaging studies (Figs. 3 and 6).

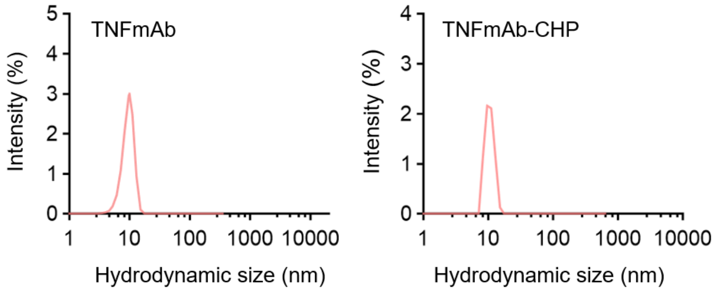

DLS analysis confirmed the absence of aggregation for unlabeled Cy7-TNFmAb and Cy7-TNFmAb-CHP. Both samples were measured in PBS at a concentration of 25 μg/mL and a temperature of 37°C.

(3) Data rectification and updated results

Based on these findings, we have repeated and re-collected all in vivo experimental data involving Cy7-TNFmAb-CHP.

We synthesized a fresh batch of Cy7-TNFmAb-CHP and confirmed the absence of protein aggregation via DLS.

In CAIA mice, the new batch of Cy7-TNFmAb-CHP exhibited a serum concentration trend similar to that of Cy7-TNFmAb (see New Figs. 6b–d). The AUC ratio improved significantly from 28.2% to 85.4%.

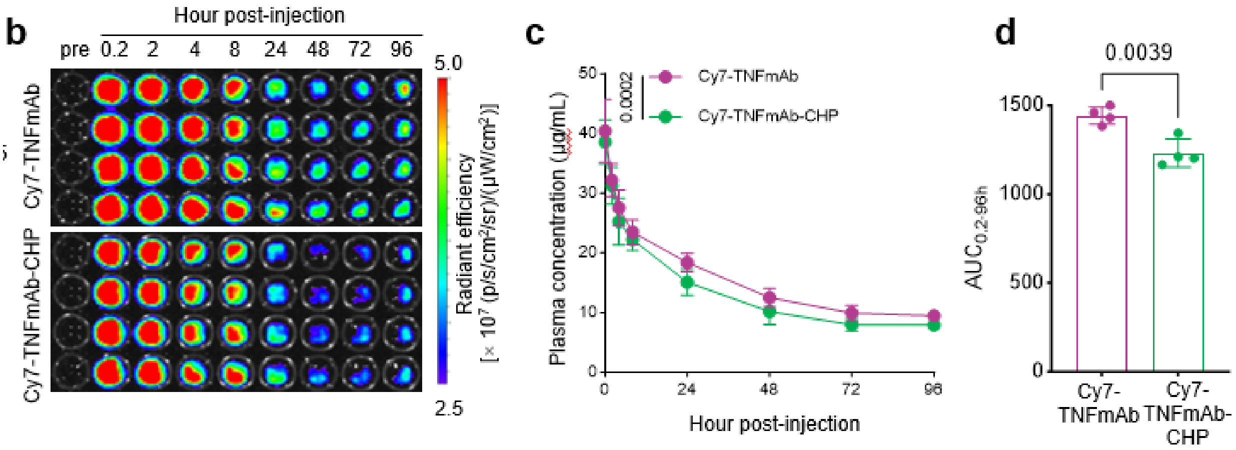

(updated) Fig 6 b Fluorescence intensity of the Cy7-TNFmAb or Cy7-TNFmAb-CHP containing plasma (50 µL/well) at different time points. **c** Quantification of the Cy7-TNFmAb and Cy7-TNFmAb-CHP concentrations in the plasma of the CAIA mice in (**b**). **d** Areas under the curve (AUC) of the plasma concentration-time curves for Cy7-TNFmAb and Cy7-TNFmAb-CHP from 0.2 to 96 h post-injection.

This confirms that the initial low AUC was due to the rapid clearance of aggregates, rather than an inherent, unexpected change in the conjugate's properties.

We have also updated the biodistribution data in **Figs. 6e & 6f** and the in vivo targeting data in **Figs. 3f & 3g** using the new batch of Cy7-TNFmAb-CHP conjugate. Detailed responses regarding these updates can be found in our replies to Comments 9 and 10 below.

(4) Pharmacokinetics data in healthy mice

Regarding your question, *"Is there PK data available for the 2 compounds in healthy mice?"*, we have performed the requested experiments.

The results show that the plasma concentration curves for Cy7-TNFmAb and Cy7-TNFmAb-CHP are even more closely aligned in normal control mice, with no significant difference in the AUC. These data have been added to the Supplementary Information.

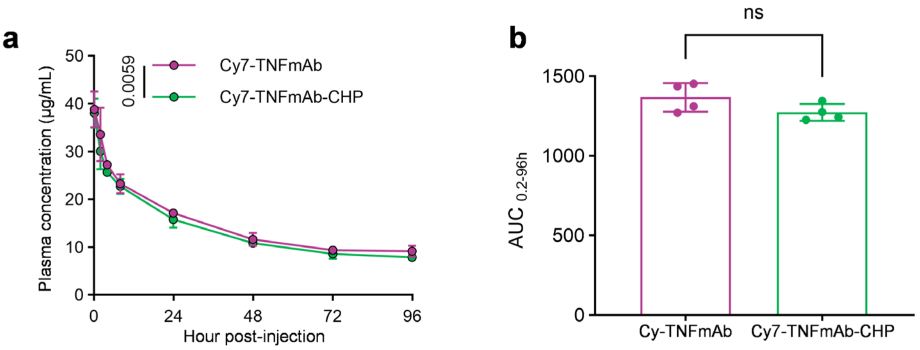

Supplementary Fig. 31. a Fluorescence based quantification of Cy7-TNFmAb and Cy7-TNFmAb-CHP concentrations in the plasma of NC mice at different time points post i.v. injection (dose: 30 µg). **b** AUC of the plasma concentration-time curves for Cy7-TNFmAb and Cy7-TNFmAb-CHP from 0.2 to 96 h post-injection. Statistical analysis: paired *t*-test (**a**) and unpaired *t*-test (**b**). ns: no significance.

In addition to updating the figures, we have revised the corresponding figure descriptions, results and discussions in the manuscript to reflect these corrected findings.

Results:

Pharmacokinetics and biodistribution of Cy7-TNFmAb-CHP in CAIA mice

Finally, we characterized the longitudinal blood concentrations of Cy7-TNFmAb-CHP and Cy7-TNFmAb via Cy7 fluorescence measurements of serial blood samples collected from CAIA mice 0.2–96 h post i.v. injection (Fig. 6a, b). Both probes exhibited a similar gradual decline, approaching a plateau by 96 h (Fig. 6b, c). At equivalent dosing, the area under the concentration-time curve for Cy7-TNFmAb-CHP was approximately 85.4% of that observed for TNFmAb within 96 h post-injection (Fig. 6d). Interestingly, the pharmacokinetic profiles of the two constructs were nearly indistinguishable in healthy control mice (Supplementary Fig. 31). This suggests that the modest reduction in serum concentration observed for Cy7-TNFmAb-CHP in CAIA mice (Fig. 6c, d) may be partially attributable to its enhanced retention within inflamed RA joints.

Updates in Discussion:

the superior efficacy of TNFmAb-CHP[56]. Furthermore, our study demonstrates the unique utility of light-sheet microscopy in visualizing the in vivo spatial distribution of therapeutics across tissue architectures[64]. While CHP conjugation led to a modest reduction in systemic exposure (Fig. 6d), it facilitated a preferential accumulation of TNFmAb within the inflamed ECM of RA joints (Fig. 6f). This subtle shift in biodistribution suggests a promising strategy for improving the therapeutic index and reducing the potential for systemic side effects (Fig. 6). We also provide preliminary evidence that the collagen-hybridizing targeting strategy can be applied to different RA animal models and possesses potential for clinical translation (Supplementary Figs. 29-30).

9. The authors should clarify the clearance route of the compounds Cy7-TNFmAb and Cy7-TNFmAb-CHP: is it primarily renal? What are the uptake differences in feces/bowels and urine? The marked difference observed in blood levels does not appear to be reflected in other organ systems; this discrepancy cannot be fully explained by the differences observed in the front paws alone. (Fig.6)

Original Figs. 6e & 6f

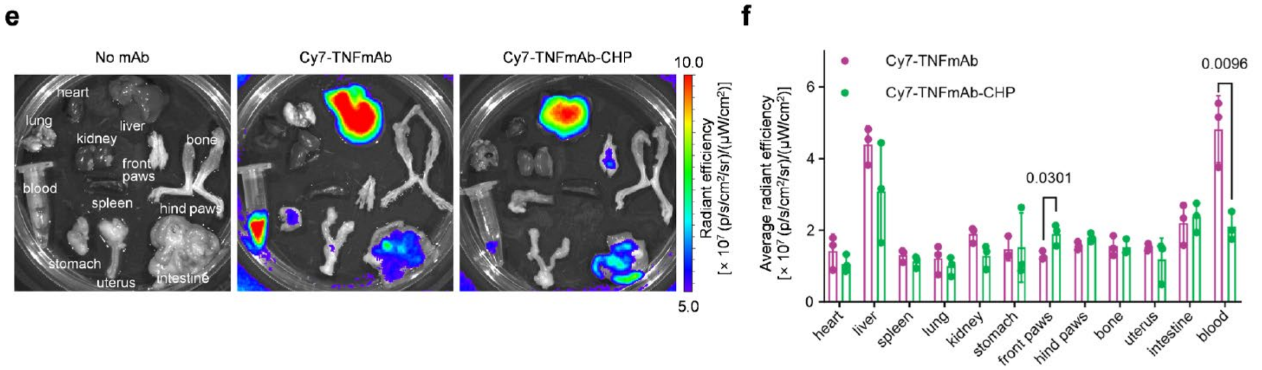

Fig. 6e Representative fluorescence images of the hearts, livers, spleens, lungs, kidneys, stomachs, intestines, uteruses, paws, and blood of CAIA mice harvested with no mAb injection (No mAb, *n* = 1 mouse) or 24 h post i.v. injection of 30 μg of Cy7-TNFmAb or Cy7-TNFmAb-CHP (average total arthritis score per mouse: 5.5, *n* = 3 mice). **f** Average radiant efficiency of each organ quantified from the fluorescence images in (**e**).

We sincerely appreciate your insightful comments regarding the clearance pathways and the consistency of our biodistribution data.

We agree that in the original manuscript, the marked difference in blood levels was indeed inconsistent with the similar signal levels observed in other organ systems in Figs. 6e and 6f, and that this discrepancy could not be fully explained by the differences in the front paws alone.

However, as detailed in our response to the previous comment, this anomaly was primarily due to the micro-aggregation of the initial batch of Cy7-TNFmAb-CHP. With the new data collected using a non-aggregated batch, the serum concentration profile of Cy7-TNFmAb-CHP is now consistent with that of Cy7-TNFmAb, eliminating the marked difference previously observed.

In addition, we have re-conducted the biodistribution and clearance experiments (tissues harvested at 24 h post-injection). The updated results and our analysis are as follows:

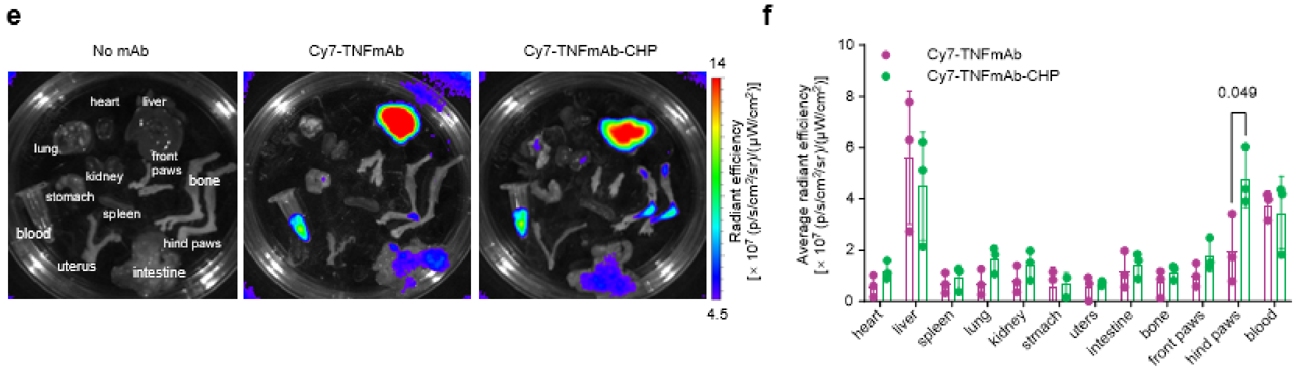

(updated) Fig. 6 e Representative fluorescence images of the major organs, paws, and blood of CAIA mice harvested with no mAb injection (No mAb, *n* = 1 mouse) or 24 h post i.v. injection of 30 μg of Cy7-TNFmAb or Cy7-TNFmAb-CHP (average total arthritis score per mouse: 7, *n* = 3 mice). **f** Average radiant efficiency of each organ quantified from the fluorescence images in (**e**).

Compared to the original data (which was affected by the rapid clearance of aggregated proteins), the new data show:

- The Cy7-TNFmAb-CHP signal in the liver is now much closer to that of Cy7-TNFmAb.

- The signal in the inflamed joints for Cy7-TNFmAb-CHP is significantly more pronounced than in the previous version, reflecting its true targeting capability.

We have also included additional biodistribution data in CAIA mice at 2 h post-injection in the Supplementary Information.

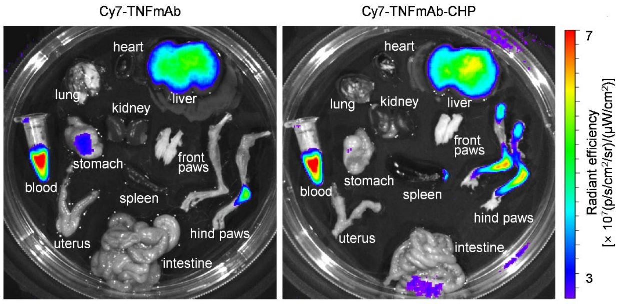

Supplementary Fig. 32 Representative fluorescence images of the major organs, paws, and blood of CAIA mice harvested 2 h post i.v. injection of 30 μg of Cy7-TNFmAb or Cy7-TNFmAb-CHP.

Both the 2 h and 24 h data demonstrate that:

- The biodistribution patterns of Cy7-TNFmAb-CHP and Cy7-TNFmAb across all major organs are very similar.
- Both compounds are primarily cleared through the liver (hepatic clearance) rather than the renal route.

Finally, in addition to updating the figures (Fig. 6), we have thoroughly revised the Results and Discussion sections to accurately reflect these findings and clarify the clearance mechanisms.

Results:

We further assessed the biodistribution of Cy7-TNFmAb-CHP and Cy7-TNFmAb in major organs of CAIA mice 24 h post-injection via fluorescence imaging (Fig. 6e). Aside from the inflamed paws, the Cy7 fluorescence intensities from Cy7-TNFmAb-CHP and Cy7-TNFmAb were comparable across all harvested organs and tissues (Fig. 6f). Similar biodistribution profiles were observed at 2 h post-injection (Supplementary Fig. 32).

To investigate the in vivo toxicity of the TNFmAb-CHP conjugate, we also analyzed the lungs, livers, spleens, kidneys, and hearts of all the mice at the endpoint of the efficacy study (Fig. 4a). Histology of the organs from the TNFmAb-CHP-treated mice showed intact cellular and tissue architecture without apparent inflammation, hemorrhage, or necrosis, comparable to that of the TNFmAb and normal groups (Supplementary Fig. 33). Collectively, these data suggest that CHP conjugation enhances the therapeutic index of TNFmAb by facilitating targeted joint retention without significantly altering systemic clearance or inducing off-target organ toxicity.

Updates in Discussion

the superior efficacy of TNFmAb-CHP[56]. Furthermore, our study demonstrates the unique utility of light-sheet microscopy in visualizing the in vivo spatial distribution of therapeutics across tissue architectures[64]. While CHP conjugation led to a modest reduction in systemic exposure (Fig. 6d), it facilitated a preferential accumulation of TNFmAb within the inflamed ECM of RA joints (Fig. 6f). This subtle shift in biodistribution suggests a promising strategy for improving the therapeutic index and reducing the potential for systemic side effects (Fig. 6). We also provide preliminary evidence that the collagen-hybridizing targeting strategy can be applied to different RA animal models and possesses potential for clinical translation (Supplementary Figs. 29-30).

10. The data appear to show a similar decline between the 2 variants. Also, no clear plateau is reached, which may instead suggest slower clearance of the CHP–TNF mAb from circulation rather than enhanced or specific uptake compared to TNF mAb. (Fig. 3g, Suppl.Fig.13 b)

Original Fig. 3g

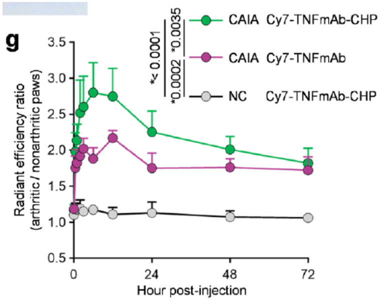

Fig 3 g Changes in radiant efficiency ratio of the arthritic paws (right hind) to the non-arthritic paw (left hind) in the normal and CAIA mice (n = 3 mice).

Original Supplementary Fig. 13b,c

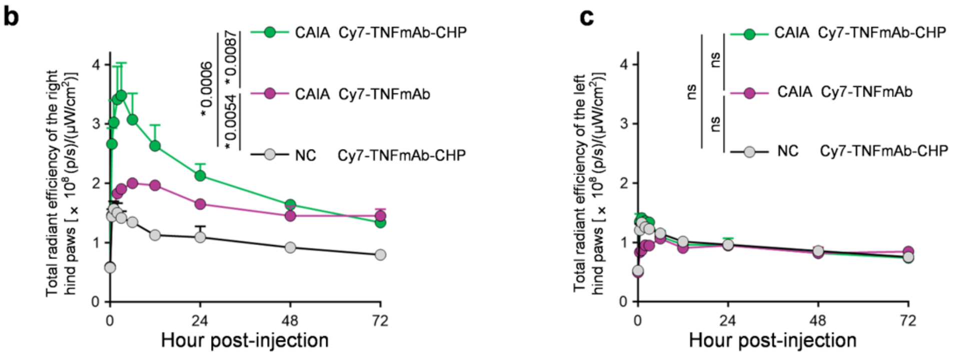

Quantified in vivo Cy7 fluorescence signals from the right (b) and left (c) hind paws of the normal and CAIA mice studied in Fig. 3f from 0 to 72 h post-injection of Cy7-TNFmAb-CHP or Cy7-TNFmAb. Numbers were expressed as mean + standard deviation. Statistical analysis: one-way repeated measures ANOVA with Geisser-Greenhouse correction for sphericity and Tukey's multiple comparisons (b, c). ns: no significance.

We sincerely thank you for this insightful comment.

We have repeated the in vivo targeting experiments in Fig. 3 using a new, non-aggregated batch of Cy7-TNFmAb-CHP. The updated data addresses the concerns regarding the signal decline and the specificity of uptake.

The initial data in the original Fig. 3f showed that the signal of the previous Cy7-TNFmAb-CHP batch in RA paws nearly disappeared by 48 h.

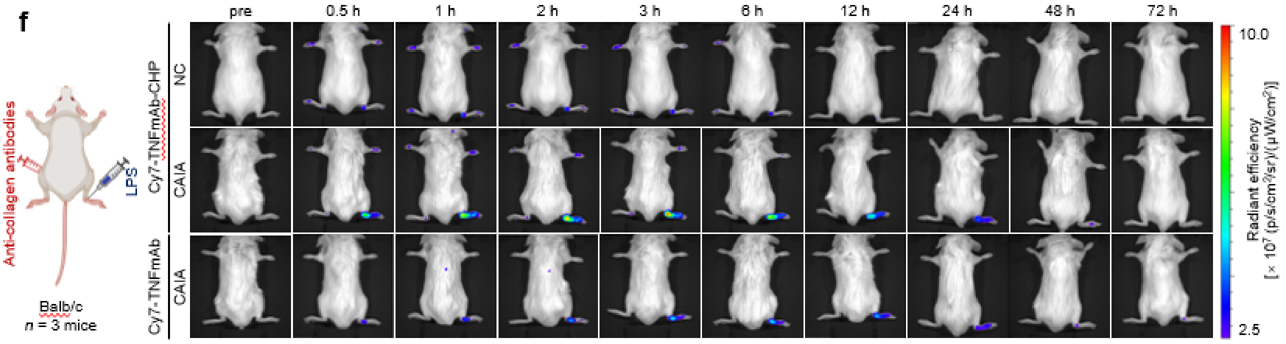

However, our updated experiments demonstrate a significant improvement:

- Extended retention: The signal of the new Cy7-TNFmAb-CHP in inflamed joints now persists for at least 72 h (New Fig. 3f).

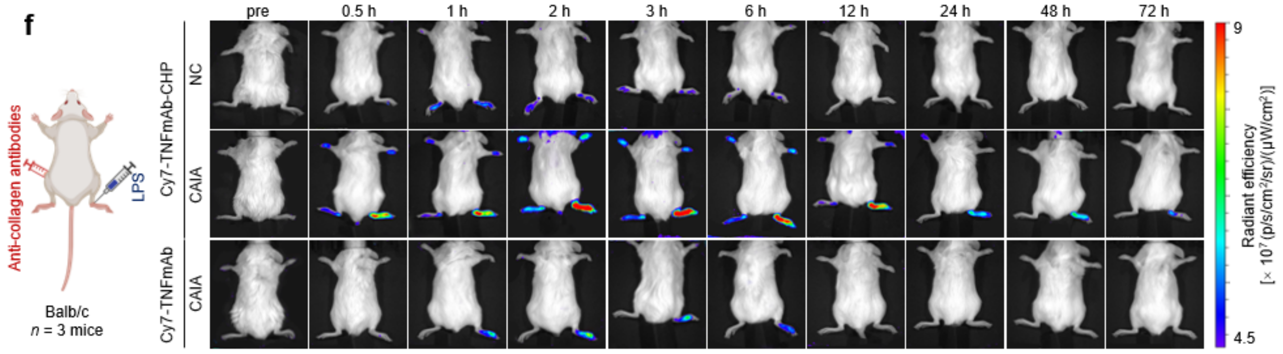

- The updated Fig. 3g and Supplementary Fig. 13b (now Supplementary Fig. 18 c–d) now show a clear divergence between the two variants. The signal intensity of Cy7-TNFmAb-CHP in RA paws, as well as the ratio of RA-to-NC paw signals, consistently remains approximately two-fold higher than that of Cy7-TNFmAb throughout the observation period.

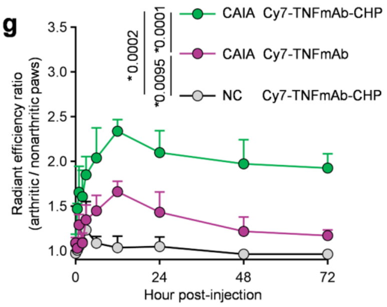

(updated) Fig 3 g Changes in radiant efficiency ratio of the arthritic paws (right hind) to the non-arthritic paw (left hind) in the normal and CAIA mice (n = 3 mice).

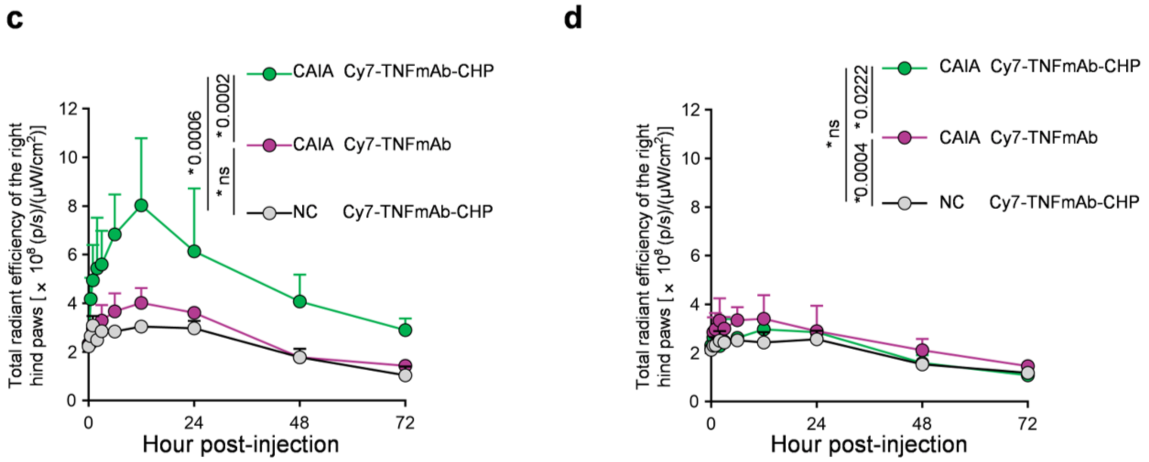

Updated Supplementary Fig. 18 (original Fig. 13). c-d Quantified in vivo Cy7 fluorescence signals from the right (**b**) and left (**c**) hind paws of the normal and CAIA mice studied in Fig. 3f from 0 to 72 h post-injection of Cy7-TNFmAb-CHP or Cy7-TNFmAb. Numbers were expressed as mean + standard deviation. Statistical analysis: one-way repeated measures ANOVA with Geisser-Greenhouse correction for sphericity and Tukey's multiple comparisons (**b**, **c**). ns: no significance.

These results resolve the concern that the two variants showed a "similar decline" and confirm the enhanced targeting capability of the CHP-conjugated antibody.

Regarding the observation that no clear plateau is reached, we appreciate your perspective that this might suggest slower circulation clearance. However, we respectfully suggest an alternative interpretation based on the unique targeting mechanism of our conjugate. (i) Unlike typical drugs targeting intracellular or cell-surface receptors, Cy7-TNFmAb-CHP targets denatured collagen via triple-helix hybridization. This hybridization is a non-covalent interaction. (ii) Furthermore, damaged collagen is located in the extracellular matrix and is subject to continuous physiological or pathological remodeling. Therefore, even with specific binding, the conjugate is not necessarily sequestered permanently within the tissue. As the systemic blood concentration gradually decreases, a

corresponding decline in the joint signal is expected. (iii) Nonetheless, in the new Fig. 3g, the signal intensity of Cy7-TNFmAb-CHP relative to the contralateral normal paw (the RA/NC ratio) does indeed approach a plateau after 48 hours, suggesting that the specific accumulation in the lesion site stabilizes relative to non-specific perfusion.

In light of these new findings, we have updated the data in Fig. 3 and Supplementary Fig. 18, and have revised the Results sections.

To test the localization of the conjugate to the inflamed joint in vivo, we injected LPS selectively at the right hind footpad (Fig. 3f). This local LPS injection induced severe arthritis at the right hind paw compared to the left one (Supplementary Fig. 18a). On day 1 post-LPS injection, an equivalent dose of Cy7-labeled TNFmAb-CHP or TNFmAb was intravenously injected into this modified CAIA model and the normal controls, which were subjected to frequent near-infrared fluorescence imaging in 3 days (Fig. 3f). The Cy7-labeled TNFmAb-CHP rapidly accumulated in the inflamed right ankle from 0.5 to 3 h post-injection, whereas the Cy7-TNFmAb showed only weak signals (Fig. 3f & 3g, Supplementary Fig. 18); from 12 to 72 h post-injection the TNFmAb-CHP fluorescence declined slowly in the inflamed ankle compared to the TNFmAb (Fig. 3f, Supplementary Figs. 18c & d). The ratio of the Cy7 fluorescence level in the arthritic paw to the non-arthritic paw was significantly higher in mice injected with TNFmAb-CHP than with TNFmAb (Fig. 3g). Analysis of the area under the curve (AUC) for the fluorescence signal over 72 h revealed that the ratio of mAb uptake in arthritic versus non-arthritic paws was 3.1-fold higher for TNFmAb-CHP than for the parent TNFmAb (Supplementary Figs. 18b). These data demonstrated the CHP's capacity to deliver its mAb conjugate to the arthritic ankle joint in vivo.

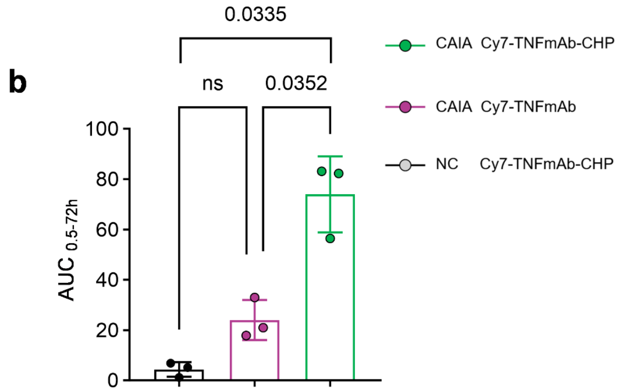

New Supplementary Fig. 18. b Areas under the curve (AUC) of the radiant efficiency ratio of the arthritic paws (right hind) to the non-arthritic paws for Cy7-TNFmAb and Cy7-TNFmAb-CHP from 0.5 to 72 h post-injection in Fig. 3g (baseline ratio: 1.0).

Minor comments:

The revised text or response corresponding to each minor comment is presented below.

• Introduction:

No comment

• Results:

Line 88: swollen swelling

> cartilage degradation and bone erosion (Supplementary Fig. 1b)[39]. Two hours post intravenous (i.v.) injection of Cy7-labeled CHP, drastically stronger in vivo near-infrared fluorescence signals were detected in the swollen ankle regions in the hind paws of the CAIA mice (6 days post-disease onset, hind paw arthritis score = 4) than those of the normal naïve mice (Fig. 1a and Supplementary Fig. 2).

Line 108: in contrast compared with (would fit better as no superlative form is used, and considering the important difference between the two collagen signals)

> CHP fluorescence signals preferentially colocalized with collagen IV immunostaining rather than collagen I (Supplementary Fig. 6). The absence of binding for the sequence-scrambled control (Cy3-

Line 109: "immunofluorescently" not commonly used in scientific writing, consider a more standard phrasing (by/with/using immunofluorescence)

Line 109: "upregulated usually refers to a baseline reference (upregulated compared to unstimulated, healthy control). Other phrasing such as high expression, abundance, prevalence, would be more suitable

> further supported by the competitive blocking experiments (Supplementary Fig. 7). Moreover, immunofluorescence analysis revealed prevalence of MMP1, 2, 3, 9, 13, 14 (esp. MMP1, 3, 9, and 13) and CTSK in the pannus cryosections (Fig. 1d) surrounded by the Cy3-CHP-marked denatured collagen (Supplementary Fig. 8), suggesting extensive matrix degradation. Strikingly, Cy3-CHP

Overall note about this part (line 106-116): co-localization can be mentioned here; any quantitative data would strengthen the interpretation and clarity of the findings (Fluorescence intensity; co-localization of signals).

We thank the reviewer for this suggestion. We carefully considered performing a quantitative co-localization analysis between Cy7-CHP and the immunofluorescence signals of various proteases (e.g., MMPs and CTSK). However, we have opted not to include this analysis for the following scientific reasons:

- Most MMPs and Cathepsin K are secreted proteases. Their spatial localization, as detected by immunofluorescence, indicates the presence of the enzyme proteins but does not necessarily reflect their enzymatic activity. These enzymes are often secreted as inactive pro-enzymes or are rapidly sequestered by endogenous inhibitors (such as TIMPs) after secretion. Therefore, a high degree of physical co-localization does not directly correlate with the site of collagen proteolytic cleavage.
- Due to the rapidly diffusing nature of these secreted proteases within the extracellular matrix, the location of the enzyme at the time of tissue fixation may not perfectly align with the site where it previously denatured the collagen. Cy7-CHP serves as a historical record of accumulated collagen damage, whereas immunofluorescence provides a snapshot of protein presence, making a precise pixel-by-pixel co-localization analysis (e.g., Pearson's correlation) potentially misleading.

- The primary objective of our study is to utilize CHP as a specific marker for the *consequence* of enzymatic activity, namely, denatured collagen, rather than to identify the specific contribution of individual proteases to that damage. We believe that characterizing the overall state of collagen degradation provides a more accurate assessment of disease progression than attempting to correlate it with specific secreted proteins that may no longer be active at that specific site.

Given these biological complexities, we believe that quantitative co-localization would not reliably strengthen the interpretation of our findings and could inadvertently introduce a false sense of spatial precision regarding enzyme-substrate interaction.

Line 222: not only does not

Line 223: “confers the mAb an affinity” requires English rephrasing; Does it mean that the affinity of CHP–anti-TNF is higher than that of anti-TNF alone?

The corresponding text has been revised to clarify that the conjugate’s affinity for TNF-α remains unaltered.

tissues compared to the RA pannus (Supplementary Fig. 17b). Collectively, these in vitro and histological results demonstrate that the CHP conjugation preserves the intrinsic antigen-recognition capability of TNFmAb while endowing the conjugate a second affinity for the denatured collagen prevalent in the RA joint matrix.

Line 237: “1.5 times greater” data not shown. AUC graph could be added to the Suppl. Fig. 13.

The corresponding AUC graph has been added to Supplementary Fig. 18b to provide quantitative support for this comparison.

in mice injected with TNFmAb-CHP than with TNFmAb (Fig. 3g). Analysis of the area under the curve (AUC) for the fluorescence signal over 72 h revealed that the ratio of mAb uptake in arthritic versus non-arthritic paws was 3.1-fold higher for TNFmAb-CHP than for the parent TNFmAb (Supplementary Figs. 18b). These data demonstrated the CHP’s capacity to deliver its mAb conjugate to the arthritic ankle joint in vivo.

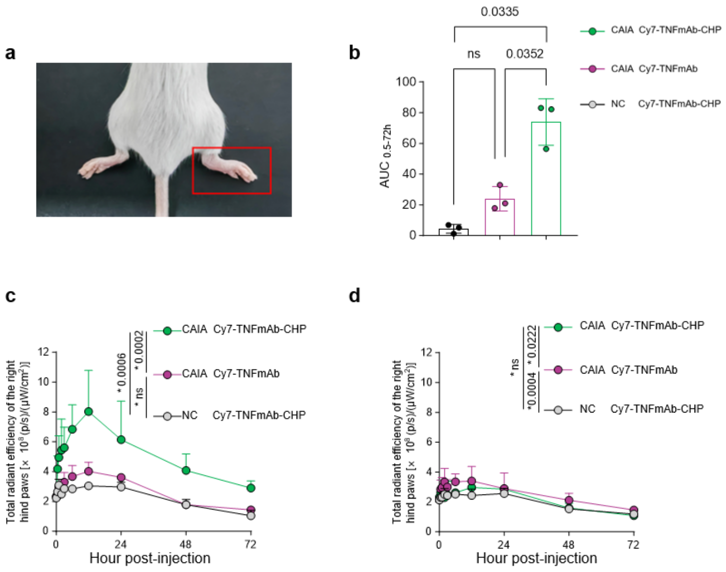

Supplementary Fig. 18. a Appearance of the inflamed arthritic right hind paw (red box) of a CAIA mouse triggered with local LPS injection (Fig. 3f). **b** Areas under the curve (AUC) of the radiant efficiency ratio of the arthritic paws (right hind) to the non-arthritic paws for Cy7-TNFmAb and Cy7-TNFmAb-CHP from 0.5 to 72 h post-injection in Fig. 3g (baseline ratio: 1.0). **c-d** Quantified in vivo Cy7 fluorescence signals from the right (**c**) and left (**d**) hind paws of the normal and

CAIA mice studied in Figure 3f from 0 to 72 h post-injection of Cy7-TNFmAb-CHP or Cy7-TNFmAb. Numbers were expressed as mean + standard deviation. Statistical analysis: Welch's ANOVA with post hoc Dunnett's T3 test (b), one-way repeated measures ANOVA with Geisser-Greenhouse correction for sphericity and Tukey's multiple comparisons (**b**, **c**). ns: no significance.

Line 271: Why a p-value is reported in this figure but not in the others (inconsistency in reporting statistical presentation).

We have removed all p-values from the main text for consistency.

(Supplementary Fig. 22). On day 7 post-mAb-treatment, in vivo, near-infrared fluorescence imaging using Cy7-CHP revealed a marked reduction in collagen degradation and denaturation in the hind paws treated with TNFmAb-CHP than those treated with TNFmAb (Fig. 4d & 4e, Supplementary Fig. 23);

Line 274: The description of the histological changes could be more specific.

a similar trend was also noted in the front paws (Supplementary Fig. 23). Endpoint histological examination and scoring of the ankle joints revealed that synovial hyperplasia, inflammatory cell infiltration, and articular cartilage erosion were effectively attenuated by TNFmAb-CHP compared to TNFmAb (Fig. 4f & 4g). Furthermore, immunostaining revealed an increase in anti-inflammatory IL-

Line 323/324: The phrasing in this section is too vague; please provide more detailed information when comparing with the control paw.

within the inflamed interphalangeal joints (Fig. 5f and b, arrowheads). Meanwhile, we noted that the spatial distribution of Cy7-TNFmAb-CHP administered in mice on day 1 post-LPS-induction (arthritis score: 1) was highly similar to the above results (Supplementary Fig. 27), whereas fluorescence was markedly less pronounced in the synovial tissues of the contralateral non-arthritic left hind paws (internal control, Supplementary Fig. 28). These findings demonstrate that CHP selectively anchors

Suppl.Fig.22: This figure would benefit from including inset coronal views, similar to those shown in Suppl.Fig.21/ Fig.5, to allow a more precise and consistent description of the results.

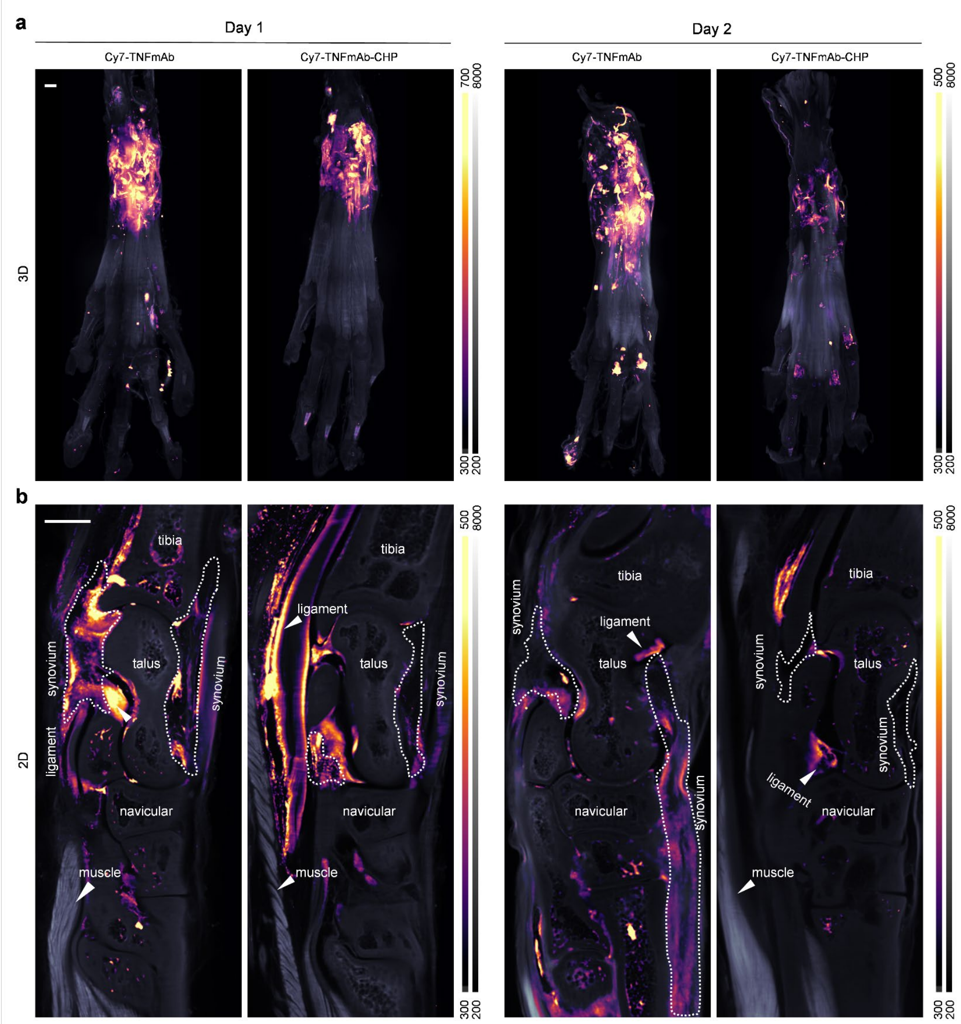

New Supplementary Fig. 28 (originally Supplementary Fig. 22). The 3D holistic views of the contralateral non-arthritic left hind paws from the CAIA mice collected 2 h post i.v. injection of Cy7-TNFmAb-CHP or Cy7-TNFmAb on day 1 or 2 post-LPS-injection in the right hind paws (shown in Supplementary Fig. 27a and Fig. 5a, respectively). 3D holistic view (**a**), 2D sagittal view (**b**). Scale bars: 500 µm.

Line 341/342: "provided with an affinity" requires rephrasing (ref. line 223).

Furthermore, we demonstrated that the CHP peptide selectively binds to the inflamed synovial pannus of a collagen-induced arthritis (CIA) mouse model, as well as to the arthritic shoulder joint cartilage from an RA patient (Supplementary Figs. 29 & 30). Crucially, this collagen hybridization-mediated binding also enabled the TNFmAb-CHP conjugate to adhere to these pathological tissues (Supplementary Figs. 29 & 30). These data implied that our targeting strategy would be effective across different RA models and potentially in human RA.

Line 356: The difference observed in blood in Fig.6f may be highlighted too, or alternatively, mentioned earlier alongside the plasma concentration data to fully describe the blood compartment. Additionally, only the front paws show a significant difference.

Line 466: No significant differences were reported for the listed organs. Could the authors provide an explanation for the differences observed in the front paws but not in the hind paws? PK data could be more discussed here (ref. Fig.6 major comments).

These concerns have been resolved following the update of the complete dataset for Figs. 6e and 6f. The revised data demonstrate that blood concentrations of Cy7-TNFmAb and Cy7-TNFmAb-CHP are

comparable (no significant difference) at 24 h post-injection; however, significant differences in their accumulation within the hind paws are evident. A similar trend in fluorescence uptake was observed in the front paws, although the difference did not reach statistical significance.

We further assessed the biodistribution of Cy7-TNFmAb-CHP and Cy7-TNFmAb in major organs of CAIA mice 24 h post-injection via fluorescence imaging (Fig. 6e). Aside from the inflamed paws, the Cy7 fluorescence intensities from Cy7-TNFmAb-CHP and Cy7-TNFmAb were comparable across all harvested organs and tissues (Fig. 6f). Similar biodistribution profiles were observed at 2 h post-injection (Supplementary Fig. 32).

• Discussion:

Line 421/422: Examples?

more objective and quantitative than arthritis scoring. For future translational applications, we will develop CHP-based imaging agents compatible with clinical modalities extending beyond the near-infrared fluorescence used in this work (e.g., ultrasound and photoacoustic imaging).

• M&M:

Line 683/684: Does the 2nd Ab control contain also Cy3-CHP?

The 2nd Ab control contains the sequence-scrambled Cy3-^{S}CHP as a negative control.

Supplementary Figs. 6 & 8). Briefly, after the OCT was removed, the sections were blocked with 10% goat serum for 30 min and incubated with MMPs and collagen antibodies diluted in pre-heated 10 µM Cy3-CHP (in 1% goat serum) overnight at 4 °C; for the negative control, sections were incubated with 10 µM sequence-scrambled Cy3-^{S}CHP in the absence of primary antibodies at this step. The staining

Reviewer #2 (Remarks to the Author):

I co-reviewed this manuscript with one of the reviewers who provided the listed reports. This is part of the Nature Communications initiative to facilitate training in peer review and to provide appropriate recognition for Early Career Researchers who co-review manuscripts.

Thank you very much.

Reviewer #3 (Remarks to the Author):
In this manuscript, Chen et al report on the use of a CHP peptide for targeted imaging and therapy in mouse models of RA. The overall goal is to reduce off-target toxicity of immunosuppressive therapies by CHP-targeting strategy and improve imaging of collagen breakdown during disease progression and regression.
The authors validate the Cy7-labeled CHP probe in a CAIA mouse model in which they inject the and image the mice 2 hours after i.v. injection. They also use a Cy7-labeled scrambled peptide as control. The Cy7 signal is observed only in the case of CHP-tagged probe and in RA animals, conforming that the probe does not bind to healthy joints nor does the scrambled CHP bind to RA joints. They also use a Cy7-CHP that is modified with lysine in order to localize the Cy7 fluorescence by light sheet imaging. The use of this lysine-modified probe is on order to improve retention during the tissue clearing procedure.
The authors use the probe to track progression of RA and regression when RA mice are treated with DEX by both an arthritis score and the Cy7-CHP signal. The authors then show that CHP tagging enabled localization of mAB to inflamed paw. In these experiments, the authors attach a TNF-alpha mAB (TNFmAB) to CHP and Cy7 and show that this drug accumulated more in the inflamed paw compared to the TNFmAB tagged with Cy7. They further show that CHP conjugation to the mAB enhanced the efficacy of this drug based on the arthritis score, ancle thickness, and Cy7-CHP imaging. Finally, the authors also show the pharmacokinetics of the mAB-CHP conjugates.
Overall the manuscript is well constructed and well written, clear and interesting. The authors also have clear controls, and well-designed experiments. However, there remains some outstanding questions and comments which require address before publication of this manuscript:
Thank you for the favorable comments and constructive suggestions. Please see below our point-by-point responses to individual reviewer comments (in blue).

1. Figure 2h: **(i)** The radiant efficiency values for the normal controls is very different from that reported for normal controls in figure 1. Both distributions are very tight, so why are the values in Figure 2 almost double that in Figure 1?
(ii) Further, why is the distribution in the PBS group so varied? The values for CAIA animals injected with Cy7-CHP in figure 1 are much tighten, with standard deviation being < 1 X 10^8 Total radiant efficiency in hind paw in vivo. Whereas in figure 2h in PBS treated group, which presumably should not be much different than the CAIA shows a standard deviation of ~ 4 X 10^8 Total radiant efficiency. Explain these differences.
(iii) Figure 2H and 2J: why the differences in the statistical analysis?

Original Figs:

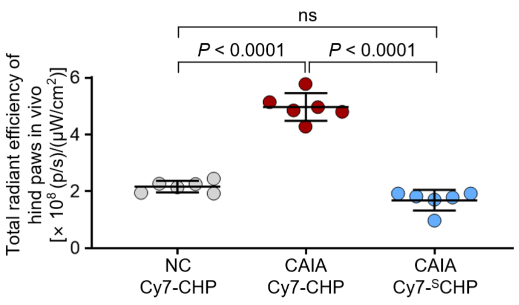

Fig. 1a

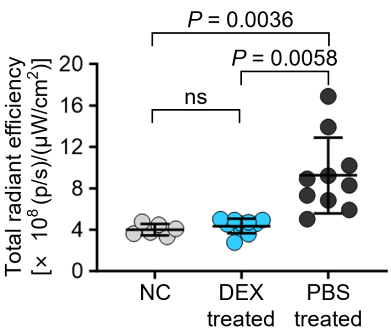

Fig. 2h

Fig. 2 Statistical analysis: Spearman correlation coefficient (**e**), Welch's ANOVA with post hoc Dunnett's T3 test (**h**), and Kruskal-Wallis test with post hoc Dunn's test (**j**).

We sincerely appreciate your meticulous examination of our imaging data and the opportunity to clarify the discrepancies noted between Fig. 1 and Fig. 2.

(i) Difference in radiant efficiency values for the NC.

Regarding the difference in radiant efficiency values for the normal control (NC) groups between Fig. 1 and Fig. 2h, this is primarily attributable to the long-term stability and maintenance of the imaging equipment. The in vivo fluorescence imaging system (IVIS, PerkinElmer) used in this study is a core facility instrument with an extremely high workload, operating for thousands of hours annually. There was an interval of more than one year between the experiments for Fig. 1 and Fig. 2 (the entire research project spanned over four years). During this period, the equipment underwent several routine maintenance sessions and component replacements, which had led to fluctuations in absolute radiant efficiency values across different time points. However, we emphasize that the data within each specific experiment are internally consistent, and the statistical differences between groups remain highly significant and reproducible.

(ii) The higher variance in the PBS-treated CAIA group in Fig. 2h

The higher variance observed in the PBS-treated CAIA group in Fig. 2h compared to the tighter distribution in Fig. 1 stems from the inherent heterogeneity of the CAIA animal model and differences in experimental design. In Fig. 1, we specifically pre-selected mice with a uniform disease severity (hind paw arthritis score = 4) for the experiment to ensure a controlled comparison of probe performance. In contrast, Fig. 2h represents a longitudinal therapeutic study. Mice were randomly assigned to treatment groups at the start, and after 10 days of treatment, the 10 hind paws of the 5 mice in the PBS group naturally exhibited varying degrees of disease progression (hind paw arthritis score = 2-4). This individual variation is a characteristic feature of the CAIA model, as also evidenced by the variance of the arthritis score in Supplementary Fig. 13 (original Supplementary Fig. 9).

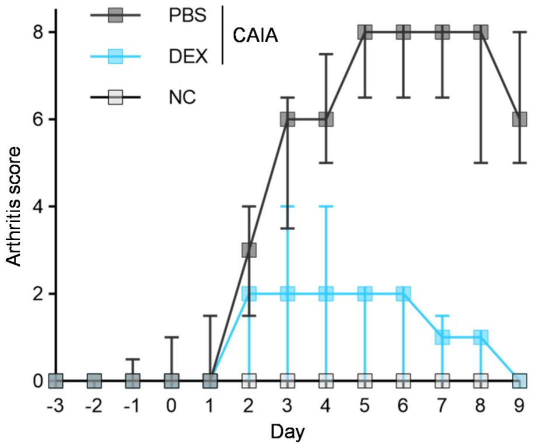

Supplementary Fig. 13 Arthritis scores of the hind paws of the NC mice or the CAIA mice treated with DEX or PBS throughout the study in Fig. 2f. Numbers were expressed as median with interquartile range.

We chose to report the full spectrum of these data points rather than excluding individuals with milder symptoms to maintain objective transparency. We believe this wider distribution actually reflects the reliability and sensitivity of our CHP probe in accurately capturing the actual pathological state of each joint, rather than indicating a lack of reproducibility.

To avoid further confusion, we have added detailed descriptions of the arthritis scores for the mice used in Fig. 1 and Fig. 2h to the revised manuscript.

cartilage degradation and bone erosion (Supplementary Fig. 1b)[39]. Two hours post intravenous (i.v.) injection of Cy7-labeled CHP, drastically stronger in vivo near-infrared fluorescence signals were detected in the swollen ankle regions in the hind paws of the CAIA mice (6 days post-disease onset, hind paw arthritis score = 4) than those of the normal naïve mice (Fig. 1a and Supplementary Fig. 2).

vehicle-only (i.e., PBS buffer) (Supplementary Fig. 13). On day 9 (the endpoint), we i.v. injected the mice with Cy7-CHP; the in vivo Cy7-CHP fluorescence from the hind paws of the DEX-treated mice was almost indistinguishable from the normal controls (Fig. 2g and h) and drastically lower than that of the PBS-treated group (hind paw arthritis scores = 2–4). Histologic analysis of each right hind paw

(iii) The different statistical analyses

Finally, regarding the different statistical analyses used in Fig. 2h and Fig. 2j, we selected the statistical methods based on the specific characteristics of each dataset. We ensured that each test applied was the most appropriate for the underlying distribution of that specific data group to ensure scientific rigor.

For Fig. 2h (fluorescence signal data): The data are continuous and normally distributed, but they show unequal variances (heteroscedasticity). Since the assumptions of traditional ANOVA are not met under these conditions, we used the Welch ANOVA, which is robust to variance heterogeneity. Post-hoc comparisons were performed with Dunnett's T3 test, a method suitable for pairwise testing when variances are unequal and which controls for multiple-comparison error.

For Fig. 2J (arthritis clinical scores): These data represent ranked measurements and do not follow a normal distribution. Therefore, we applied the non-parametric Kruskal–Wallis test for overall group comparison, followed by Dunn's test for post-hoc pairwise comparisons, an appropriate approach for ordinal or skewed data that do not satisfy parametric assumptions.

2. Figure 3: It is not clear to me why Cy7 signal is not observed over the entire mouse body if the injection of the dye was IV. If the antibody without the CHP targeting is non-specific and has off-target effects, why is signal not observed elsewhere?

Original Fig. 3f

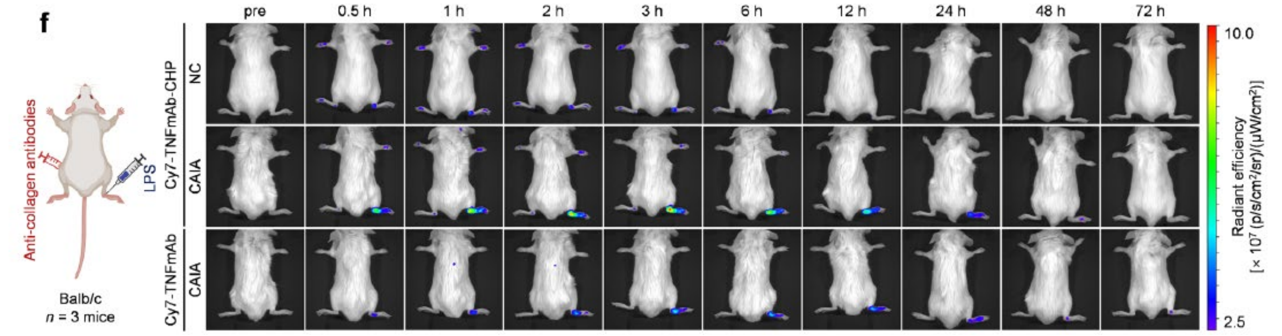

We sincerely thank you for this insightful question regarding the systemic distribution of our probes. We understand the expectation that an intravenous (i.v.) injection might result in a more uniform glow across the body; however, the biodistribution of large-protein therapeutics like full-length antibodies is fundamentally different from that of small-molecule dyes or peptides (e.g., see Supplementary Fig. 2a, 2 h images).

Below is our detailed explanation for the observed imaging patterns:

(1) Unlike small-molecule drugs that diffuse rapidly through tissues, the distribution of full-length antibodies is primarily governed by vascular permeability, target-mediated disposition, and organ-specific uptake (e.g., the liver). Post-injection, the signal does not diffuse uniformly but rather concentrates in areas with high blood perfusion or specific target expression. While a faint systemic signal might be detectable if the imaging threshold were significantly lowered, it would remain negligible compared to the high-intensity signals observed at the target sites (the inflamed paws).

(2) Technically, it is also worth noting that mouse fur significantly attenuates and scatters fluorescence signals. The trunk of the mouse is covered in dense hair, which acts as a physical barrier to any weak Cy7 signal. In contrast, the paws are virtually hairless, allowing for much more sensitive and prominent detection of the fluorescent signal, which further emphasizes the contrast between the target site and the rest of the body.

(3) We would like to clarify that the TNFmAb (without CHP) is not a non-specific or off-target molecule. It is a monoclonal antibody with a high affinity for TNF-α, a cytokine that is heavily upregulated in the inflamed joints of CAIA mice. (Its side effect in RA patents is also not because of its non-specificity, but because it may target other non-joint areas where TNF-α is high, e.g., infected lungs.)

For TNFmAb: Even without the CHP modification, the antibody naturally accumulates in the inflamed paws through specific antigen-antibody interactions (as seen in Fig. 3f).

For TNFmAb-CHP: The addition of CHP creates a dual-targeting strategy. It maintains the original TNF-α targeting capability while adding to the conjugate a second layer of affinity for denatured collagen (such as in the degraded pannus and articular cartilage). The goal of CHP conjugation is to enhance the accumulation and retention of the antibody within the pathological matrix of the joint, rather than fundamentally altering its systemic distribution pattern. The observed signal in Fig. 3 reflects this localized enrichment rather than a systemic failure of the probe to circulate.

3. The data shows that even upon binding, the CHP derivatives washout from the target tissue within 24 hours, meaning that either binding is weak or that the bound complex is cleared from the body. Given this, the authors developed Lys-featuring CHP sequence to enhance retention, since this lysine can react with formaldehyde and neighboring protein lysines to cross-link the probe. However, this is lysine-modified derivative is a *new* and different compound. Why did the authors not use this lysine-containing probe for all studies? How does the performance of this probe compare to the non-lysine-containing probe? How does the blood half life and tissue half-life differ in these two probes? These comparisons are crucial to ensure that the introduction of this lysine does not change the pharmacokinetics and distribution of the probe.

Original text (lines 96–99)

To track the exact anatomic location of CHP binding in the inflamed ankles, we used light-sheet fluorescence microscopy to scan the mice's hind paws, which were dissected 2 h post i.v. injection of a Lys-modified Cy7-CHP [Cy7-Ahx-K-Ahx-(GfO)$_9$] followed by tissue clearing using the PEGASOS method[40] (Fig. 1b, Supplementary Fig. 4, see methods). Strong smoke-like Cy7

Original Fig. 1b

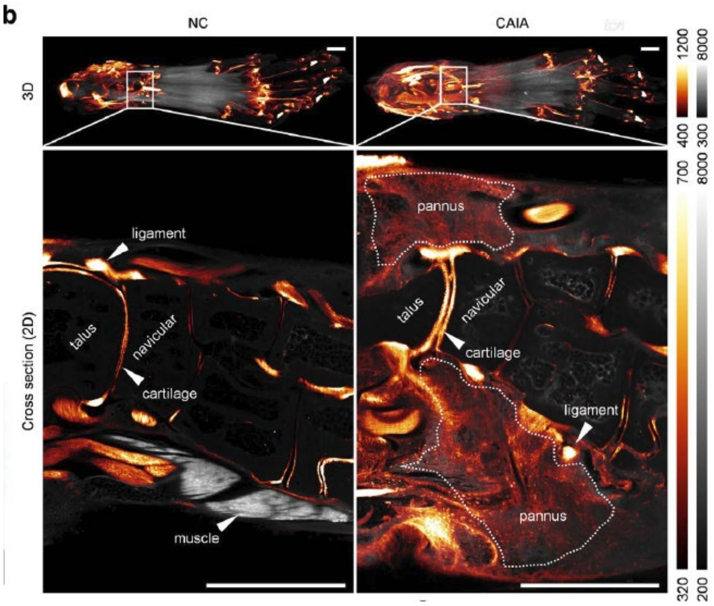

Fig. 1b Representative light-sheet fluorescence microscopy images of the hind paws from the normal and CAIA mice collected and cleared 2 h post i.v. injection of Cy7-CHP.

We sincerely appreciate your concern regarding the potential physiological differences between the original Cy7-CHP [Cy7-Ahx-(GfO)$_9$] and the ***Lys-modified*** Cy7-CHP [Cy7-Ahx-K-Ahx-(GfO)$_9$]. Ensuring that the introduction of a lysine residue does not alter the probe's fundamental biological behavior is indeed critical for the rigor of this study.

To address this, we conducted a systematic comparison of the two probes, evaluating their affinity for denatured collagen *in vitro*, as well as their pharmacokinetics, tissue enrichment, and biodistribution *in vivo* using the CAIA mouse model. These results are now summarized in the New **Supplementary Fig. 5**.

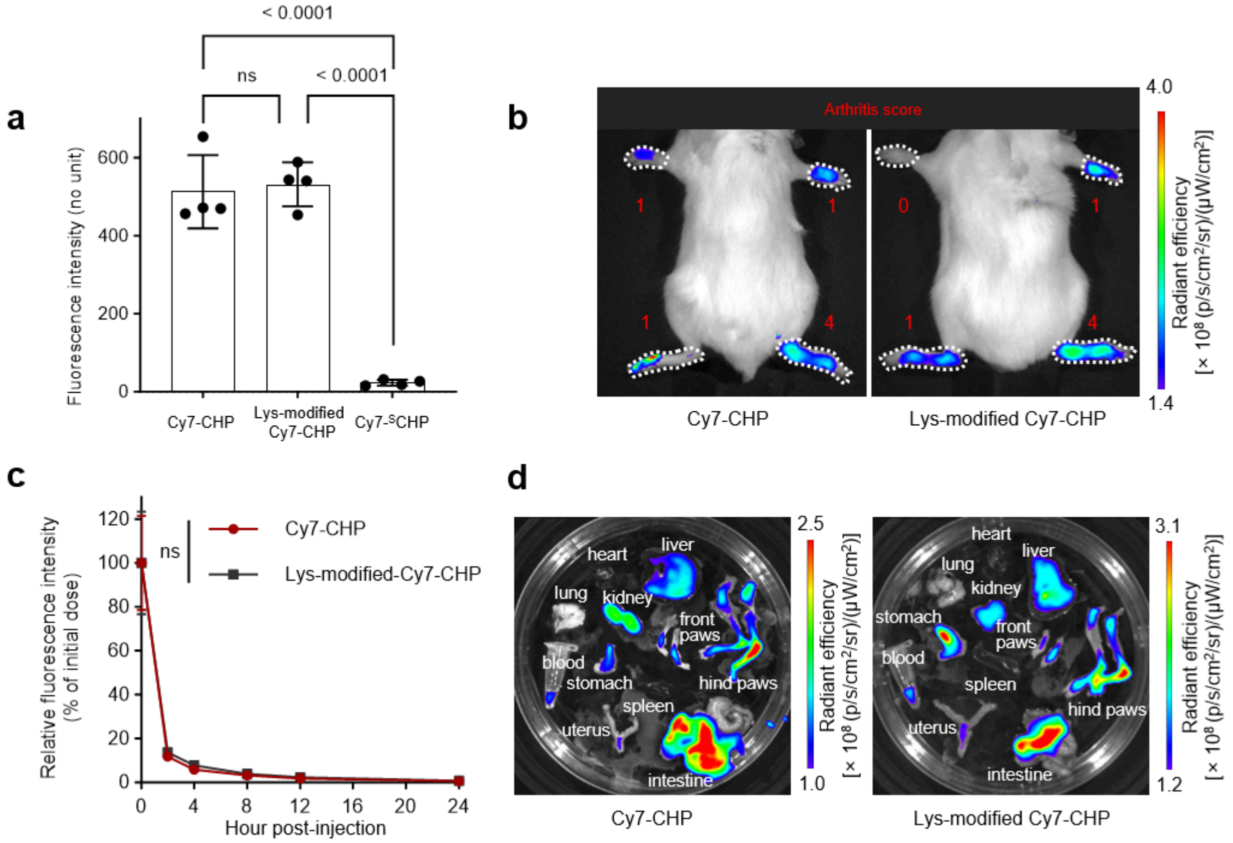

Supplementary Fig 5. **a** Fluorescence gelatin binding experiments. Cy7-^{S}CHP: sequence-scrambled control peptide. **b** Representative in vivo images of CAIA mice showing each paw's fluorescence uptake of Cy7-CHP or Lys-modified-Cy7-CHP [sequence: Cy7-Ahx-K-Ahx-$(GfO)_9$, utilized for Fig. 1b] 2 h post i.v. injection of 1 nmole of probes. **c** Fluorescence intensity of Cy7-CHP or Lys-modified-Cy7-CHP in the plasma of CAIA mice (tested on day 6 post-LPS injection) from 0.2 to 24 h post-injection, normalized to the data at 0.2 h. **d** Representative fluorescence images of the major organs, paws, and blood of CAIA mice (tested on day 6 post-LPS injection) 2 h post i.v. injection of 1 nmole Cy7-CHP or Lys-modified-Cy7-CHP. The fluorescence signals observed in the stomach and intestine originate from the autofluorescence of the chlorophyll-containing diet. Data points: individual wells (**a**) or individual mice (**c**, n = 3). Numbers were expressed as mean ± standard deviation (**a**, **c**). Statistical analysis: one-way ANOVA with Tukey's test (**a**), paired *t*-test (**c**). ns: no significance.

Systematic comparison of Cy7-CHP and Lys-modified Cy7-CHP:

- *In vitro gelatin binding:* The gelatin (heat-denatured collagen) binding assay demonstrates that both Cy7-CHP and Lys-modified Cy7-CHP possess high affinity for denatured collagen, with no significant difference in binding capacity (Supplementary Fig. 5a).
- *In vivo imaging:* Following tail vein injection in CAIA mice, both probes exhibited prominent enrichment in the inflamed paws. The fluorescence intensities at the 2 h time point were nearly identical in the RA joints (Supplementary Fig. 5b).
- *Pharmacokinetics:* Blood fluorescence analysis across multiple time points showed that the in vivo clearance profiles of Cy7-CHP and Lys-modified Cy7-CHP are almost overlapping. (Supplementary Fig. 5c).
- *Biodistribution*: Tissue harvesting 2 h post-injection revealed that the signals for both probes were primarily found in the inflamed joints and kidneys, with highly consistent biodistribution patterns (Supplementary Fig. 5d).

Summary: These data collectively demonstrate that the addition of a single lysine residue does not alter the *in vivo* targeting efficiency, the pharmacokinetics and distribution of the CHP probe.

Rationale for using the Lys-modified variant in tissue clearing and lightsheet imaging (Fig. 1b):
While the two probes behave identically in living animals, the Lys-modified Cy7-CHP offers a significant technical advantage for Light-sheet fluorescence microscopy. During the tissue clearing

process, samples are fixed with paraformaldehyde (PFA). The ε-amino group of the lysine residue in the modified probe reacts with PFA and neighboring protein amines, effectively locking the probe into the pathological matrix via covalent cross-linking. This prevents the washout of the probe during the harsh chemical environment and the slow processes of tissue clearing.

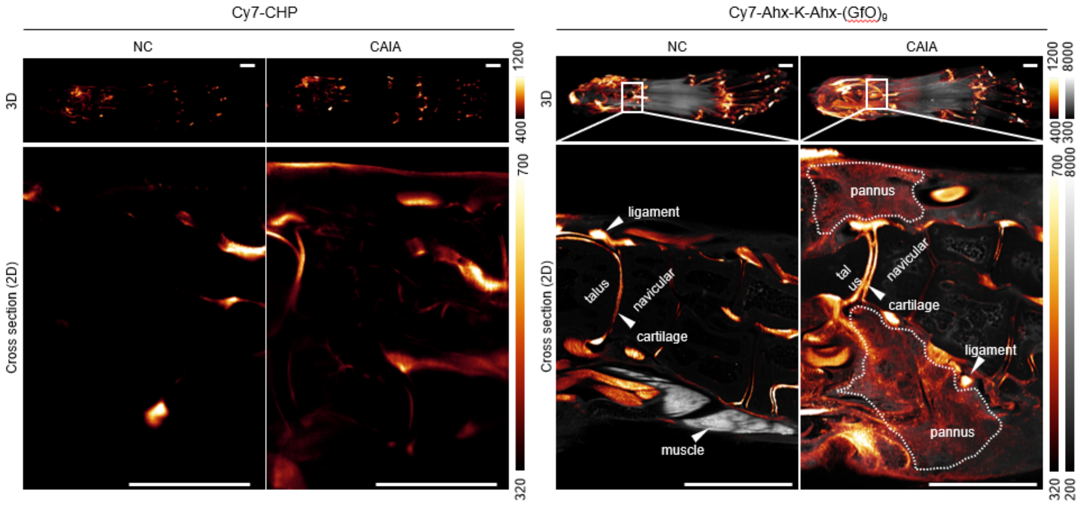

As shown in our comparative lightsheet fluorescence imaging, the Lys-modified Cy7-CHP group (right) exhibits significantly higher fluorescence intensity, superior contrast, and richer structural detail compared to the non-lysine version under the same thresholds (left, data obtained during preliminary trials of this experiment).

Therefore, while the standard Cy7-CHP is sufficient for live imaging and pharmacokinetics studies, the Lys-modified version is necessary to achieve the high-resolution visualization required for 3D light-sheet imaging. We have clarified this distinction and the supporting validation in the revised Results to ensure full transparency.

To track the exact anatomic location of CHP binding in the inflamed ankles, we used light-sheet fluorescence microscopy to scan the mice's hind paws, which were dissected 2 h post i.v. injection of a Lys-modified Cy7-CHP [Cy7-Ahx-K-Ahx-(GfO)$_9$] followed by tissue clearing using the PEGASOS method[40] (Fig. 1b, Supplementary Fig. 4, see methods). The additional Lys residue provides a primary amine group that crosslinks with paraformaldehyde during fixation. This covalent stabilization enables the CHP probe, which otherwise associates with tissue non-covalently, to withstand rigorous washing and clearing protocols. Notably, the probe's specificity and in vivo behavior remained unaltered following the Lys modification (Supplementary Fig. 5). Strong smoke-

Updates in Methods:

For Supplementary Fig. 2d and Supplementary Fig. 5c , CAIA mice (average total arthritis score per mouse: 6.3, $n = 3$ mice) and normal control mice ($n = 3$ mice) received a single i.v. injection of 1 nmole of Cy7-CHP, (or Lys-modified-Cy7-CHP). Blood was collected at 0.2, 2, 4, 8, 12, and 24 h. Plasma samples were analyzed using the IVIS Spectrum system under the same imaging settings as described above.

interest for each organ/tissue measured across all mAb-dosed mice using the Living Image software after removing the value of the same organ/tissue of a CAIA mouse without any mAb injection. For Supplementary Fig. 5d, CAIA mice (average total arthritis score per mouse: 6.5, $n = 2$ mice) received a single i.v. injection of 1 nmole of Cy7-CHP or Lys-modified-Cy7-CHP. The mice were sacrificed at 2 h post-injection, and the same set of tissues was collected and imaged.

Gelatin binding assays

For Supplementary Fig. 5a, wells of a 96-well plate were coated with approximately 6 µL of 70 °C hot gelatin (Sigma, G2500) solution in PBS (10% w/v) before incubation at 4 °C for 10 min to allow gelation. PBS solution (100 µL) containing 10 µM of Cy7-CHP or Lys-modified-Cy7-CHP [Cy7-Ahx-K-Ahx-$(GfO)_9$] or Cy7-^{S}CHP was heated at 45 °C for 10 min before being immediately added to each well and allowed to bind at 4 °C overnight ($n = 4$). The plate was washed three times with 200 µL of PBS at room temperature. The fluorescence intensity of each well was detected with a Biotek Synergy HTX plate reader (ex/em: 750/800 nm, gain: 1000).

4. Further, for the antibody conjugate, tissue was collected at 2 hours post injection (for data discussed staring line 308, line 312). The authors show that the antibody conjugate has slow clearance compared to CHP-Cy7, so at 2 hours post injection wouldn't there be significant non-specific binding of the probe?

The authors should show clearly the clearance half-lives of both the CHP-7 and the Cy7-labeled TNFmAb-CHP from the blood of NC and both models of CAIA models, as well the normal/uninjured paw and the CAIA paws in both models. It is not clear how the timepoint for imaging and/or tissue harvest were determined.

Original text, lines 308–312

Anatomical locating CHP-delivered TNFmAb in the arthritic paws

We traced the distribution of the CHP-conjugated TNFmAb-CHP within the anatomical locations of the inflamed hind paws of the CAIA mice using light-sheet microscopy. On day 2 post-LPS-induction of arthritis in the right hind footpad (Fig. 5a, arthritis score: 2), both mice were intravenously injected with an equal dose of Cy7-labeled TNFmAb-CHP or TNFmAb. Two hours post-injection, their hind

We appreciate the reviewer's concern regarding potential non-specific signals due to the slow clearance profile of antibody conjugates. The reviewer is correct that at 2 hours post-injection, the conjugate is still circulating in the bloodstream. However, we selected this specific time point for lightsheet fluorescence microscopy for the following reasons:

- *Optimization of the comparative window:* A central goal of this study was to compare the TNFmAb-CHP conjugate with the unmodified TNFmAb. Because the control molecules clear more rapidly than the conjugate, waiting until the 6-hour peak would result in a significant loss of signal in the control groups. The 2-hour window provides a robust and sufficient signal for the conjugate while ensuring the control group is still detectable, allowing for a rigorous head-to-head spatial comparison.

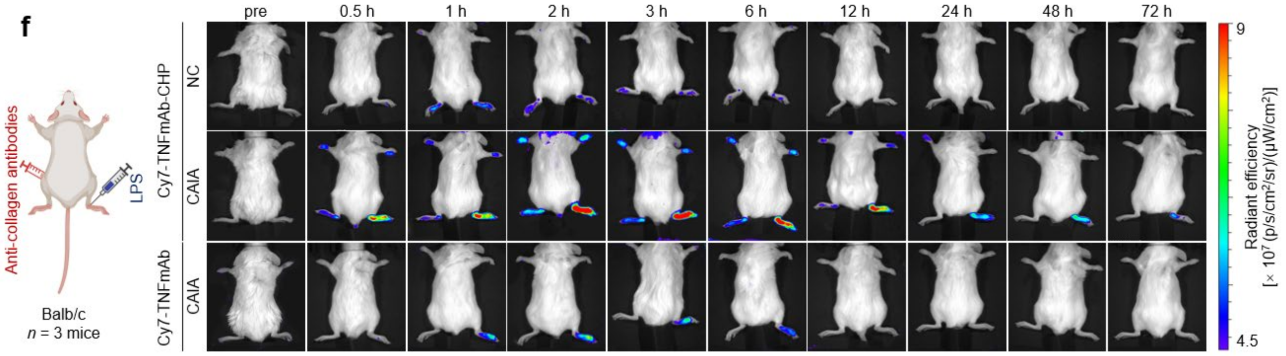

- *Spatial discrimination versus total signal:* While non-specific circulating signal may be present at 2 hours, lightsheet fluorescence microscopy provides the spatial resolution necessary to distinguish between intravascular signal and specific tissue/matrix binding. Our analysis focuses on the distinct patterns of localization of the two mAb molecules within those joints rather than total fluorescence intensity alone.
- *Systemic perfusion to eliminate blood pool signal:* Crucially, as we stated in the Methods, prior to tissue collection at the 2-hour mark, all animals underwent systemic perfusion with PBS to flush the entire vasculature. This step helps remove the majority of circulating, non-bound fluorescent mAbs in our lightsheet fluorescence microscopy images (Fig. 5).

We would also like to further clarify that while the 2-hour post-injection time point was used for both the Cy7-CHP peptide (Fig. 2) and the mAb-CHP conjugate (Fig. 5), the scientific rationale behind this selection differs based on the unique pharmacokinetic profiles of the two molecules:

- *For Cy7-CHP (peptide-based imaging):* The 2-hour window was chosen because this small peptide clears rapidly from the systemic circulation. By 2 hours, the non-specifically bound or circulating background probes have been largely eliminated. Thus, the remaining fluorescence signal represents the probe's specific binding to damaged collagen, ensuring the highest diagnostic accuracy and sensitivity for RA detection (the purpose of Fig. 2).
- *For TNFmAb-CHP (antibody-based distribution):* In contrast, the antibody conjugate has a significantly longer half-life and slower clearance. For this molecule, the 2-hour time point was selected not to wait for total systemic clearance, but to **capture the high accumulation** in the target tissue as indicated by in vivo imaging (Fig. 3f). This allows for a high-resolution 3D visualization of the spatial distribution patterns of the two mAb-based therapeutics.

According to your suggestions, we have made the following updates to the manuscript.

- We have added a brief justification to the corresponding Results section regarding the selection of the 2 h post-injection time point for imaging and tissue-harvesting.

We traced the distribution of the CHP-conjugated TNFmAb-CHP within the anatomical locations of the inflamed hind paws of the CAIA mice using light-sheet microscopy. On day 2 post-LPS-induction of arthritis in the right hind footpad (Fig. 5a, arthritis score: 2), both mice were intravenously injected with an equal dose of Cy7-labeled TNFmAb-CHP or TNFmAb. Two hours post-injection, their hind paws were collected and cleared. The 2 h time point was selected to ensure a robust fluorescence signal for the Cy7-TNFmAb-CHP conjugate while maintaining detectable signal levels in the more rapidly cleared Cy7-TNFmAb control group in the RA joint (Fig. 3f), thereby enabling a direct and meaningful spatial comparison. To eliminate non-specific signals from the circulation, all animals were subjected to extensive systemic perfusion with PBS prior to tissue collection, ensuring that the detected fluorescence specifically represents tissue-localized mAbs. Light-sheet fluorescence imaging revealed

- In the text related to Fig. 1, where the initial in vivo fluorescence imaging of Cy7-CHP is described, we have added a sentence justifying the 2 h interval based on its rapid clearance profile.

cartilage degradation and bone erosion (Supplementary Fig. 1b)[39]. Two hours post intravenous (i.v.) injection of Cy7-labeled CHP, drastically stronger in vivo near-infrared fluorescence signals were detected in the swollen ankle regions in the hind paws of the CAIA mice (6 days post-disease onset, hind paw arthritis score = 4) than those of the normal naïve mice (Fig. 1a and Supplementary Fig. 2). In vivo imaging was performed at 2 h post-injection of Cy7-CHP to allow for the systemic clearance of non-specific circulating probes (Supplementary Fig. 2d), thereby ensuring that the remaining fluorescence specifically highlights collagen damage for accurate RA detection (Supplementary Fig. 2a). However, the control peptide Cy7-^{S}CHP with a scrambled amino acid sequence and no collagen

- Following your suggestion, we assessed the clearance profiles via serum concentrations for Cy7-CHP (Supplementary Fig. 2d), as well as for Cy7-TNFmAb and Cy7-TNFmAb-CHP in both NC (Supplementary Fig. 32a) and the systemic CAIA mice (Fig. 6c).

Supplementary Fig. 2d

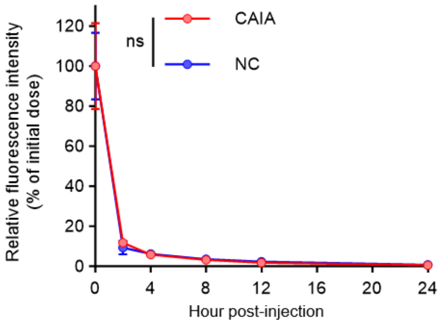

Fig. 6c

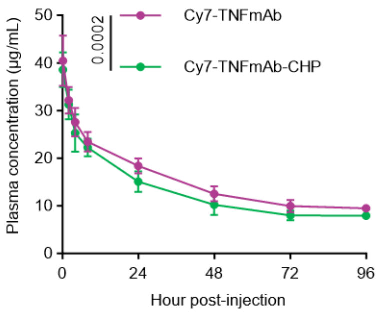

Supplementary Fig. 31a

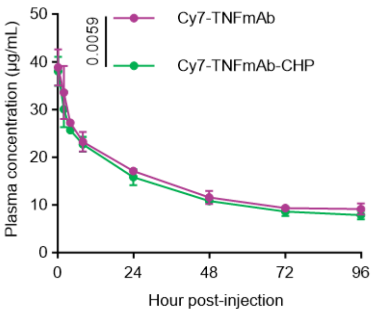

Supplementary Fig. 2d Fluorescence intensity of Cy7-CHP in plasma from NC and CAIA mice (tested on day 6 post-LPS injection) from 0.2 to 24 h post-injection, normalized to the data obtained at 0.2 h (n = 3 mice). Statistical analysis: paired t-test (**d**).
Fig. 6c Quantification of the Cy7-TNFmAb and Cy7-TNFmAb-CHP concentrations in the plasma of the CAIA mice at different time points post-injection.
Supplementary Fig. 31a Fluorescence based quantification of Cy7-TNFmAb and Cy7-TNFmAb-CHP concentrations in the plasma of NC mice at different time points post i.v. injection (dose: 30 μg).

- Blood half-lives calculated by nonlinear regression using a two-phase decay model are summarized below.

Compound	Cy7-CHP		Cy7-TNFmAb-CHP		Cy7-TNFmAb	
Model	NC	CAIA	NC	CAIA	NC	CAIA
Half-life (h)	0.60	0.52	19.57	18.45	22.16	25.06

- Finally, we would like to clarify that determining accurate local half-lives specifically within “normal versus CAIA paws” is technically unfeasible. Due to the limited penetration depth of fluorescence and varying optical properties of the tissue, it is not possible to achieve the reliable, absolute quantification of probe concentrations required to calculate local pharmacokinetic parameters in these specific anatomical regions.

5. Line 348: the authors say that the CHP-TNFmAb conjugate has a shorter blood half-life than the other Cy7-TNFmAB conjugate. How can this be explained? The pharmacokinetics would be primarily governed by the large size of the antibody, and introduction of CHP would only serve to increase the size of the probe which would have either minimal effect of blood half-life or prolong blood half-life. What is the blood half-life of a scrambled CHP-antibody conjugate?

Original lines 348-352:

CAIA mice 0.2–96 h post i.v. injection (Fig. 6a, b). The plasma concentration of Cy7-TNFmAb-CHP declined quickly from 2 h and approached zero by 96 h, whereas the concentration of Cy7-TNFmAb was notably higher from 2 h (Fig. 6b, c). Indeed, with the identical dosage, the area under the concentration-time curve for Cy7-TNFmAb-CHP was merely 28.2% that of TNFmAb within 96 h post-injection (Fig. 6d), suggesting that CHP-conjugation may significantly reduce the systemic exposure of TNFmAb in CAIA mice.

Original Figs. 6b–d

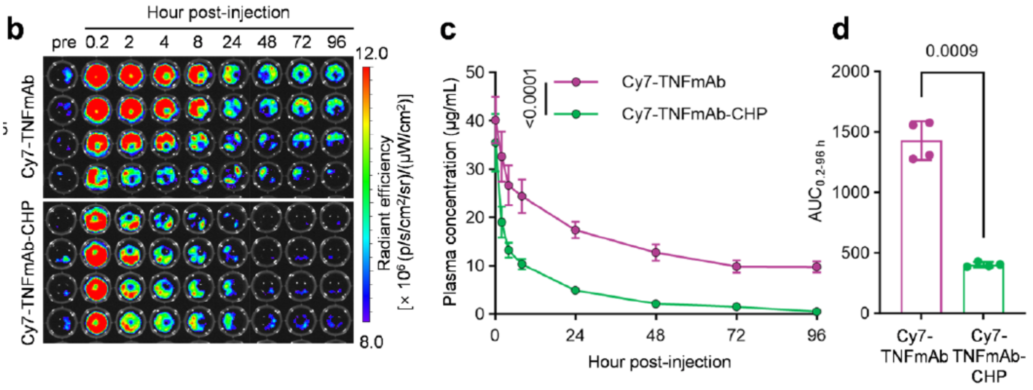

b Fluorescence intensity of the Cy7-TNFmAb or Cy7-TNFmAb-CHP containing plasma (50 μL/well) at different time points. **c** Quantification of the Cy7-TNFmAb and Cy7-TNFmAb-CHP concentrations in the plasma of the CAIA mice in (**b**). **d** Areas under the curve (AUC) of the plasma concentration-time curves for Cy7-TNFmAb and Cy7-TNFmAb-CHP from 0.2 to 96 h post-injection.

We sincerely thank you for this profound and critical observation. We agree that the initially observed discrepancy, where the serum concentration AUC of Cy7-TNFmAb-CHP was merely 28.2% of that of TNFmAb, was indeed unexpected and inconsistent with the molecules' intended behavior.

Following a systematic investigation and validation process, we discovered that this anomaly was caused by a degree of micro-aggregation in the specific batch of Cy7-TNFmAb-CHP (but not Cy7-TNFmAb) used for the original Fig. 6. This aggregation most likely occurred during several months of storage prior to those specific experiments.

Below is the detailed analysis, the solution implemented, and the updated data.

(3) Evidence of Cy7-TNFmAb-CHP conjugate aggregation

- *Dynamic Light Scattering (DLS) Analysis:* We performed size analysis on the clear sample solutions at 37 °C. The results showed that while Cy7-TNFmAb exhibited a single peak at nearly 10 nm, the Cy7-TNFmAb-CHP batch showed a new peak at 100 nm in addition to the primary 10 nm peak. This confirms the presence of partial antibody aggregation.

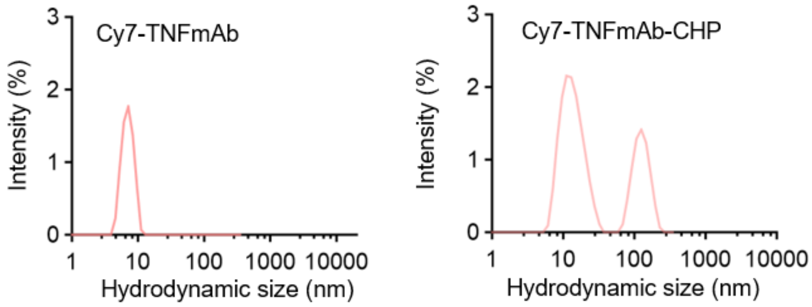

DLS analysis confirmed partial aggregation of Cy7-TNFmAb-CHP compared to Cy7-TNFmAb. (Both samples were measured in PBS at a concentration of 25 μg/mL at 37 °C.)

- *Impact on pharmacokinetics:* Protein aggregates are known to be cleared more rapidly from the bloodstream by the reticuloendothelial system, which significantly shortens their half-life. This explains why the Cy7-TNFmAb-CHP signal in the original Fig. 6 declined much faster than Cy7-TNFmAb, leading to the low AUC ratio.
- *Reversibility:* DLS measurements demonstrated that heating the affected Cy7-TNFmAb-CHP solution to 55 °C for 5 minutes caused the 100 nm peak to disappear. This indicates that the aggregation was reversible under mild thermal treatment.

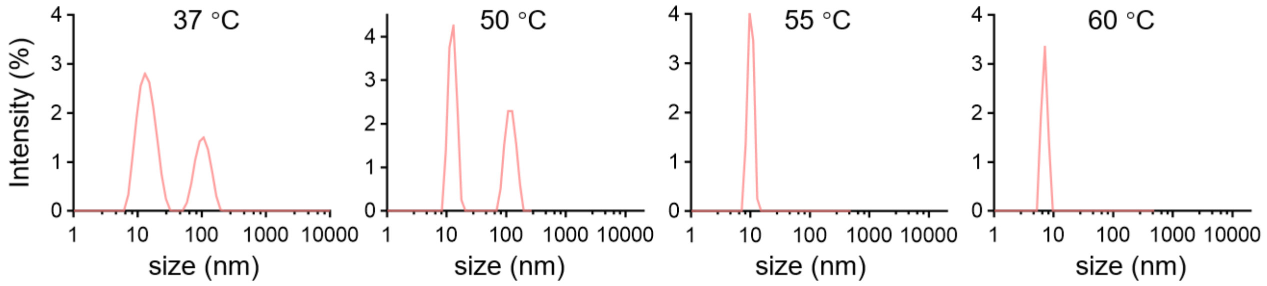

DLS measurements of Cy7-TNFmAb-CHP particle size after heating at different temperatures for 5 min showed that disaggregation of Cy7-TNFmAb-CHP aggregates began upon heating at 55 °C for 5 min. All samples were measured in PBS at a concentration of 25 μg/mL.

(4) Scope of the issue

Crucially, the unlabeled TNFmAb and TNFmAb-CHP used for all therapeutic efficacy experiments (e.g., Fig. 4) did *not* undergo aggregation. Their experimental results remain valid and unaffected. The issue was strictly confined to the *fluorescently labeled* batches used for the metabolic and imaging studies (Figs. 3 and 6).

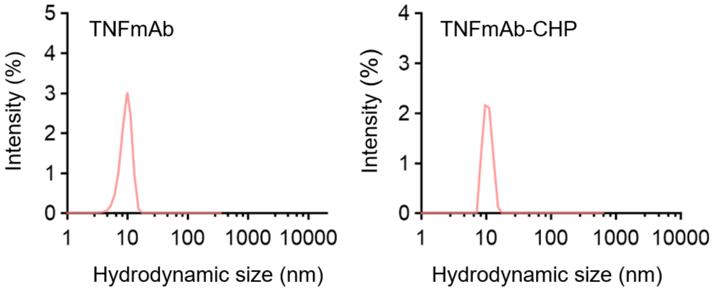

DLS analysis confirmed the absence of aggregation for unlabeled Cy7-TNFmAb and Cy7-TNFmAb-CHP. Both samples were measured in PBS at a concentration of 25 μg/mL and a temperature of 37°C.

(3) Data rectification and updated results

Based on these findings, we have repeated and re-collected all in vivo experimental data involving Cy7-TNFmAb-CHP.

We synthesized a fresh batch of Cy7-TNFmAb-CHP and confirmed the absence of protein aggregation via DLS.
In CAIA mice, the new batch of Cy7-TNFmAb-CHP exhibited a serum concentration trend similar to that of Cy7-TNFmAb (see New Figs. 6b–d). The AUC ratio improved significantly from 28.2% to 85.4%.

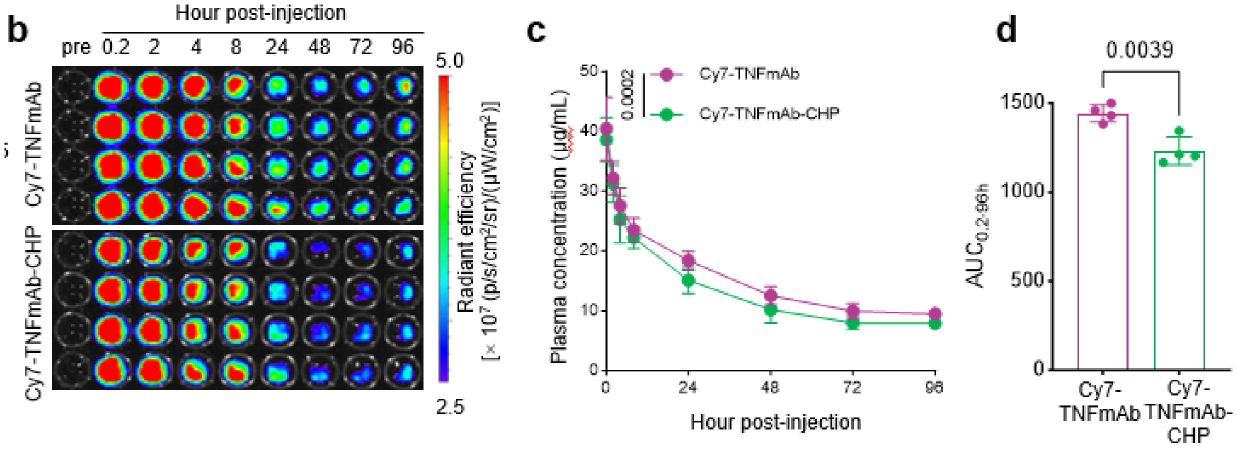

(updated) Fig 6 b Fluorescence intensity of the Cy7-TNFmAb or Cy7-TNFmAb-CHP containing plasma (50 µL/well) at different time points. **c** Quantification of the Cy7-TNFmAb and Cy7-TNFmAb-CHP concentrations in the plasma of the CAIA mice in (**b**). **d** Areas under the curve (AUC) of the plasma concentration-time curves for Cy7-TNFmAb and Cy7-TNFmAb-CHP from 0.2 to 96 h post-injection.

This confirms that the initial low AUC was due to the rapid clearance of aggregates, rather than an inherent, unexpected change in the conjugate's properties.
We have also updated the biodistribution data in **Figs. 6e & 6f** and the in vivo targeting data in **Figs. 3f & 3g** using the new batch of Cy7-TNFmAb-CHP conjugate. Detailed responses regarding these updates can be found in our replies to Comments 9 and 10 of Reviewer 1 above.

(4) Pharmacokinetics data in healthy mice
Regarding your question, *"Is there PK data available for the 2 compounds in healthy mice?"*, we have performed the requested experiments.
The results show that the plasma concentration curves for Cy7-TNFmAb and Cy7-TNFmAb-CHP are even more closely aligned in normal control mice, with no significant difference in the AUC. These data have been added to the Supplementary Information.

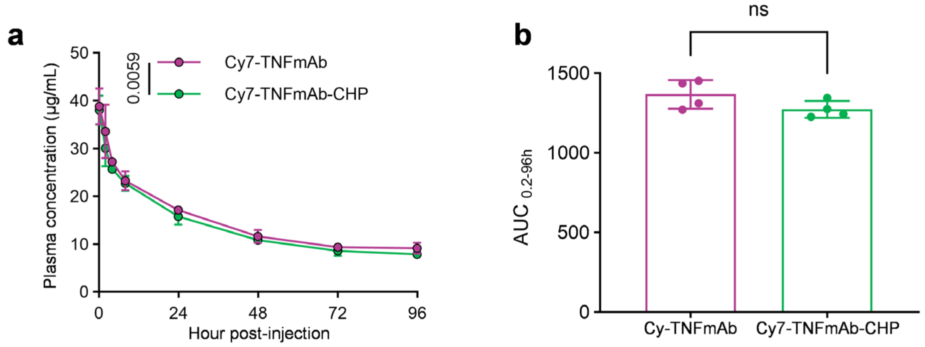

Supplementary Fig. 31. a Fluorescence based quantification of Cy7-TNFmAb and Cy7-TNFmAb-CHP concentrations in the plasma of NC mice at different time points post i.v. injection (dose: 30 µg). **b** AUC of the plasma concentration-time curves for Cy7-TNFmAb and Cy7-TNFmAb-CHP from 0.2 to 96 h post-injection. Statistical analysis: paired *t*-test (**a**) and unpaired *t*-test (**b**). ns: no significance.

In addition to updating the figures, we have revised the corresponding figure descriptions, results and discussions in the manuscript to reflect these corrected findings.
Results:

Pharmacokinetics and biodistribution of Cy7-TNFmAb-CHP in CAIA mice

Finally, we characterized the longitudinal blood concentrations of Cy7-TNFmAb-CHP and Cy7-TNFmAb via Cy7 fluorescence measurements of serial blood samples collected from CAIA mice 0.2–96 h post i.v. injection (Fig. 6a, b). Both probes exhibited a similar gradual decline, approaching a plateau by 96 h (Fig. 6b, c). At equivalent dosing, the area under the concentration-time curve for Cy7-TNFmAb-CHP was approximately 85.4% of that observed for TNFmAb within 96 h post-injection (Fig. 6d). Interestingly, the pharmacokinetic profiles of the two constructs were nearly indistinguishable in healthy control mice (Supplementary Fig. 31). This suggests that the modest reduction in serum concentration observed for Cy7-TNFmAb-CHP in CAIA mice (Fig. 6c, d) may be partially attributable to its enhanced retention within inflamed RA joints.

We further assessed the biodistribution of Cy7-TNFmAb-CHP and Cy7-TNFmAb in major organs of CAIA mice 24 h post-injection via fluorescence imaging (Fig. 6e). Aside from the inflamed paws, the Cy7 fluorescence intensities from Cy7-TNFmAb-CHP and Cy7-TNFmAb were comparable across all harvested organs and tissues (Fig. 6f). Similar biodistribution profiles were observed at 2 h post-injection (Supplementary Fig. 32).

To investigate the in vivo toxicity of the TNFmAb-CHP conjugate, we also analyzed the lungs, livers, spleens, kidneys, and hearts of all the mice at the endpoint of the efficacy study (Fig. 4a). Histology of the organs from the TNFmAb-CHP-treated mice showed intact cellular and tissue architecture without apparent inflammation, hemorrhage, or necrosis, comparable to that of the TNFmAb and normal groups (Supplementary Fig. 33). Collectively, these data suggest that CHP conjugation enhances the therapeutic index of TNFmAb by facilitating targeted joint retention without significantly altering systemic clearance or inducing off-target organ toxicity.

Updates in Discussion:

the superior efficacy of TNFmAb-CHP[56]. Furthermore, our study demonstrates the unique utility of light-sheet microscopy in visualizing the in vivo spatial distribution of therapeutics across tissue architectures[64]. While CHP conjugation led to a modest reduction in systemic exposure (Fig. 6d), it facilitated a preferential accumulation of TNFmAb within the inflamed ECM of RA joints (Fig. 6f). This subtle shift in biodistribution suggests a promising strategy for improving the therapeutic index and reducing the potential for systemic side effects (Fig. 6). We also provide preliminary evidence that the collagen-hybridizing targeting strategy can be applied to different RA animal models and possesses potential for clinical translation (Supplementary Figs. 29-30).

6. Why are females only used in this study?

We sincerely thank you for this important question regarding sex as a biological variable. The decision to use only female mice for the CAIA model in this study was based on a combination of clinical relevance, model reliability, and established experimental protocols in the field of RA research.

Here is a summary of the reasons for this choice, which we have now clarified in the Methods section:

1) Clinical relevance to human disease

Rheumatoid arthritis is a highly sexually dimorphic disease in humans. Epidemiological data consistently show that RA is significantly more prevalent in women than in men, with a ratio of approximately 2–5:1. [Ref: Volkmann, E.R., Feghali-Bostwick, C. Sex- and gender-based personalized medicine in rheumatology. *Nat Rev Rheumatol* **21**, 251–252 (2025)]. Furthermore, women often experience more severe symptoms and a different disease progression. By using female mice, we aimed to align our animal model with the most representative patient demographic affected by this condition.

2) Model susceptibility and uniformity

In the context of the CIA (Collagen-Induced Arthritis) and CAIA (Collagen Antibody-Induced Arthritis) models, female mice are widely recognized for their consistent susceptibility and more predictable disease kinetics.

3) Ethical and practical considerations

Male mice housed together are prone to aggressive behavior and fighting, especially when under the stress of systemic inflammation. Fighting often leads to skin lesions and secondary inflammation in the paws and tail. These injuries can interfere with the objective clinical scoring of arthritis and create false positive signals in fluorescence imaging. The increased stress levels in aggressive male groups can also alter immune responses, potentially confounding the therapeutic efficacy data.

4) Consistency with existing literature

The vast majority of studies involving the evaluation of antibody-based RA therapeutics and collagen-targeting probes have utilized female mice. Using females allowed us to directly compare our results with previously published benchmarks, ensuring our findings are interpreted within a well-established scientific context.

Finally, we have added a clarifying statement in the CAIA Models subsection of the Methods to explain this rationale:

CAIA Models

Female Balb/c mice (7 to 8 weeks old) were purchased from SPF (Beijing) Biotechnology, and housed in a specific-pathogen-free (SPF) grade facility with a 12-hour cycle of light and darkness with free access to food and water. Consistent with established protocols for the CAIA model, female mice were used in this study to reflect the higher clinical prevalence of RA in women and to minimize the confounding effects of male aggression on arthritis scoring. Arthritis was induced by intraperitoneal

7. Were the arthritis scores blinded?

Yes, the arthritis scoring was performed in a blinded manner.

Throughout the study, the investigator responsible for assessing and recording the paw swelling and arthritis scores was unaware of the group allocation (e.g., whether the mouse received TNFmAb or TNFmAb-CHP). This standard blinding procedure was implemented to prevent any potential observer bias during data collection.

We have updated the Methods section of the revised manuscript to explicitly state this blinding requirement:

their average arthritis scores progressed to 1 or 2 per paw (from 1 or 2 days post-LPS induction), where paw swelling was monitored daily for 7 days post-treatment (Supplementary Figs. 24-25). All paw scoring and measurements were conducted in a blinded manner, where the investigator responsible for arthritis scoring was kept unaware of the specific treatment group allocations.

RESPONSE TO REVIEWERS' COMMENTS

Reviewer #1 (Remarks to the Author):
The authors have addressed all the comments very well and have performed valuable additional experiments. The correction of the experiments in which they previously identified microaggregates, is in particular very relevant.
I have no further comments.
Reviewer #2 (Remarks to the Author):
I co-reviewed this manuscript with one of the reviewers who provided the listed reports. This is part of the Nature Communications initiative to facilitate training in peer review and to provide appropriate recognition for Early Career Researchers who co-review manuscripts.
We thank the reviewers for the positive feedback and support for our manuscript.

Reviewer #3 (Remarks to the Author):
In the revision of the manuscript titled “Imaging and Therapeutic Antibody Delivery in Rheumatoid Arthritis by Collagen Hybridization” Chen et al did an admirable job addressing all the reviewer comments. Based on this and the additions to the manuscript, my recommendation is to publish the manuscript after addressing these final minor issues:
We thank the reviewer for the thoughtful evaluation and for recommending the manuscript for publication. We appreciate the recognition of our additional experimental efforts.

1. For Supplementary Figure 13, please show the time points where there are significant differences between the groups.

Original Supplementary Fig. 13

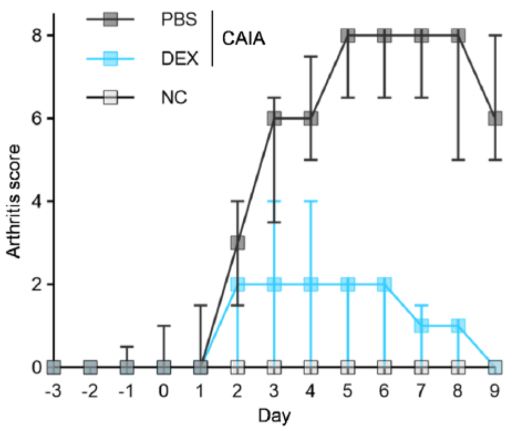

Supplementary Fig. 13. Arthritis scores of the hind paws of the NC mice or the CAIA mice treated with DEX or PBS throughout the study in Fig. 2f. Numbers were expressed as median with interquartile range.

We thank the reviewer for the thoughtful suggestion. We have carefully evaluated the feasibility of performing day-by-day comparisons for Supplementary Figure 13. However, considering the nature of the data and the necessity for statistical rigor, we have opted to perform the holistic analysis instead for the following reasons:

- **Data:** The arthritis score is an ordinal (rank-based) variable rather than a continuous one. Therefore, parametric tests like 2-way ANOVA are not applicable. To maintain statistical integrity, we must rely on non-parametric methods.

- **Multiplicity:** Our study records data daily over 13 days. Performing independent non-parametric comparisons (such as the Kruskal-Wallis test) for each of the 13 time points would necessitate a very stringent multi-comparison correction (e.g., Bonferroni correction). This would significantly reduce statistical power and increase the risk of Type II errors, potentially masking the true therapeutic effect of DEX that is clearly visible in the overall trend.
- To account for the *repeated-measure* nature of the data, we utilized the Friedman test for repeated measures on ranks followed by Dunn's multiple comparisons test. This approach is specifically designed for ordinal data in a longitudinal study, allowing us to compare the overall disease progression and treatment efficacy across the three groups (NC, CAIA-PBS, and CAIA-DEX) throughout the entire 13-day course.

The results of the Friedman test confirm that the CAIA-DEX group exhibited a significantly lower overall arthritis burden compared to the CAIA-PBS group (P < 0.05). We believe this added global analysis provides a more robust and statistically sound conclusion regarding the drug's efficacy than isolated daily comparisons.

We have updated the figure and figure legend of Supplementary Figure 13 to explicitly state the use of this non-parametric repeated-measures analysis. We hope the reviewer finds this explanation and our rigorous statistical approach satisfactory.

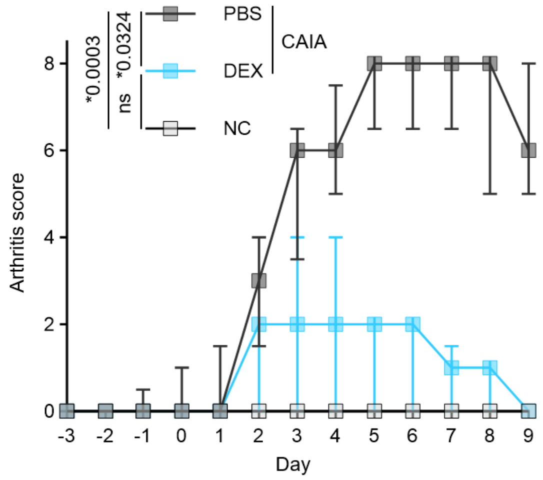

(updated) Supplementary Fig. 13. Arthritis scores of the hind paws of the NC mice or the CAIA mice treated with DEX or PBS throughout the study in Fig. 2f. Data were expressed as median with interquartile range. Statistical analysis: Friedman test for repeated measures on ranks and Dunn's multiple comparisons.

2. Comment 5: Can you please show quantification of the DLS data? It looks to me like the CHP conjugate is much larger in size than the non-CHP conjugate, but it is unclear if this is within experimental error or not. Does the aggregation happen with the other CHP conjugates? Have the authors verified that their other probes with CHP do not aggregate? How is this verified, and is the verification done before every experiment?

Comment 5 in the R1 response letter.

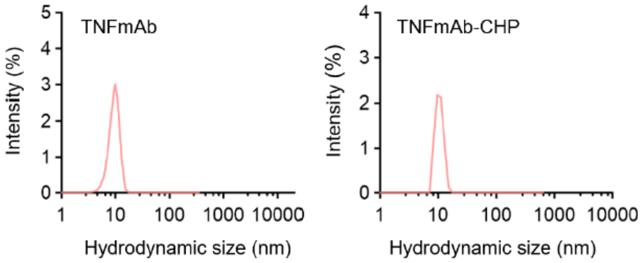

DLS analysis confirmed the absence of aggregation for unlabeled TNFmAb and TNFmAb-CHP. Both samples were measured in PBS at a concentration of 25 µg/mL and at 37 °C.

We thank the reviewer for this rigorous observation and for the opportunity to clarify the DLS results. We have performed a detailed quantification of the DLS data to address this concern. Our analysis confirms that the hydrodynamic size of TNFmAb after CHP conjugation is essentially identical to that of the unmodified TNFmAb. The quantitative results are summarized below:

	TNFmAb	TNFmAb-CHP
Mean peak diameter (nm)	10.01 ± 1.95	10.67 ± 1.78
Data plot (from comment 5 in R1 response letter)	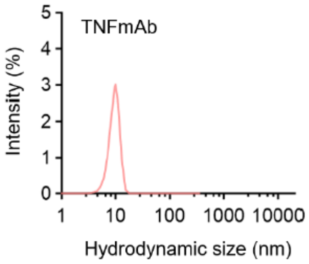	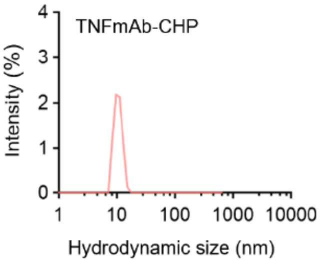

The difference in the mean hydrodynamic diameter between the two groups is 0.66 nm, which falls well within the distribution width of the peaks and the inherent instrumental error of DLS for protein-sized molecules. Each plot represents the statistical average of n=5 measurements per sample, and these results were consistent across multiple independent biological replicates.

We believe the perception that the TNFmAb-CHP conjugate appears “larger” is likely a visual artifact arising from the logarithmic scale of the X-axis. On a $\log_{10}$ scale, the interval between 1 nm and 10 nm is visually stretched compared to the interval between 10 nm and 20 nm. Consequently, the slightly wider distribution of the TNFmAb peak (diameter range: 10.01 ± 1.95 nm) causes it to spread further toward the left (lower values) on the axis. In contrast, the TNFmAb-CHP peak is narrower (diameter range: 10.67 ± 1.78 nm) and appears more compact. This increased “sharpness”, combined with the minor 0.66 nm shift in the mean, creates a visual illusion of a more significant size increase than what is numerically present.

The 0.66 nm difference is negligible in the context of protein hydrodynamic diameter measurements and does not represent a biologically or physically significant change in size. To further demonstrate this, we have provided an updated version of the size distribution plot using a *linear* X-axis (see below), which clearly shows that the two distributions almost entirely overlap.

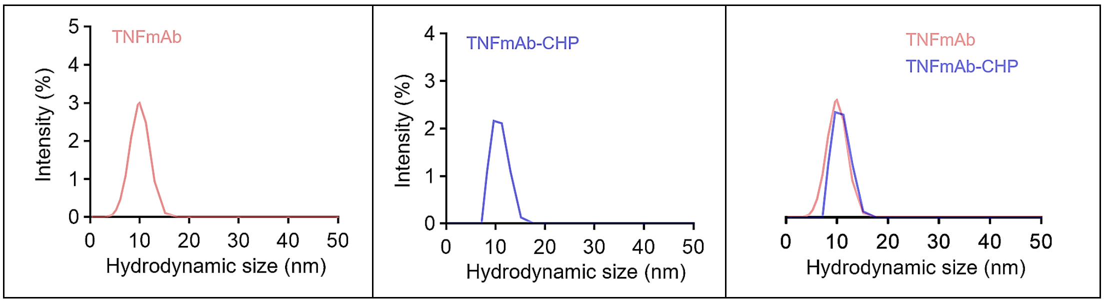

Does the aggregation happen with the other CHP conjugates? Have the authors verified that their other probes with CHP do not aggregate? How is this verified, and is the verification done before every experiment?

We thank the reviewer for these important questions regarding the stability of our CHP conjugates. We can confirm that the micro-aggregation previously identified was an incident confined to a specific batch of Cy7-TNFmAb-CHP. As detailed in our previous revision, all data associated with that batch have been replaced with results from non-aggregated samples. To ensure the integrity of our results, we verified the size distribution of our CHP-conjugates via DLS prior to conducting those new experiments. Our results confirm that Cy7-TNFmAb and Cy7-TNFmAb-CHP exhibit nearly identical hydrodynamic diameters, indicating no aggregation occurs upon conjugation.

	Cy7-TNFmAb	Cy7-TNFmAb-CHP	Merge
Mean peak diameter (nm)	10.34 ± 1.23	10.28 ± 1.22	
Data plot	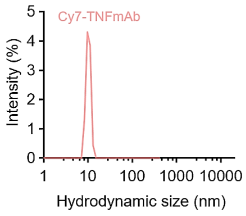	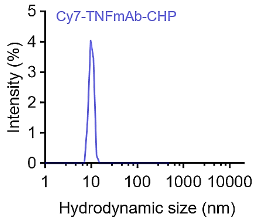	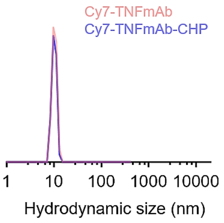

Furthermore, to demonstrate the long-term stability of these conjugates, we measured the size of TNFmAb-CHP *eight* months after its initial conjugation reaction. As shown below, the conjugate remains non-aggregated even after prolonged storage. As emphasized in our prior response, the unlabeled TNFmAb and TNFmAb-CHP used for all therapeutic efficacy tests (e.g., Fig. 4) did not undergo aggregation. We have rigorously verified these materials, and remain confident that the therapeutic results are valid and unaffected.

	TNFmAb	TNFmAb-CHP
Mean peak diameter (nm)	10.01 ± 1.95	10.67 ± 1.78
Data plot	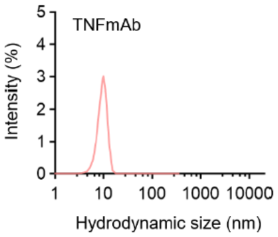	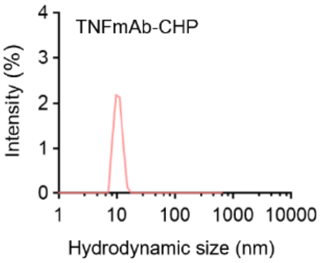
